# Molecular Mechanism of *Salvia miltiorrhiza* Bunge in Treating Cerebral Infarction

**DOI:** 10.1155/2022/5992394

**Published:** 2022-03-29

**Authors:** Xietao Ye, Jiali Liu, Xinyao Yuan, Songhong Yang, Yi Huang, Yan Chen

**Affiliations:** ^1^Affiliated Hospital of Integrated Traditional Chinese and Western Medicine, Nanjing University of Chinese Medicine, Nanjing, China; ^2^Jiangsu Province Academy of Traditional Chinese Medicine, Nanjing, China; ^3^Key Laboratory of Drug Metabolism and Pharmacokinetics, State Key Laboratory of Natural Medicines, China Pharmaceutical University, Nanjing, China; ^4^Tiantai County Food and Drug Testing Center, Taizhou, China; ^5^Jiangxi University of Chinese Medicine, Nanchang, China

## Abstract

**Background:**

Cerebral infarction (CI) is a common brain disease in clinical practice, which is mainly due to the pathological environment of ischemia and hypoxia caused by difficult cerebral circulation perfusion function, resulting in ischemic necrosis of local brain tissue and neurological impairment. In traditional Chinese medicine (TCM) theory, CI is mainly due to blood stasis in the brain. Therefore, blood-activating and stasis-dissipating drugs are often used to treat CI in clinical practice. *Salvia miltiorrhiza* Bunge (SMB) is a kind of traditional Chinese medicine with good efficacy in promoting blood circulation and removing blood stasis, and treatment of CI with it is a feasible strategy. Based on the above analysis, we chose network pharmacology to investigate the feasibility of SMB in the treatment of CI and to study the possible molecular mechanisms by providing some reference for the treatment of CI with TCM.

**Methods:**

The active ingredients and related targets of SMB were obtained through the Traditional Chinese Medicine Systems Pharmacology (TCMSP) database, and CI-related targets were obtained from the GeneCards and DisGeNET databases. The target of SMB for the treatment of CI was obtained using Cytoscape software and visualized. GO and KEGG enrichment analysis was performed based on “clusterProfiler” within R, and the prediction results were validated by molecular docking technique.

**Results:**

By constructing a compound-target (C-T) network, it was found that the active components in SMB mainly treated CI by regulating key proteins such as AKT1, IL-6, and EGFR. These key proteins mainly involve in pathways such as immune regulation, cancer and lipid metabolism, such as lipid and atherosclerosis, chemical carcinogenesis-receptor activation pathways, and IL-17 signaling pathway. In the GO term, it mainly regulates the response to steroid hormones, membrane rafts, and G protein-amine receptor coupled activity. Eventually, we verified that the luteolin and tanshinone IIA components in SMB have a good possibility of action with AKT1 and IL-6 by in silico techniques, indicating that SMB has some scientificity in the treatment of CI.

**Conclusion:**

SMB mainly treats CI by regulating 94 proteins involved in lipid and atherosclerosis, chemical carcinogenesis-receptor activation, and IL-17 signaling pathway. Our research strategy provided a template for the drug development of TCM for the treatment of CI.

## 1. Introduction

Cerebral infarction (CI), also known as ischemic stroke, is very common in cerebrovascular diseases, and the number of patients with this disease accounts for about 70% of patients with acute cerebrovascular diseases, which indicates that reasonable treatment is important for patients with this disease. The disease is mainly attributed to local tissue ischemia and hypoxia caused by cerebral blood flow disorders, followed by brain tissue necrosis and, in severe cases, neurological impairment [1]. At the same time, CI has the characteristics of complex pathogenesis, high mortality as well as recurrence rate, and an effective treatment plan is urgently needed for its treatment in clinical practice. Thrombolytic therapy is a feasible treatment for CI, but this method has the problem of short half-life of drugs, which may cause body toxicity if the preparation is used in large amounts. Therefore, we wanted to find a new drug to replace thrombolytic therapy for CI.

With an in-depth understanding of traditional Chinese medicine, people focus on finding appropriate traditional Chinese medicine (TCM) for the treatment of CI. In TCM theory, CI belongs to the category of “stroke,” and the common symptom is blood stasis. Therefore, drugs promoting blood circulation to remove blood stasis can be used to treat CI [1].

*Salvia miltiorrhiza* Bunge (SMB), a traditional Chinese medicine with the effect of promoting blood circulation and removing blood stasis, is also used as a botanical drug for the treatment of diseases in many Asian countries in addition to its widespread use in China [2]. At the same time, modern pharmacological studies have shown that SMB can inhibit thrombosis by improving blood circulation [3].

Compound *Salvia miltiorrhiza* (CSM) injection prepared with SMB as the main raw material is a commonly used drug for promoting blood circulation and removing blood stasis, which has been authorized by the China Food and Drug Administration. CSM is available to treat CI [1]. However, due to the complex mechanism of TCM in the treatment of diseases, it is difficult for ordinary research methods to systematically elaborate on diverse mechanisms.

**Figure 1:**
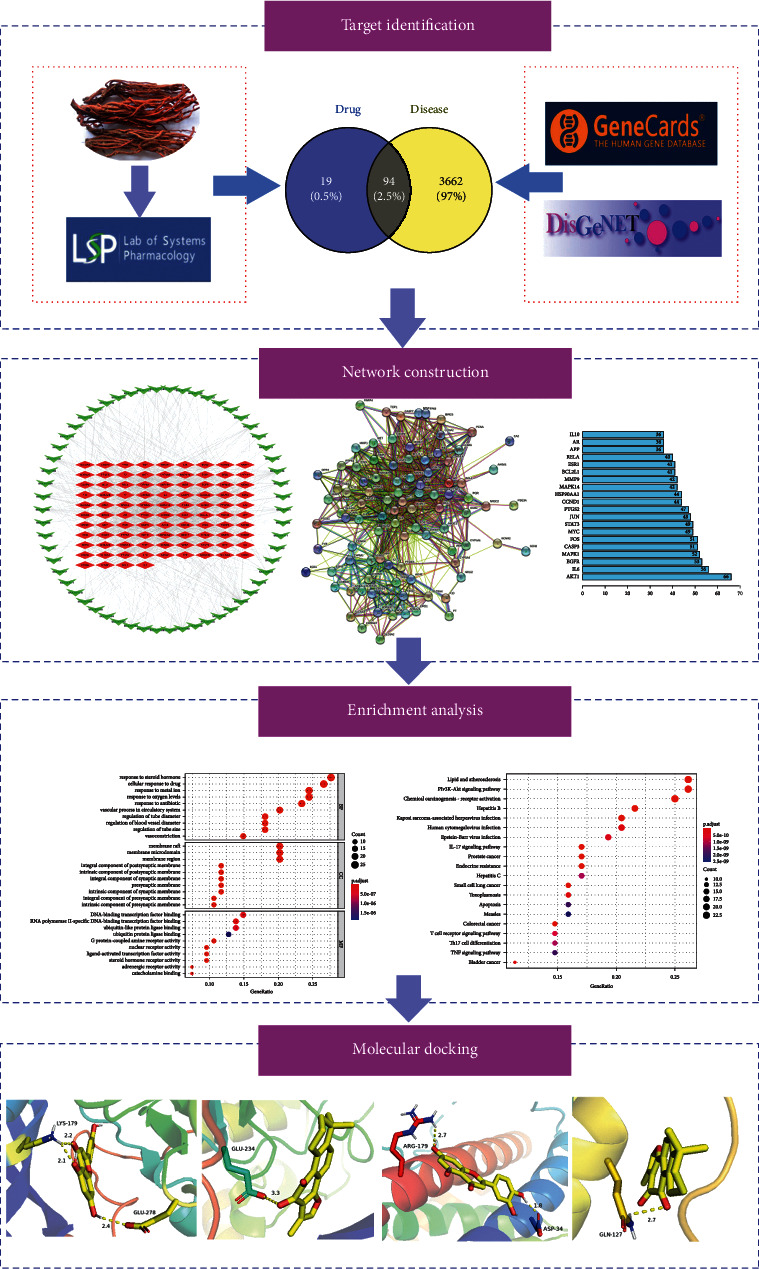
Molecular mechanism of *Salvia miltiorrhiza* Bunge in treating cerebral infarction.

Network pharmacology, as an emerging research tool, can be used to analyze the complex mechanisms of TCM in the treatment of diseases and reveal the route of drug action at the protein level [4]. Therefore, in this study, we selected network pharmacology to investigate the research means of SMB in the treatment of CI (Figure 1).

## 2. Materials and Methods

### 2.1. Screening Active Components of SMB

In general, oral bioavailability (OB) is an important measure of drug efficacy and drug-like properties (DL) represent the potential of compounds to become drugs [5]. We first collected all compounds contained in SMB in the TCM Systems Pharmacology (TCMSP) database (https://tcmspw.com/tcmspsearc-h.php), using “Radix Salviae” as the keyword. Then, we used (OB) ≥30% and (DL) ≥0.18 as the screening criteria to determine the potential active ingredients that may exist.

### 2.2. Predicting the Targets of the Compounds

We predicted possible targets associated with SMB compounds by searching the TCMSP database for “relevant targets” and normalized the target names by UniProt (https://www.uniprot.org/).

### 2.3. Screening of Disease Targets

CI related targets were searched in GeneCards and DisGeNET databases using the keyword “cerebral infarction.”

### 2.4. Construction of a Compound-Target (C-T) Network

Firstly, we constructed a network of drug compound targets interacting with disease targets by using Venny (https://bioinfogp.cnb.csic.es/tools/venny/index.html). Then, using Cytoscape V3.7.1 (https://www.cytoscape.org/) software, we performed a visual analysis of the C-T network.

### 2.5. Construction of a Protein-Protein Interaction (PPI) Network

Interaction networks between disease proteins were constructed by the Search Tool for the Retrieval of Interacting Genes/Proteins (STRING) (https://string-db.org/) [6].

### 2.6. GO (Gene Ontology) and KEGG Pathway Enrichment

Enrichment analysis of GO and KEGG for therapeutic proteins was performed by the “clusterProfiler” package in R (R Project for Statistical Computing, Vienna, Austria) [7].

### 2.7. Expression of Targets in Organs

The drugs refer to TCM and are used under the guidance of the TCM theory, while the medication law of SMB also follows the TCM theory. According to TCM theory, the occurrence of diseases is often due to the imbalance of the overall state of the body, and the lesions of a certain part or organ are often accompanied by abnormalities in other parts of the body. Therefore, during treatment, we need to implement the concept of combining global with local and multidimensional to gain insight into the development and changes of the disease [8]. We want to know whether SMB can modulate different organs to treat CI. Therefore, we obtained the expression of the top 20 proteins of the PPI network in different organs by BioGPS (https://biogps.org/).

### 2.8. Computational Validation of C–T Interactions

To verify the reliability of the previous prediction results, protein structures of AKT1 and IL-6 proteins with PDB codes 4EKL and 1ALU were obtained from the Research Collaboratory for Structural Bioinformatics Protein Data Bank (PDB) https://www.rcsb.org/), respectively. We performed in silico docking studies by AutoDock Vina software for the two predicted specific compounds and the two targets described above, respectively. We used the fraction of binding energy as a criterion to evaluate the possibility of binding of components to proteins. It is generally accepted that the smaller the binding energy score, the greater the possibility that components act on proteins [9].

## 3. Results

### 3.1. Screening of Active Components and Targets of SMB

In the TCMSP database, 202 possible chemical constituents of SMB were obtained. According to the screening parameters of OB ≥30% and DL ≥0.18, a total of 59 potential active ingredients corresponding to 113 drug targets were obtained by excluding components without corresponding targets (Table 1). The 113 potential drug targets are detailed in Supplementary Table S1.

### 3.2. Acquisition of Known Therapeutic Targets for CI

A total of 3,756 therapeutic targets of CI were obtained from GeneCards and DisGeNET after the removal of duplicated targets (Supplementary Table S2).

### 3.3. Analyses of the C-T Network

**Figure 2:**
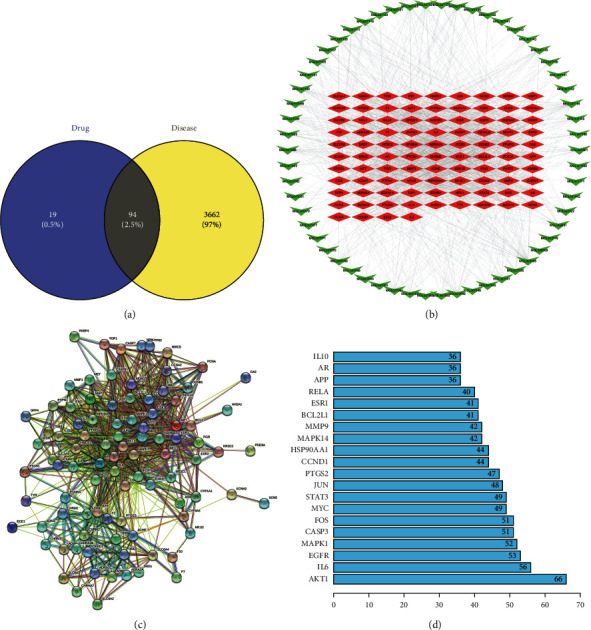
(a) Overlapping targets between diseases and drugs. (b) C-T network. (c) PPI network. (d) Bar plot of the PPI network.

There were 94 overlapping targets between 58 active component-related targets and disease targets in SMB (Figures 2(a) and 2(b)), indicating that these 94 proteins were target proteins for the treatment of CI by SMB. The active ingredients in SMB were indicated by green arrows; therapeutic targets were indicated by red squares.

Further analysis of the C-T network revealed that the node degrees of the components luteolin, tanshinone IIA, and dihydrotanshinone were 43, 35, and 26, respectively. These components have a high node degree, indicating that these components may be key to CI therapy.

Modern studies have shown that some of the components in SMB have a role in treating CI. For example, luteolin can activate the AMPK/mTOR signaling pathway and improve neurological impairment due to CI [56]. Tanshinone IIA can prevent CI development by inhibiting neuronal apoptosis and inflammatory responses [57]. This also justifies to some extent the predictions based on network pharmacology.

### 3.4. Analyses of the PPI Network

As shown in Figure 2(c), we constructed the regulatory relationship between the above 94 therapeutic targets into a PPI network for presentation (Supplementary Table S3). Then, the top 20 ranked proteins are selected according to the node degree (Figure 2(d)). It can be seen that the node degrees of AKT1, IL-6, and EGFR are 66, 56, and 53, respectively. They belong to the top three targets ranked by degree, indicating that they may play a key role in therapy and are the focus of subsequent studies.

### 3.5. Pathway-Enrichment Analyses Using GO and KEGG Databases

**Figure 3:**
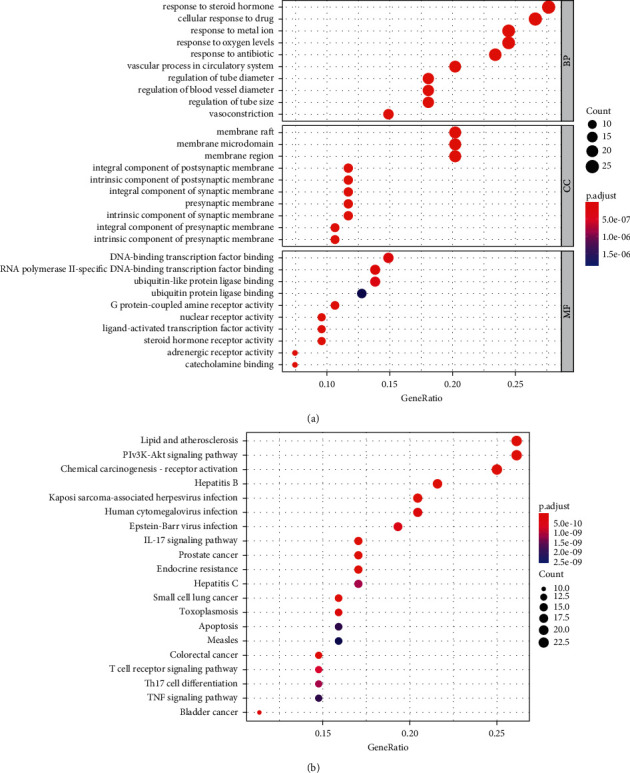
(a) GO process enrichment results. (b) KEGG pathway enrichment results.

We selected the top 10 biological processes (BP), cellular components (CC), and molecular functions (MF) of 94 therapeutic targets for analysis (Figure 3(a)). BP mainly includes response to steroid hormone (GO:0048545), vascular process in circulatory system (GO:0003018), and cellular response to drug (GO:0035690). CC mainly includes membrane raft (GO:0045121), membrane microdomain (GO:0098857), and membrane region (GO:0098589). MF mainly includes G protein-coupled amine receptor activity (GO:0008227), adrenergic receptor activity (GO:0004935), and nuclear receptor activity (GO:0004879). The details of the GO process enrichment results are shown in Supplementary Table S4.

Analysis of the KEGG pathway enrichment, results revealed that SMB may treat CI by regulating 138 pathways. We selected the top 20 pathways for analysis (Figure 3(b)). The results showed that SMB treatment of CI mainly involved lipid and atherosclerosis, chemical carcinogenesis-receptor activation, IL-17 signaling pathway, and other pathways (Supplementary Table S5).

### 3.6. Expression of Targets in Different Organs

**Figure 4:**
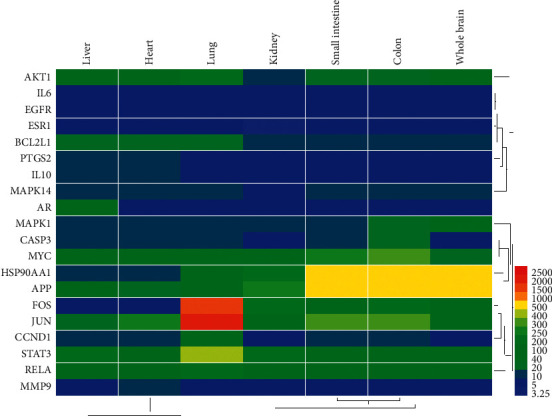
Heatmap of target expression in different organs. The *x*-axis indicates the organ name. The *y*-axis indicates the target name; from left to right, the liver, heart, kidney, lung, small intestine, and whole brain.

According to TCM theory, when lesions occur in local organs or tissues, they often cause reactions in other parts of the body. Therefore, we selected the top 20 proteins of the PPI network to observe their expression in different organs (Figure 4).

We can see that most proteins are also highly expressed in the lungs, in addition to higher expression in the brain. This suggests that it may be possible that SMB may also be linked to physiological processes in the lungs when treating CI through these targets. Studies have also shown that lobectomy may cause serious cardioembolic cerebral infarction complications [58]. This also illustrates to some extent that SMB may play a therapeutic role by regulating the organs associated with CI and allowing them to reach homeostasis.

### 3.7. Computational Assessment of Selected C-T Interactions

We selected AKT1, IL-6 and luteolin, and tanshinone IIA, respectively, for docking activity validation by in silico techniques. When the binding energy of protein and active ingredient is <0, it indicates that the active ingredient has the possibility of acting on protein. The lower the binding energy, the more reliable the possibility of action [9].

**Figure 5:**
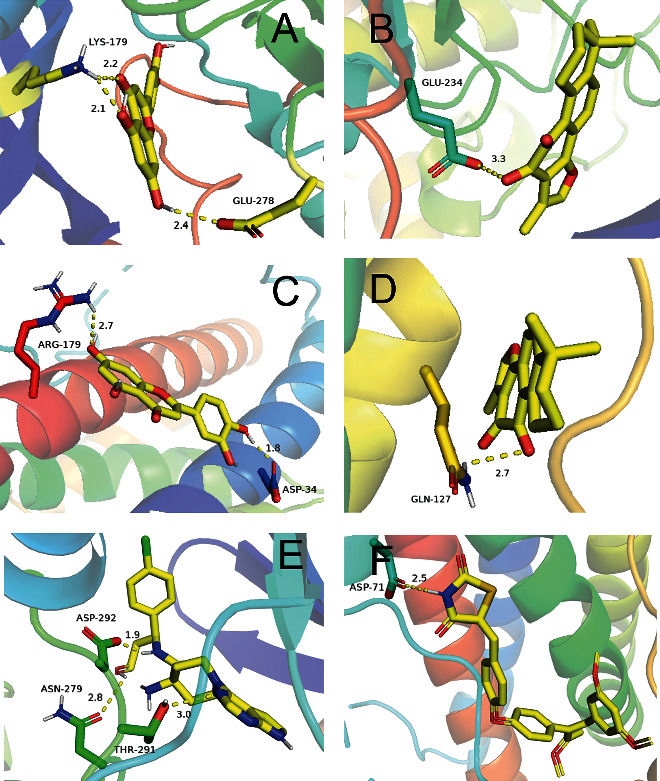
Binding studies of selected compound-target interactions. (a) Luteolin with AKT1. (b) Luteolin with IL-6. (c) Tanshinone IIA with AKT1. (d) Tanshinone IIA with IL-6. (e) AKT1 and small molecule inhibitors. (f) IL-6 and small molecule inhibitors.

First, we selected luteolin and AKT1 and IL-6, respectively, for binding validation. The calculated score between luteolin and AKT1 was −8.5 kcal/mol. The calculated score of luteolin with IL-6 was −7.1 kcal/mol, and hydrogen bonds were formed between luteolin and multiple amino acid residues of AKT1 and IL-6, respectively (Figures 5(a) and 5(b)), indicating that luteolin may act on the above two proteins.

Next, tanshinone IIA was docked with AKT1 and IL-6, respectively. The docking scores were −9.1 and −7.3 kcal/mol, respectively. Tanshinone IIA can also form hydrogen bonds with multiple amino acid residues of AKT1 and IL-6, respectively (Figures 5(c) and 5(d)). The validation results illustrate that tanshinone IIA had a good possibility of binding to two CI targets.

Finally, we performed activity validation of AKT1 and IL-6 proteins with their small molecule inhibitors, respectively, for judging the reliability of predicting active components. The calculated scores for the two were -9.1 and -6.8 kcal/mol, respectively. At the same time, both small molecule inhibitors and predicted active components bind to the same active pocket of the corresponding protein, except that the amino acid residues that form hydrogen bonds are different (Figures 5(e) and 5(f)), which was determined by the different chemical properties between the components. Overall, the above results suggest some plausibility of the previously predicted active ingredient.

## 4. Discussion

It is reported that patients who die due to CI account for about 40% of patients with cardiovascular disease each year in China, with the number of people affected being about 1.2 million [59]. For the health of patients, the development of an effective treatment for CI is currently the primary problem. CI is generally a brain function impairment caused by blood circulation dysfunction. According to TCM theory, CI is caused by blood stasis and venation obstruction, which is consistent with the clinical treatment symptoms of SMB, indicating that SMB may be a potential drug for the treatment of CI. Therefore, we hope to explore the scientificity and mechanism of action of SMB in the treatment of CI using network pharmacology.

Luteolin, tanshinone IIA, miltirone, dihydrotanshinlactone, cryptotanshinone, and isocryptotanshinone and other components may be the main material basis for CI treatment. Modern studies have shown that luteolin can regulate oxidative stress and related proteins on apoptosis pathways (such as TLR4, TLR5, p38 MAPK, etc.), thereby alleviating CI [60]. Tanshinone IIA can treat CI by various mechanisms such as promoting cerebral blood circulation and inhibiting inflammatory processes [61]. Supercritical CO_2_ extract of SMB with a cryptotanshinone content of 4.55% can alleviate cerebral ischemic injury by reducing thrombosis and platelet aggregation [62]. Miltirone is considered as a potential drug for the treatment of CI through antiplatelet drugs [63]. Based on the above evidence, it is scientific that we use network pharmacology to screen the active ingredients of SMB for the treatment of CI. Luteolin, tanshinone IIA, miltirone and other components may play a key role in the treatment of CI.

Proteins such as AKT1, IL-6, EGFR, and MAPK1 occupy an important position in PPI networks. AKT protein plays a key role in the occurrence and development of CI, and AKT protein phosphorylation can play a protective role when brain tissue is ischemic. At the same time, AKT can also regulate the biological activity of many nerve cells [63]. Inflammatory factors can not only indicate the physiological status of CI patients but also regulate the progression of CI itself. In general, at the onset of CI, the cerebral blood flow circulation is interrupted, causing the release of a large number of inflammatory factors including IL-6 and recruiting a variety of immune cells (such as macrophages, T lymphocytes, and natural killer cells) to accumulate, resulting in an excessive immune response [64]. Therefore, reasonable control of the occurrence of inflammatory response can effectively reduce the harm of CI to the body. Neurogenesis is an important mechanism for the treatment of CI, and EGFR can activate and regulate neurogenesis during CI development and is a key protein in CI treatment [65]. In addition, the phosphorylation level of MAPK1 is also closely related to neuronal survival [66].

GO enrichment results indicate that CI treatment mainly regulates processes such as response to steroid hormone, membrane raft, and G protein-coupled amine receptor activity. The signaling pathway enrichment results suggest that pathways such as lipid and atherosclerosis, chemical carcinogenesis-receptor activation, and IL-17 signaling pathway are of higher importance in CI therapy.

## 5. Conclusions

SMB is a commonly used drug in TCM clinical practice and has great application prospects. We predicted the mechanism of action of SMB in the treatment of CI by network pharmacology and validated some of our prediction results using in silico docking techniques. Of course, more evidence is needed to show the effectiveness of SMB in the treatment of CI, which will also be the focus of our later study. Our study provides a reference for TCM treatment of CI.

## Figures and Tables

**Table 1:** A total of fifty-nine ingredients were selected as the details of the active ingredients of SMB in this study.

No.	Mol ID	Molecule name	OB	DL	Reference
1	MOL001601	Trijuganone B	38.74	0.35	[10]
2	MOL001659	Poriferasterol	43.82	0.75	[11]
3	MOL001771	Poriferast-5-en-3beta-ol	36.91	0.75	[12]
4	MOL001942	Isoimperatorin	45.46	0.22	[13]
5	MOL002222	Sugiol	36.11	0.27	[14]
6	MOL002651	Dihydrotanshinone IIA	43.76	0.40	[15]
7	MOL002776	Baicalin	40.12	0.75	[16]
8	MOL000569	Digallate	61.84	0.25	
9	MOL000006	Luteolin	36.16	0.24	[17]
10	MOL007036	5,6-Dihydroxy-7-isopropyl-1, 1-dimethyl-2,3-dihydrophenanthren-4-one	33.76	0.28	[18]
11	MOL007041	2-Isopropyl-8-methylphenanthrene-3,4-dione	40.86	0.22	[19]
12	MOL007045	3*α*-Hydroxytanshinone IIA	44.92	0.44	[20]
13	MOL007048	(E)-3-[2-(3,4-Dihydroxyphenyl)-7-hydroxy-benzofuran-4-yl]acrylic acid	48.24	0.31	[21]
14	MOL007049	4-Methylenemiltirone	34.34	0.22	[22]
15	MOL007050	2-(4-Hydroxy-3-methoxyphenyl)-5-(3-hydroxypropyl)-7-methoxy-3-benzofurancarboxaldehyde	62.78	0.39	[23]
16	MOL007058	Formyltanshinone	73.44	0.41	
17	MOL007059	3-Beta-hydroxymethyllenetanshiquinone	32.16	0.40	
18	MOL007061	Methylenetanshinquinone	37.07	0.36	[24]
19	MOL007063	Przewalskin A	37.10	0.64	[25]
20	MOL007064	Przewalskin B	110.32	0.43	[26]
21	MOL007068	Przewaquinone B	62.24	0.41	[27]
22	MOL007069	Przewaquinone C	55.74	0.40	[28]
23	MOL007070	(6S, 7R)-6,7-Dihydroxy-1,6-dimethyl-8,9-dihydro-7h-naphtho[8, 7-g]benzofuran-10,11-dione	41.31	0.45	
24	MOL007071	Przewaquinone F	40.30	0.45	[28]
25	MOL007077	Sclareol	43.67	0.20	[29]
26	MOL007079	Tanshinaldehyde	52.47	0.45	[30]
27	MOL007081	Danshenol B	57.95	0.55	[31]
28	MOL007082	Danshenol A	56.96	0.52	[32]
29	MOL007085	Salvilenone	30.38	0.37	[33]
30	MOL007088	Cryptotanshinone	52.34	0.39	[34]
31	MOL007093	Dan-shexinkum D	38.88	0.55	[35]
32	MOL007094	Danshenspiroketallactone	50.43	0.30	[36]
33	MOL007098	Deoxyneocryptotanshinone	49.40	0.28	[37]
34	MOL007100	Dihydrotanshinlactone	38.68	0.32	
35	MOL007101	Dihydrotanshinone I	45.04	0.36	[38]
36	MOL007105	Epidanshenspiroketallactone	68.27	0.30	[36]
37	MOL007107	Ferruginol	36.06	0.24	[39]
38	MOL007108	Isocryptotanshi-none	54.98	0.39	[38]
39	MOL007111	Isotanshinone II	49.91	0.39	[40]
40	MOL007115	Manool	45.04	0.20	[41]
41	MOL007119	Miltionone I	49.68	0.32	[42]
42	MOL007120	Miltionone II	71.02	0.43	[43]
43	MOL007121	Miltipolone	36.55	0.36	[44]
44	MOL007122	Miltirone	38.75	0.25	[45]
45	MOL007124	Neocryptotanshinone II	39.46	0.23	[46]
46	MOL007125	Neocryptotanshinone	52.48	0.32	[47]
47	MOL007127	1-Methyl-8,9-dihydro-7h-naphtho[5,6-g]benzofuran-6,10,11-trione	34.72	0.36	[48]
48	MOL007130	Prolithospermic acid	64.37	0.31	[49]
49	MOL007132	(2R)-3-(3,4-Dihydroxyphenyl)-2-[(Z)-3-(3,4-dihydroxyphenyl)acryloyl]oxy-propionic acid	109.38	0.35	
50	MOL007141	Salvianolic acid G	45.56	0.60	[50]
51	MOL007142	Salvianolic acid J	43.37	0.72	[51]
52	MOL007143	Salvilenone I	32.43	0.22	[33]
53	MOL007145	Salviolone	31.72	0.23	[52]
54	MOL007150	(6S)-6-Hydroxy-1-methyl-6-methylol-8,9-dihydro-7h-naphtho[8,7-g]benzofuran-10,11-quinone	75.38	0.45	[51]
55	MOL007151	Tanshindiol B	42.66	0.45	[52]
56	MOL007152	Przewaquinone E	42.85	0.45	[28]
57	MOL007154	Tanshinone IIA	49.88	0.39	[53]
58	MOL007155	(6S)-6-(Hydroxymethyl)-1,6-dimethyl-8,9-dihydro-7h-naphtho[8,7-g]benzofuran-10,11-dione	65.25	0.44	[54]
59	MOL007156	Tanshinone VI	45.63	0.29	[55]

## Data Availability

The data used to support the findings of this study are available from the corresponding author upon request. The authors obtained the composition of rhubarb and its potential target from the TCMSP database (https://tcmsp-e.com/). They also obtained the potential target of cancer according to the DisGeNET database (https://www.disgenet.org/search) and GeneCards database (https://www.genecards.org) and, subsequently, PPI analysis (STRING database, https://string-db.org/), 343 KEGG pathways analysis, and GO biological processes (clusterProfiler package).

## Additional Points

Traditional Chinese medicine (TCM) is a complicated system containing hundreds of constituents responsible for their therapeutic effects. As a brand-new area of pharmacology, network pharmacology provides new approaches of drug discovery for complex diseases and offers new methods for elucidating the multiple action mechanisms of drugs. In TCM theory, cerebral infarction (CI) belongs to the category of “stroke” and the common symptom is blood stasis, which can be treated by blood-activating and stasis-dissipating drugs. This is consistent with the therapeutic symptoms of *Salvia miltiorrhiza* Bunge (SMB), but its specific active components and mechanism of action are not clear. In this study, network pharmacology approaches were used to investigate the possible mechanism underlying the effectiveness of SMB in the treatment of CI. Firstly, our work was to demonstrate the efficiency of SMB in the treatment of CI. Our findings did not only provide scientific proofs for SMB in the treatment of CI but also indicate the feasibility of studying the mechanism of action of TCM in treating diseases based on network pharmacology. Our work provides a direction for elucidating the scientific connotation of traditional Chinese medicine in the treatment of diseases and drug discovery.

## Supplementary Materials

Supplementary Materials Table S1: details of active compounds and targets. Table S2: known therapeutic targets in cerebral infarction. Table S3: the details of the PPI network. Table S4: the details of the GO analysis. Table S5: the KEGG pathway enrichment analysis.
