# Supplementary material for: Molecular Mechanism of Salvia miltiorrhiza Bunge in Treating Cerebral Infarction
Source: Evid Based Complement Alternat Med. 2022 Mar 29;2022:5992394. doi: 10.1155/2022/5992394 (PMC8983215; doi:10.1155/2022/5992394)
Supplement: Supplementary Materials — Table S1: details of active compounds and targets. Table S2: known therapeutic targets in cerebral infarction. Table S3: the details of the PPI network. Table S4: the details of the GO analysis. Table S5: the KEGG pathway enrichment analysis. [file 5992394.f1.zip › 5992394.f1/Table S1-Details of active compounds and targets (2).doc]

**Table S1: Details of active compounds and targets**

| **Mol ID** | **Target** |
| --- | --- |
| MOL001601; MOL001601; MOL001601; MOL001601; MOL001601; MOL001601; MOL001601; MOL001601; MOL001601; MOL001601; MOL001601; MOL001601; MOL001601; MOL001601; MOL001601; MOL001601; MOL001601; MOL001601; MOL001601; MOL001601; MOL001601; MOL001601; MOL001601; MOL001601; MOL001601; MOL001659; MOL001659; MOL001771; MOL001771; MOL001942; MOL002222; MOL002222; MOL002222; MOL002222; MOL002222; MOL002222; MOL002222; MOL002222; MOL002222; MOL002222; MOL002222; MOL002222; MOL002222; MOL002222; MOL002222; MOL002222; MOL002222; MOL002651; MOL002651; MOL002651; MOL002651; MOL002651; MOL002651; MOL002651; MOL002651; MOL002651; MOL002651; MOL002651; MOL002651; MOL002651; MOL002651; MOL002651; MOL002651; MOL002651; MOL002651; MOL002651; MOL002776; MOL002776; MOL000569; MOL000569; MOL000569; MOL000006; MOL000006; MOL000006; MOL000006; MOL000006; MOL000006; MOL000006; MOL000006; MOL000006; MOL000006; MOL000006; MOL000006; MOL000006; MOL000006; MOL000006; MOL000006; MOL000006; MOL000006; MOL000006; MOL000006; MOL000006; MOL000006; MOL000006; MOL000006; MOL000006; MOL000006; MOL000006; MOL000006; MOL000006; MOL000006; MOL000006; MOL000006; MOL000006; MOL000006; MOL000006; MOL000006; MOL000006; MOL000006; MOL000006; MOL000006; MOL000006; MOL000006; MOL000006; MOL000006; MOL000006; MOL000006; MOL000006; MOL000006; MOL000006; MOL000006; MOL000006; MOL007036; MOL007036; MOL007036; MOL007036; MOL007036; MOL007036; MOL007036; MOL007036; MOL007036; MOL007036; MOL007036; MOL007036; MOL007036; MOL007036; MOL007036; MOL007041; MOL007041; MOL007041; MOL007041; MOL007041; MOL007041; MOL007041; MOL007041; MOL007041; MOL007041; MOL007041; MOL007041; MOL007041; MOL007041; MOL007041; MOL007041; MOL007041; MOL007041; MOL007041; MOL007041; MOL007041; MOL007041; MOL007041; MOL007041; MOL007045; MOL007045; MOL007045; MOL007045; MOL007045; MOL007045; MOL007045; MOL007045; MOL007045; MOL007045; MOL007045; MOL007045; MOL007045; MOL007048; MOL007048; MOL007048; MOL007049; MOL007049; MOL007049; MOL007049; MOL007049; MOL007049; MOL007049; MOL007049; MOL007049; MOL007049; MOL007049; MOL007049; MOL007049; MOL007049; MOL007049; MOL007049; MOL007049; MOL007049; MOL007049; MOL007049; MOL007049; MOL007049; MOL007049; MOL007049; MOL007049; MOL007049; MOL007049; MOL007049; MOL007049; MOL007049; MOL007050; MOL007050; MOL007050; MOL007050; MOL007050; MOL007050; MOL007050; MOL007050; MOL007050; MOL007050; MOL007058; MOL007058; MOL007058; MOL007058; MOL007058; MOL007058; MOL007058; MOL007059; MOL007059; MOL007059; MOL007059; MOL007059; MOL007059; MOL007059; MOL007059; MOL007059; MOL007059; MOL007059; MOL007059; MOL007059; MOL007059; MOL007059; MOL007059; MOL007061; MOL007061; MOL007061; MOL007061; MOL007061; MOL007061; MOL007061; MOL007061; MOL007061; MOL007061; MOL007061; MOL007061; MOL007061; MOL007061; MOL007061; MOL007061; MOL007061; MOL007061; MOL007061; MOL007061; MOL007061; MOL007063; MOL007063; MOL007064; MOL007064; MOL007064; MOL007064; MOL007064; MOL007064; MOL007068; MOL007068; MOL007068; MOL007068; MOL007068; MOL007068; MOL007068; MOL007069; MOL007069; MOL007069; MOL007069; MOL007069; MOL007069; MOL007069; MOL007069; MOL007069; MOL007069; MOL007069; MOL007069; MOL007069; MOL007069; MOL007069; MOL007069; MOL007069; MOL007069; MOL007069; MOL007069; MOL007070; MOL007070; MOL007070; MOL007070; MOL007070; MOL007070; MOL007070; MOL007070; MOL007071; MOL007071; MOL007071; MOL007071; MOL007071; MOL007077; MOL007079; MOL007079; MOL007079; MOL007079; MOL007079; MOL007079; MOL007079; MOL007079; MOL007079; MOL007079; MOL007079; MOL007079; MOL007081; MOL007081; MOL007081; MOL007081; MOL007081; MOL007081; MOL007081; MOL007081; MOL007082; MOL007082; MOL007082; MOL007082; MOL007082; MOL007082; MOL007082; MOL007085; MOL007085; MOL007085; MOL007085; MOL007085; MOL007085; MOL007088; MOL007088; MOL007088; MOL007088; MOL007088; MOL007088; MOL007088; MOL007088; MOL007088; MOL007088; MOL007088; MOL007088; MOL007088; MOL007088; MOL007088; MOL007088; MOL007088; MOL007088; MOL007088; MOL007088; MOL007088; MOL007088; MOL007088; MOL007088; MOL007088; MOL007088; MOL007088; MOL007088; MOL007088; MOL007093; MOL007093; MOL007093; MOL007093; MOL007093; MOL007093; MOL007093; MOL007093; MOL007093; MOL007093; MOL007093; MOL007093; MOL007093; MOL007093; MOL007093; MOL007093; MOL007093; MOL007093; MOL007093; MOL007093; MOL007093; MOL007093; MOL007093; MOL007093; MOL007093; MOL007094; MOL007094; MOL007094; MOL007094; MOL007094; MOL007094; MOL007094; MOL007094; MOL007094; MOL007094; MOL007094; MOL007094; MOL007094; MOL007094; MOL007094; MOL007094; MOL007094; MOL007094; MOL007094; MOL007094; MOL007094; MOL007094; MOL007094; MOL007094; MOL007098; MOL007098; MOL007098; MOL007098; MOL007098; MOL007098; MOL007098; MOL007098; MOL007098; MOL007098; MOL007098; MOL007098; MOL007098; MOL007098; MOL007098; MOL007098; MOL007098; MOL007098; MOL007098; MOL007098; MOL007098; MOL007098; MOL007098; MOL007098; MOL007100; MOL007100; MOL007100; MOL007100; MOL007100; MOL007100; MOL007100; MOL007100; MOL007100; MOL007100; MOL007100; MOL007100; MOL007100; MOL007100; MOL007100; MOL007100; MOL007100; MOL007100; MOL007100; MOL007100; MOL007100; MOL007100; MOL007100; MOL007100; MOL007100; MOL007100; MOL007100; MOL007100; MOL007100; MOL007100; MOL007101; MOL007101; MOL007101; MOL007101; MOL007101; MOL007101; MOL007101; MOL007101; MOL007101; MOL007101; MOL007101; MOL007101; MOL007105; MOL007105; MOL007105; MOL007105; MOL007105; MOL007105; MOL007105; MOL007105; MOL007105; MOL007105; MOL007105; MOL007105; MOL007105; MOL007105; MOL007105; MOL007105; MOL007105; MOL007105; MOL007105; MOL007105; MOL007105; MOL007107; MOL007107; MOL007107; MOL007107; MOL007107; MOL007107; MOL007107; MOL007107; MOL007107; MOL007107; MOL007107; MOL007107; MOL007108; MOL007108; MOL007108; MOL007108; MOL007108; MOL007108; MOL007108; MOL007108; MOL007108; MOL007108; MOL007108; MOL007108; MOL007108; MOL007108; MOL007108; MOL007108; MOL007108; MOL007108; MOL007108; MOL007108; MOL007108; MOL007108; MOL007108; MOL007108; MOL007108; MOL007108; MOL007108; MOL007108; MOL007111; MOL007111; MOL007111; MOL007111; MOL007111; MOL007111; MOL007111; MOL007111; MOL007111; MOL007111; MOL007111; MOL007111; MOL007111; MOL007111; MOL007111; MOL007111; MOL007111; MOL007111; MOL007111; MOL007111; MOL007111; MOL007111; MOL007111; MOL007115; MOL007119; MOL007119; MOL007119; MOL007119; MOL007119; MOL007119; MOL007119; MOL007119; MOL007119; MOL007119; MOL007119; MOL007119; MOL007119; MOL007119; MOL007119; MOL007119; MOL007119; MOL007119; MOL007119; MOL007119; MOL007119; MOL007119; MOL007119; MOL007120; MOL007120; MOL007120; MOL007120; MOL007120; MOL007120; MOL007120; MOL007120; MOL007121; MOL007121; MOL007122; MOL007122; MOL007122; MOL007122; MOL007122; MOL007122; MOL007122; MOL007122; MOL007122; MOL007122; MOL007122; MOL007122; MOL007122; MOL007122; MOL007122; MOL007122; MOL007122; MOL007122; MOL007122; MOL007122; MOL007122; MOL007122; MOL007122; MOL007122; MOL007122; MOL007124; MOL007124; MOL007124; MOL007124; MOL007124; MOL007124; MOL007124; MOL007124; MOL007124; MOL007124; MOL007124; MOL007124; MOL007124; MOL007124; MOL007124; MOL007124; MOL007124; MOL007124; MOL007124; MOL007124; MOL007124; MOL007124; MOL007124; MOL007124; MOL007124; MOL007125; MOL007125; MOL007125; MOL007125; MOL007125; MOL007125; MOL007125; MOL007125; MOL007125; MOL007125; MOL007125; MOL007125; MOL007125; MOL007125; MOL007125; MOL007127; MOL007127; MOL007127; MOL007127; MOL007127; MOL007127; MOL007127; MOL007127; MOL007127; MOL007127; MOL007127; MOL007127; MOL007127; MOL007127; MOL007127; MOL007127; MOL007127; MOL007130; MOL007130; MOL007130; MOL007130; MOL007130; MOL007130; MOL007130; MOL007130; MOL007130; MOL007132; MOL007132; MOL007132; MOL007132; MOL007132; MOL007132; MOL007132; MOL007132; MOL007141; MOL007142; MOL007142; MOL007142; MOL007143; MOL007143; MOL007143; MOL007143; MOL007143; MOL007143; MOL007143; MOL007143; MOL007145; MOL007145; MOL007145; MOL007145; MOL007145; MOL007145; MOL007145; MOL007145; MOL007145; MOL007145; MOL007145; MOL007145; MOL007145; MOL007145; MOL007145; MOL007145; MOL007145; MOL007145; MOL007145; MOL007145; MOL007145; MOL007145; MOL007145; MOL007145; MOL007145; MOL007145; MOL007145; MOL007145; MOL007150; MOL007150; MOL007150; MOL007150; MOL007150; MOL007150; MOL007150; MOL007150; MOL007151; MOL007151; MOL007151; MOL007151; MOL007151; MOL007151; MOL007151; MOL007152; MOL007152; MOL007152; MOL007152; MOL007152; MOL007152; MOL007152; MOL007154; MOL007154; MOL007154; MOL007154; MOL007154; MOL007154; MOL007154; MOL007154; MOL007154; MOL007154; MOL007154; MOL007154; MOL007154; MOL007154; MOL007154; MOL007154; MOL007154; MOL007154; MOL007154; MOL007154; MOL007154; MOL007154; MOL007154; MOL007154; MOL007154; MOL007154; MOL007154; MOL007154; MOL007154; MOL007154; MOL007154; MOL007154; MOL007154; MOL007154; MOL007154; MOL007154; MOL007154; MOL007154; MOL007154; MOL007154; MOL007155; MOL007155; MOL007155; MOL007155; MOL007155; MOL007155; MOL007155; MOL007155; MOL007155; MOL007155; MOL007155; MOL007155; MOL007155; MOL007156; MOL007156; MOL007156; MOL007156; MOL007156; MOL007156; MOL007156; MOL007156; MOL007156 MOL007156 | PTGS1; CHRM3; CHRM1; SCN5A; CHRM5; PTGS2; CA2; CHRM4; RXRA; OPRD1; PDE3A; ADRA1A; CHRM2; ADRA1B; SLC6A3; ADRB2; ADRA1D; TOP2A; OPRM1; HSP90AA1; CHRNA7; NCOA2; NCOA1; DRD1; SLC6A4; PGR; NR3C2; PGR; NCOA2; PTGS2; CHRM3; CHRM1; SCN5A; CHRM5; PTGS2; CA2; CHRM4; OPRD1; ACHE; ADRA1A; CHRM2; ADRA1B; ADRB2; ADRA1D; DRD2; OPRM1; CHRNA7; DRD1; CHRM3; F2; CHRM1; ESR1; AR; SCN5A; PPARG; CHRM5; PTGS2; CHRM4; OPRD1; ACHE; ADRA1A; ADRB2; OPRM1; DPP4; CHRNA7; NCOA1; F10; PTPN1; PTGS2; HSP90AA1; AKR1B1; PTGS1; AR; PTGS2; HSP90AA1; PRSS1; NCOA2; PRKACA; DPP4; RELA; EGFR; AKT1; CCND1; BCL2L1; CDKN1A; CASP9; MMP2; MMP9; MAPK1; IL10; RB1; TNFSF15; JUN; IL6; CASP3; TP63; NFKBIA; TOP1; MDM2; APP; MMP1; PCNA; ERBB2; PPARG; HMOX1; CASP7; ICAM1; MCL1; BIRC5; IL2; CCNB1; TYR; IFNG; IL4; TOP2A; SLC2A4; INSR; CD40LG; PTGES; NUF2; ADCY2; MET; PTGS1; CHRM3; CHRM1; SCN5A; PTGS2; CA2; RXRA; ACHE; ADRA1A; ADRA1B; ADRB2; TOP2A; OPRM1; NCOA2; NCOA1; PTGS1; DRD1; CHRM3; CHRM1; ESR1; AR; SCN5A; PPARG; CHRM5; PTGS2; CHRM4; RXRA; ADRA1A; CHRM2; ADRA1B; SLC6A3; ADRB2; ADRA1D; SLC6A4; OPRM1; CHRNA7; PRKACA; CCNA2; NCOA2; F2; CHRM1; SCN5A; CHRM5; PTGS2; OPRD1; ACHE; ADRB2; OPRM1; DPP4; CHRNA7; PRSS1; NCOA1; PTGS2; PTPN1; HSP90AA1; PTGS1; DRD1; CHRM3; CHRM1; ESR1; AR; SCN5A; PPARG; CHRM5; PTGS2; ADRA2A; CA2; ADRA2C; CHRM4; RXRA; OPRD1; ADRA1A; CHRM2; ADRA1B; SLC6A3; ADRB2; ADRA1D; TOP2A; SLC6A4; DRD2; OPRM1; CHRNA7; PRKACA; NCOA2; NCOA1; NOS2; F2; ESR1; AR; PPARG; ESR2; MAPK14; GSK3B; HSP90AA1; CCNA2; F2; AR; PTGS2; RXRA; DPP4; PRKACA; NCOA1; DRD1; F2; CHRM1; PTGS2; CA2; RXRA; OPRD1; ACHE; ADRA1A; ADRB2; OPRM1; DPP4; HSP90AA1; CHRNA7; PRSS1; NCOA1; DRD1; CHRM3; F2; CHRM1; SCN5A; CHRM5; PTGS2; CA2; RXRA; OPRD1; ACHE; ADRA1A; CHRM2; ADRB2; SLC6A4; OPRM1; DPP4; HSP90AA1; CHRNA7; PRSS1; NCOA1; NR3C2; NR3C1; PTGS2; PGR; NR3C2; NR3C1; NCOA2; NCOA1; F2; PTGS2; RXRA; DPP4; HSP90AA1; PRSS1; NCOA1; PTGS1; DRD1; CHRM3; F2; CHRM1; SCN5A; CHRM5; PTGS2; CA2; CHRM4; OPRD1; ACHE; ADRA1A; CHRM2; ADRB2; OPRM1; DPP4; HSP90AA1; CHRNA7; NCOA1; F2; PTGS2; CA2; ACHE; DPP4; HSP90AA1; PRSS1; NCOA1; F2; PTGS2; DPP4; PRSS1; NCOA1; PTGS2; DRD1; F2; CHRM1; PTGS2; OPRD1; ACHE; ADRB2; OPRM1; DPP4; CHRNA7; PRSS1; NCOA1; PTGS2; CA2; PGR; TOP2A; OPRM1; NR3C1; HSP90AA1; NCOA1; PTGS1; KCNH2; SCN5A; F10; PTGS2; RXRA; NCOA1; PTGS1; ESR1; AR; CHRM5; PTGS2; ESR2; PTGS1; DRD1; CHRM3; CHRM1; SCN5A; CHRM5; PTGS2; CA2; CHRM4; OPRD1; ADRA1A; CHRM2; ADRA1B; ADRB2; ADRA1D; TOP2A; OPRM1; CHRNA7; NCOA2; NCOA1; PGR; RELA; STAT3; CCND1; BCL2L1; TNFSF15; APP; EDN3; BIRC5; NOS2; PTGS1; F2; KCNH2; CHRM1; ESR1; AR; SCN5A; PPARG; F10; PTGS2; CA2; RXRA; ACHE; ADRA1B; ADRB2; TOP2A; ESR2; DPP4; GSK3B; CHEK1; PRSS1; CCNA2; NCOA2; NCOA1; PTGS1; DRD1; CHRM3; F2; CHRM1; ESR1; SCN5A; CHRM5; PTGS2; CA2; CHRM4; RXRA; ACHE; ADRA1A; CHRM2; ADRA1B; ADRB2; ADRA1D; CHRNA2; SLC6A4; OPRM1; DPP4; HSP90AA1; CHRNA7; PTGS1; DRD1; CHRM3; CHRM1; ESR1; AR; SCN5A; CHRM5; PTGS2; CA2; CHRM4; RXRA; OPRD1; ADRA1A; CHRM2; ADRA1B; ADRB2; ADRA1D; TOP2A; OPRM1; GSK3B; CHRNA7; NCOA2; NCOA1; NOS2; PTGS1; DRD1; CHRM3; F2; CHRM1; ESR1; AR; SCN5A; PPARG; CHRM5; PTGS2; CA2; RXRA; ACHE; PDE3A; ADRA1A; ADRA1B; PTPN1; SLC6A3; ADRB2; ADRA1D; SLC6A4; OPRM1; DPP4; GSK3B; CHRNA7; PRKACA; PRSS1; CCNA2; PTGS1; SCN5A; PTGS2; RXRA; ADRA1A; ADRA1B; ADRB2; HSP90AA1; CHRNA7; PRKACA; NCOA2; NCOA1; PTGS1; DRD1; CHRM3; CHRM1; ESR1; SCN5A; CHRM5; PTGS2; CHRM4; RXRA; OPRD1; PDE3A; ADRA1A; CHRM2; ADRA1B; ADRB2; ADRA1D; SLC6A4; OPRM1; HSP90AA1; CHRNA7; CHRM3; F2; CHRM1; SCN5A; CA2; ACHE; ADRA1A; CHRM2; ADRA1B; ADRB2; ADRA1D; OPRM1; NOS2; PTGS1; DRD1; CHRM3; CHRM1; ESR1; AR; SCN5A; F10; CHRM5; PTGS2; CA2; CHRM4; RXRA; OPRD1; ACHE; ADRA1A; CHRM2; ADRA1B; ADRB2; ADRA1D; TOP2A; DRD2; OPRM1; CHRNA7; PRSS1; NCOA2; NCOA1; NOS2; DRD1; CHRM3; F2; CHRM1; ESR1; AR; SCN5A; CHRM5; PTGS2; RXRA; OPRD1; ACHE; ADRA1A; CHRM2; ADRB2; OPRM1; ESR2; DPP4; GSK3B; CHRNA7; CHEK1; CCNA2; NCOA2; PTGS1; CHRM3; CHRM1; ESR1; AR; SCN5A; F10; PTGS2; CA2; RXRA; OPRD1; ADRA1A; CHRM2; ADRA1B; ADRB2; TOP2A; OPRM1; NR3C1; GSK3B; CHRNA7; CCNA2; NCOA2; NCOA1; F2; PTGS2; CA2; ACHE; PGR; NR3C1; NCOA2; NCOA1; ESR1; ACHE; PTGS1; DRD1; CHRM3; CHRM1; ESR1; AR; DRD5; SCN5A; CHRM5; PTGS2; CA2; ADRA2C; CHRM4; RXRA; OPRD1; ADRA1A; CHRM2; ADRA1B; SLC6A3; ADRB2; ADRA1D; TOP2A; OPRM1; CHRNA7; NCOA2; PTGS1; DRD1; CHRM3; CHRM1; ESR1; AR; SCN5A; PTGS2; CA2; CHRM4; RXRA; OPRD1; PDE3A; ADRA1A; CHRM2; ADRA1B; SLC6A3; ADRB2; ADRA1D; SLC6A4; OPRM1; GSK3B; HSP90AA1; CHRNA7; CCNA2; PTGS1; CHRM3; CHRM1; SCN5A; PPARG; PTGS2; CA2; ADRA1B; ADRB2; ADRA1D; TOP2A; OPRM1; CHRNA7; NCOA2; NCOA1; PTGS1; DRD1; CHRM3; F2; SCN5A; CHRM5; PTGS2; CA2; RXRA; ACHE; ADRA1A; ADRB2; OPRM1; DPP4; HSP90AA1; CHRNA7; NCOA1; NOS2; PTGS1; F2; ESR1; AR; PTGS2; PTPN1; HSP90AA1; PRSS1; F2; ESR1; AR; PPARG; PTGS2; DPP4; PRSS1; CCNA2; PTGS2; F7; PTPN1; PRSS1; PTGS2; RXRA; ACHE; PGR; PTPN1; NR3C1; NCOA2; NCOA1; PTGS1; DRD1; CHRM3; F2; CHRM1; DRD5; SCN5A; CHRM5; PTGS2; ADRA2A; CHRM4; OPRD1; ACHE; PDE3A; SLC6A2; ADRA1A; CHRM2; ADRA2B; ADRA1B; SLC6A3; ADRB2; CHRNA2; SLC6A4; DRD2; OPRM1; CHRNA7; GABRG3; GABRE; F2; PTGS2; CA2; ACHE; DPP4; HSP90AA1; PRSS1; NCOA1; F2; PTGS2; CA2; ACHE; DPP4; HSP90AA1; NCOA1; F2; PTGS2; CA2; ACHE; DPP4; HSP90AA1; NCOA1; DRD1; CHRM3; F2; CHRM1; SCN5A; CHRM5; PTGS2; CHRM4; OPRD1; ACHE; ADRA1A; CHRM2; ADRB2; OPRM1; DPP4; CHRNA7; NCOA1; RXRA; RELA; BCL2; FOS; CDKN1A; MMP9; JUN; AHSA1; CASP3; TP63; NFKBIA; FASN; EDNRA; EDN3; CYP3A4; MYC; CYP1A1; NR1I2; NPM1; ECE1; PARP4; CALCR; ITGB3; F2; CHRM1; SCN5A; PTGS2; OPRD1; ACHE; ADRA1A; ADRB2; OPRM1; DPP4; CHRNA7; PRSS1; NCOA1; PTGS1; ESR1; AR; SCN5A; PPARG; F10; PTGS2; HSP90AA1; NCOA2 NCOA1 |
