# Supplementary material for: Molecular Mechanism of Salvia miltiorrhiza Bunge in Treating Cerebral Infarction
Source: Evid Based Complement Alternat Med. 2022 Mar 29;2022:5992394. doi: 10.1155/2022/5992394 (PMC8983215; doi:10.1155/2022/5992394)
Supplement: Supplementary Materials — Table S1: details of active compounds and targets. Table S2: known therapeutic targets in cerebral infarction. Table S3: the details of the PPI network. Table S4: the details of the GO analysis. Table S5: the KEGG pathway enrichment analysis. [file 5992394.f1.zip › 5992394.f1/Table S2-Known Therapeutic Targets in Cerebral Infarction (2).doc]

**Table S2: Known Therapeutic Targets in Cerebral Infarction**

**Target**

NPY

PLAT

ALOX5AP

NOS3

F5

PRKCH

PLAU

VDR

ADH1B

HBA1

SERPINE1

MAP3K5

MAP2

ANXA1

IL18

SLC6A1

VIM

PTGDR

EPHX2

ADORA1

TSPAN33

TLR4

NFE2L2

PIK3CG

APOE

SIRT1

PIK3CD

GABPA

PIK3CB

BDNF

TNF

VEGFA

PIK3CA

C20orf181

IL1B

HMGB1

HIF1A

IL1A

IL1RN

ACE

MTHFR

NLRP3

HSPB3

MIR210

TGFB1

MAPK3

ACSM3

AQP4

HSPA4

HSPB1

HSPB2

CSF3

MIR155

LPL

ANGPT1

CRP

FGB

NGB

ADIPOQ

PTEN

FGF2

HGF

FGFR1

SOD1

KCNK2

AGT

MIR126

ABCA1

ALDH2

ALB

EPHB2

NGF

PLG

APOH

CAV1

CXCL12

PLA2G7

TXN

TNFSF4

SPP1

BSG

TRPM2

CBS

S100B

TBXA2R

REST

PINK1

TLR2

ATM

TRPC6

THBD

ROCK2

LCN2

EPO

NTN1

PON1

LDLR

DNM1L

F2R

MIR21

PROZ

MANF

IGF1

CCL2

ROS1

HP

SOD3

GDNF

NOX4

JAK2

ABCC8

RAC1

NTF3

CCL5

GFAP

HSPG2

GRN

GPER1

GRIK2

LGALS3

LEP

GRIN2A

KDR

MALAT1

KLK1

ANXA6

NR4A1

XIAP

RAB7B

SETD2

PPIG

STC1

SP1

CD40

SLC5A1

SLC2A1

RTN4R

SLC1A2

GAS5

REN

SEMA6A

MIR451A

SYP

MAP3K7

SPHK1

APLN

RIPK1

DENR

UBASH3B

XBP1

TRPV1

TRAF2

TP53

CLDN5

TFRC

PTGIS

MRS2

OLR1

NUCB2

NOTCH3

NFKB1

SERPINC1

COX2

MAP3K11

CX3CR1

MBL2

MIR223

MIR195

PAEP

CKLF

ACKR3

MAP2K7

MAPK8

MEG3

TRPM7

PLA2G4A

UTRN

PHB

PDE4D

PDE4A

TNFRSF12A

MIR130A

GPR17

ADM

GCG

CREB1

CIRBP

NAMPT

SIRT2

AGER

DLG4

DECR1

POLDIP2

LILRB1

FOXO3

CBSL

CYBB

CPB2

AGTR1

DAPK2

AIF1

CRMP1

MTOR

G6PD

CST3

FABP1

GLP1R

MTCO2P12

CDKN2B-AS1

FABP2

CSF2

CTNNB1

CTSS

COL3A1

CX3CL1

MAP4K1

CNR1

NOD2

NEUROG2

SELP

SELE

CCL3

TRIM21

S100A11

KLF6

SAA1

CCR5

COL11A2

CEACAM1

PSIP1

H3P40

COL4A2

SRY

XYLT2

SMTN

SPAG11A

SLC8A2

SUMO3

SMARCA4

SLC8A1

SLC8A3

SLC9A1

SLC19A1

SLC12A2

SUMO2

OLIG1

MIR544A

SFTPD

NMNAT1

P2RY12

ST3GAL4

NDRG4

KLK8

MGLL

OSBPL9

ADCYAP1R1

LRG1

BMP7

MAPK10

MCOLN1

PDIK1L

PTGER1

PTGER2

PTGIR

OR2AG1

MIR431

RNH1

PARP1

EDIL3

MIR376B

DENND11

RTN4

MAP2K1

MAP2K3

CTSG

PRNP

PSPN

PROS1

HTRA1

PSEN1

CLDND1

RETN

TIGAR

MIR490

MIR491

CRK

MOK

RAF1

IL21

ACE2

TRPV4

RBP4

ADORA2B

ACTB

CP

SLCO6A1

RPS6KB1

BAX

TRIB3

MIR146B

MIR497

NDRG3

NDRG2

SLC12A5

NLN

KIDINS220

SRXN1

PTMA

ADGRB2

PTMAP4

RNF213

RYR1

RIPK3

RNF146

GDF11

CDK5

KHSRP

CAV2

PSMG1

AOC3

ADAM10

CDK4

NRP1

CCK

CEBPZ

CYP4F2

PIK3R3

SNHG12

CAPN1

AXIN2

BRAP

CASP1

RAMP2

CASP4

CASP6

IMMP2L

AKT1S1

CAT

PPM1D

CDK9

ARHGEF7

PGR-AS1

SELENBP1

MAPK8IP1

CD36

SCARB1

GAL3ST1

VPS4B

ENTPD1

CXCL14

CARTPT

KLK4

CD68

ATG13

BMS1

MIR940

OPN4

MIR365A

HAP1

OTULIN

ANGPTL1

USP14

SLC33A1

CD14

S1PR2

OCLN

KL

LINC01672

GRAP2

MUC16

CDC42

PPP1R13L

CST12P

POSTN

TLR5

TM7SF2

TMSB4X

TXNIP

P3H4

TNFAIP6

TNFRSF1A

TNFRSF1B

HSP90B1

THPO

TSPO

RN7SL263P

CFTR

PLK2

TAT

NES

TBXAS1

TCF3

MIR634

TEAD1

TEK

TGFA

EBP

TGFBR2

SLC35A1

CERNA3

PERCC1

CACNA1B

SCG2

RAB7A

CAD

CALB1

NOX5

RAMP1

ADIPOR2

AIMP2

NLRX1

TFPI2

ZC3H12A

YWHAZ

AKR1A1

VWF

POTEF

ATG7

SEMA4D

UCHL1

UGCG

VPS51

SLC9A6

SNHG14

SPAG11B

VEGFC

SEMA3A

KLF2

COASY

CUX1

CXCL8

ITPR3

CBX5

JAK3

JUNB

JUND

GSTK1

KCND2

TARDBP

KCNK3

KCNN4

NCS1

ITGB1

ITGAM

IRAK1

FOSB

IL11

IL13

IL15

IL16

TNFRSF9

IL17A

RPL13A

IMPA1

CXCL10

PDX1

SIRT3

FLT1

LMTK2

MIR128-2

ABCD2

MIR140

MIR145

MIR146A

MIR149

FGA

MIR15A

MIR183

FCN1

MIR204

MIR106B

ARNTL

LTA

LAMP1

LAMC2

LBP

LCAT

SMG1

FOXF2

LGALS2

DKK1

LIF

LIG4

LIMK1

LPA

OPN5

IL4R

BTBD8

HPGDS

HCLS1

HFE

KCNIP3

GCLC

SIGLEC7

GJA1

ACACA

CBLIF

GH1

NPAS4

HOXC13

H2AX

GZMB

UBQLN1

ANGPT2

ANG

RGMB

GPI

GOT1

GLUL

GRIN2C

HTRA2

CXCL1

GSTM1

MSTN

HPX

TMEM119

APOBEC1

BRINP3

TXN2

IGF1R

ALOX15

RBPJ

ALOX5

ABCA4

SNHG1

IL2RB

PART1

RNF19A

APOB

APEH

HRG

APEX1

HES1

HSPA1A

HSPA1B

GDF1

HSPA5

GCY

FGF21

PTPN22

TNC

BACE1

EDN1

PCSK1

PCYT1A

DSCAM

DPYSL2

DCDC2

ISYNA1

PDK1

ENPP2

PDPK1

SERPINF1

STK26

GP6

HBEGF

ANGPTL4

ALDH7A1

P2RY2

AGTR2

DUOX2

PRDX1

DUSP2

SERPINB2

IL22

PRKN

TAS2R13

ASCC1

PER1

PFKFB3

PON2

PON3

CYP3A5

PPIA

ERCC6L

CASZ1

ADPRS

TUG1

CYP2C19

PREP

PRKCB

CYP4A11

TREM2

DUOX1

SERPINB6

SERPINE2

DIO2

ADAMTS18

DDC

MDK

DCR

BCL11A

SHC3

CYP11B2

SERPINF2

PLSCR1

BDNF-AS

PTK2B

MFGE8

MIF

KMT2A

EPHA4

MMP12

MMP14

CD200

MIAT

CDNF

MC4R

F12

MAS1

MIR212

MIR217

MIR224

MIR24-1

MIR29A

MIR29B1

MIR29B2

MIR320A

MIR93

SMAD3

MAG

MAPT

MIR133B

MIR323A

NOTCH1

EFNB2

NPPA

NPPB

NPR2

NRDC

ATP2B1

EEF1A1

EDNRB

NR4A2

MIR424

AHSG

ELANE

COX1

ENO2

ENO1

RNR2

MYCN

MYD88

GADD45B

ZBTB7C

ATF4

NUBP1

EMP1

EMD

ODC1

KRIT1

CCM2

COL4A1

TREX1

ENG

ATRIP

GAD1

ATRIP-TREX1

F13A1

GUCY1A1

ITM2B

MEF2C

SAMHD1

RNASEH2B

LRP8

ABCD1

ADD3

MEF2A

F3

SERPINA3

PSMA6

MPO

APOA1

ACTA2

GATM

CCT7

CLCN1

GCLM

PIK3C2A

PAFAH1B1

MB

ELN

BRAF

NF1

DCX

MMP3

PROCR

GP1BA

HBB

POLG

PLP1

MT-TL1

EPRS1

LRP1

PECAM1

ENPP1

INS

SERPINI1

PDGFRB

KRAS

FBN1

GBA

ADAMTS13

ARSA

HMGCR

SHH

THSD1

PCSK9

PDGFB

PSAP

WRN

PHACTR1

ADD1

NOS1

TNNT2

VLDLR

EGF

TSC2

AVP

ANXA5

GLA

CFH

ALG14

TNNI3

PKD1

PF4

ITGA2B

ERCC6

TIMP3

GSR

ITGA2

MIR34A

IFIH1

PSEN2

CETP

SLC20A2

CLU

PLA2G6

MEFV

STXBP1

TIMP1

GRIN1

DLL1

TSC1

CR1

VCAM1

CDKN2A

MME

SNCA

ABCC6

LRP6

ASPM

TH

THBS1

IDH1

ATP1A2

XPR1

FLNA

GRIN2B

TGFB2

MCPH1

F8

PPARA

HLA-B

FAS

JAM2

CALCA

MTR

CDH5

PRL

APOA2

DLL4

JAG1

KNG1

MPL

MYORG

LIPC

HTR2A

SORT1

COMT

RNF213-AS1

GSN

SPTAN1

APOC3

RNASEH2C

IL12B

RNASEH2A

FABP3

FCGR2A

CYP27A1

ADAR

DOCK8

CDKN2B

CD59

GPT

KCNMA1

PPBP

ADA2

PIGM

CYCS

SULT1A3

CCL11

HTT

GNAQ

ADRB1

LMNA

KCNQ1

CKB

CACNA1D

FN1

KCNJ5

CXCR4

CTSD

PROC

TTR

IDE

LOC107133510

LOC106099062

APOA5

BCHE

GJA4

CYBA

ACSL4

CYP2C9

GAPDH

SMPD1

LOC106627981

PIGW

CDH2

NOTCH2

A2M

CD34

MBP

TGFB3

SOD2

LOC102723566

MGP

LEMD3

IL6R

IDH2

IL33

CFLAR

CENPJ

PIGA

TGFBR3

GNB3

PTX3

ITPR1

CCR2

SLC4A1

NOTCH4

ZNF335

SCN1B

TERT

CSF1R

SYNGAP1

NTRK2

SST

CKM

F11

ANGPTL6

PAPPA

BRCA2

ABCC9

NKX2-1

EPOR

JAG2

ACTA2-AS1

EIF2B3

TCF4

SH2B3

BDKRB2

VCP

SPG7

IGFBP3

CALR

MT-CO1

HSPA8

TPP1

SCN8A

ACVRL1

GNB1

EIF2B5

RLBP1

CHKA

CDK5RAP2

NF2

YWHAQ

FLVCR2

NPC1

THBS2

MYH7

GYPA

HEY1

DLL3

CLCN2

GATA4

MYDGF

SHBG

TIMP2

CA3

LRRK2

ARSB

ADH1C

HEY2

TBK1

INSL6

CHAT

KCNQ2

C3

MECP2

SELL

VHL

ABCB1

EEF1A2

DYRK1A

HTR1A

KITLG

GHRL

ACP5

CTSB

B2M

TGFBR1

HEYL

SRRT

TOMM40

CPT2

PTCH1

PTH

MYH11

NSD1

SRC

ERCC2

KLF4

TBCD

GDF15

SERPINA1

LZTR1

ERCC1

PGF

THBS4

SMARCAL1

MT-CO3

RPS27A

ABCG5

MT-ND4

SMAD4

PCNT

ATL1

LAMB1

IGFBP1

CREBBP

RAB3GAP2

TBL2

COL1A2

FGFR2

TGIF1

SMO

MT-TF

APOA4

ITGAL

CNP

UTS2

MMP8

FCGR2B

WWOX

PCSK2

FGG

PDE5A

CDKN1C

ATP13A2

CIITA

TUBB3

HCN1

PTPN11

FURIN

FGF13

MYH6

NARS2

SLC2A10

DGUOK

C1S

NPPC

F9

CCN2

BGLAP

MDH2

SUCLA2

HLA-DRB1

DMXL2

SELPLG

PDHA1

IL12A

COL1A1

APCS

NCAM1

MIR199A1

SQSTM1

HSD17B10

MIR150

MIR133A1

ADSL

S100A1

CD55

FMR1

MT-CO2

EIF2AK2

PDGFA

RASA1

CD163

ADAMTSL1

TRPM4

MIR328

IL23R

ITGB2

PLCB1

ERCC8

ITM2C

CD46

MIR27A

ADORA2A

BAZ1B

SLC5A6

MAP1B

MLC1

TWNK

POLR1C

HFE-AS1

HABP2

PLAUR

BMP4

PVALB

ZMIZ1

MIR221

MMACHC

NEFL

PRKG1

PRTN3

CTC1

TANGO2

DNAJC5

RFC2

CTF1

LDLRAP1

CEP152

MIR22

MIR124-1

COG2

DCN

ABCG8

MSX2

AIFM1

MLX

CLIP2

CEP19

ARL13B

EHHADH

FKRP

ZMPSTE24

ARG1

CRH

DOCK6

DNASE1

RBFOX3

LMX1B

SDHB

SDHD

GTF2I

F13B

PIGQ

DISC1

STAT1

C4A

KRT18

DCC

TRAPPC9

MYL3

MIR181A1

CHGA

GJB2

SLC17A5

GABRA1

ZNF627

PC

MIR486-1

HSPD1

MMP13

ABCD4

VKORC1

KCNA1

GTF2IRD1

CDKN3

PCCB

ACTG1

TBP

GRK2

ATP7A

BACE2

ATXN2

MUC1

SORL1

NPR1

BMP6

NOTCH2NLC

GARS1

HRAS

XDH

EPAS1

MYO7A

STIL

CEP135

NTRK1

GOT2

TINF2

ZEB2

SOX2

TET2

IL27

AHDC1

PDP1

GRIA1

MMP7

SERPING1

TF

MT-TS1

RHOA

LOX

TIE1

MIR30A

RAB23

TMEM106B

TYMP

ISG15

CD4

MT-TK

FMN2

POMT2

UNC5C

OTC

PYGB

THOC2

CD8A

VPS33B

TNFRSF11B

HLA-A

LAMA2

EXOSC9

SDHA

NRXN1

MTRR

PRODH

HLA-DQB1

BRCC3

TNNT1

DMRT3

SCNN1A

MIR423

GAA

IL1R1

DNASE1L3

MLXIPL

AIMP1

COX14

AP5Z1

MYH9

CFI

TRAPPC11

DNMT1

ERCC4

CAMK2G

POMT1

AQP1

P2RX4

RAP1A

HTR3A

TMTC3

TOR1A

MT-ND2

GNRH1

POLG2

RAP1B

BAD

SHANK3

ISCA1

MEN1

MIR151A

CDH23

CNTF

LOC110806262

CCR1

LIPG

PARK7

BGN

GP1BB

PEX6

GRM1

EPHB4

RNF135

VIP

STK11

AFP

LYVE1

MIR17

MLEC

CCL7

DAG1

LMNB1

TUBB

P2RX3

CHIT1

IGF2

UQCRFS1

SURF1

H3-3A

ADCY10

GLI3

NBN

COL18A1

EP300

SUFU

IGFBP2

ASXL1

IL5

FKTN

SERPIND1

PGM1

H2AC18

MT-CYB

NAGA

ADAMTS4

DIAPH1

ITGAV

STN1

TLR3

RRM2B

POMC

MIR144

COX5A

ACTC1

CASP8

STRN

DNM1

CUX2

RAPGEF2

PAH

APC

CD79A

FABP12

ADORA3

GNAS

HBE1

MPV17

NKX2-5

IRS1

COL5A2

DNMT3A

PCCA

CYP7B1

TJP1

LEPR

ATP6AP2

CST4

FIG4

HLA-DQA1

LIPE

DKC1

ADCYAP1

ANXA2

COG5

MRE11

NRG1

SCARB2

TRH

RYR2

CACNA1C

LRP2

HDAC9

JPH3

ADA

TENM1

STAT4

HGD

SIL1

SLC25A4

ACADM

TFPI

VCAN

NAGLU

CTLA4

B3GALNT2

GABBR2

BRCA1

ADAM17

HIC1

SLC1A3

EXO1

DES

EOGT

PIGG

SUGCT

SIN3A

MIR143

PRKAR1A

POMGNT1

PRKDC

RAD51

LARGE1

DEAF1

GGCX

MVK

DYRK1B

PANK2

SCN9A

DPM2

MIR132

ERAP1

UBAC2

KLRC4

IL12A-AS1

RTEL1

ACADS

FLNC

SLC22A3

CXCR3

CASR

HIBCH

APOC1

ARF1

PRKCG

MAT2A

KCNA2

EIF2AK1

AGXT

AMPD2

IL17F

ABAT

MIR6886

HMGCL

RASIP1

ADAMTS1

ABO

GSTP1

NDUFAF3

MEST

PRDM16

ZFP30

DEL17Q11.2

CFB

GAP43

PTPRC

IRF3

CYP2J2

STRIP1

MAN2B1

PNP

MIR142

WNT3

GRIA4

MOCS2

TAF1

EPHA3

CD44

ALX4

CSF3R

CYP1B1

PTPA

IL3

IAPP

DLD

NSUN2

SKI

ECHS1

IFNA1

VPS13A

PIGT

FASLG

MT-TN

CA10

LOC107303340

CYP2D6

COL5A1

MAP2K2

TFAM

ATXN1

GSTT1

ASS1

CRPPA

IL1RAPL2

IVD

CGAS

DAPK1

NARS1

NEDD4L

NDUFS4

IKBKG

PIK3R2

MT-TL2

EZH2

KDM6A

MYT1L

GMPPB

PSTPIP1

PRKCA

CDH15

CCR6

NR1H2

SDC1

CSF1

HBG2

PARN

SDHC

AHSP

MYLK

IGHE

FAM20C

ACD

TMEM165

MT-TE

MPZ

FOXP3

RET

ABCG2

GCH1

LGMN

COQ2

SGCB

TAOK1

MSTO1

DDX58

PEX2

MOCS1

IFNB1

DGKZ

PLK4

DHCR24

TLR9

TARS1

MYMY4

MED12L

SCNN1B

SCNN1G

DNAJC6

TSPAN2

RNF13

GAL

DNM2

FCGR3B

HAMP

FGFR3

LTF

COG6

COL4A2-AS2

PTK2

SPARC

ANKIB1

CCNH

FDFT1

SYNJ1

SLC12A3

PHYH

FTO

COL2A1

IFIT1

STING1

KCNQ1OT1

NDST1

CYP2C8

VTN

CCR3

NRAS

NRGN

DBH

CALCRL

MAOB

LRRC56

FOXC1

SAA4

CYP7A1

HCRT

ALPL

SLC16A1-AS1

GPX1

OGDH

ALOX12B

NGFR

CSNK2B

GRM5

EGR1

WT1

PLA2G2A

QDPR

RPL36A-HNRNPH2

CHCHD10

CYP46A1

PIGV

COG7

EDC3

AHCY

GLUD1

ARMC8

ZNF621

KLHDC7A

ACAD9

PDYN

KCND3

CNTN1

LOC110806306

TK2

CDK5R1

WNT1

GABBR1

WASHC5

PIGY

M6PR

CALM1

HADHA

COL6A2

RPS6KA3

TBC1D7-LOC100130357

EIF4G1

CPS1

UGT1A1

CHD3

IDO1

PRUNE1

FGF23

OPA1

NFKBIL1

SOCS3

CHRNA4

SREBF2

CHI3L1

MASP2

NECTIN2

SLC6A6

UCP2

DDAH2

SOX17

CRYAA

PLTP

GC

HNF1B

NAA10

ACADVL

PIK3R1

ATXN3

RUNX2

TUBA1B

EFNA5

OSM

CAMTA1

PROM1

EHD1

NTS

GGT1

CEP63

MED23

XPA

PITX2

CNNM2

MMADHC

CYGB

HYOU1

HRH1

YAP1

MYBPC3

APOC2

DNAH8

STX1A

SLCO2A1

BECN1

IFITM5

ATP10A

SYNE1

SLC5A2

SPPL2A

TNFRSF4

NKRF

BCAR1

TREM1

LRPAP1

NT5E

IL6ST

ADAMTS7

SCN4A

NDUFA6

UFSP2

LMBRD1

CPOX

PNMT

IRS2

CARD16

GHSR

LOC109504728

RNASE3

HRH2

ACY1

PDCD1

NHP2

MAX

AIM2

SPTBN4

POMK

IL2RA

HS3ST4

POMGNT2

SLC19A2

AP3D1

FOXP2

DSP

ZRANB3

AAGAB

B4GALT1

MLH1

HLCS

SUN1

TPH1

ICOSLG

DOCK8-AS1

LDLR-AS1

LOC106560211

ATR

LRP5

CRYAB

TNIK

MT3

TERC

NAGS

SGK1

NSF

MYOCD

ETFA

EGLN1

CYP1A2

TFG

CDKN1B

GNA11

STAG1

NSUN5

DPM1

HYAL2

IFNGR1

NCF1

ABCB7

EIF2AK3

VCL

KRT7

KCNJ11

H19

IRF1

BIN1

FABP4

MINK1

ITM2A

ARID1A

ASAH1

CHEK2

FCGR3A

SMARCA2

HELLS

BLVRB

TNFRSF8

NEFH

PPP1R12A

MIR125B1

MIA3

TAC1

TGM2

IKZF1

CH25H

LMNB2

FUCA1

COL4A5

LAMP2

PET100

DYNC2H1

HTR2C

HDAC4

MIR125A

MKI67

TICAM1

GCKR

RECQL4

TAGLN

TNFSF12

BMP2

C5AR1

NDUFAF6

DMD

IL37

EFNB1

TNFSF11

RNU4ATAC

SERPINH1

GOSR2

ATP6V1E1

SLC18A3

RPA1

TBC1D7

FBLN1

MIR6795

AP2M1

TNFRSF11A

GJA5

HUWE1

APAF1

PDGFRA

PRF1

ALG2

TGM1

PCDH11X

PTGDS

DGKE

CYP27B1

GUSB

ABCG1

FBLN5

SUN2

CELSR2

SLC6A5

PAK1

CMC4

TRAPPC2L

LEPQTL1

NUP93

ERBB4

USP9X

PPARGC1A

WRAP53

NOP10

HARS1

DGKB

IL9

SLC34A1

TKT

ASCL1

NDUFA13

HRH3

RCAN1

BLZF1

HNRNPA1

ANPEP

DYSF

GSTO1

HOTAIR

RXFP2

TMEM127

NUDT6

POLB

CXCR2

MIR222

TNXB

HADHB

SMAD2

GPX3

HAVCR1

GIGYF2

FXN

STMN2

CHUK

SOX9

CTNNA1

MMUT

PIAS2

ALLC

LOC111365141

GPSM1

LTB

ADRB3

CDK1

ITIH4

CHRNB2

HBA2

SLC16A1

CCL18

THY1

PRKAA2

RNF207

PTGER3

PCSK5

IL18R1

CUBN

BCKDHB

CYP19A1

LOC107372315

RPLP0

CACNA1S

FYN

ANK3

CCL26

CAMK2D

PRKCE

ITGA3

CTSK

HTR1D

CAPN2

SLC25A24

HLA-C

DOLK

IL1RL1

XRCC1

ZFHX3

MIR26B

MMRN1

MROS

CD69

FTH1

CNR2

WNT9B

LMO4

CCR7

AGRN

FHOD1

FEN1

DNMT3B

CGB3

GATA2

KRT3

LAIR1

LAMA1

MYMK

HPGD

S100A8

IRF5

C19orf12

TRAF6

PROP1

NEU1

SDHAF2

SREBF1

DDX39B

NDUFA12

ZNF597

SBDS

ST2

AIP

STUB1

MYOM2

RECK

SHOC2

OXT

GLMN

TNFSF10

GRIK1

CXCL9

JCAD

PRKCD

ABCC2

ACAN

CPQ

TCN2

SMPD2

CYP2B6

HNRNPA2B1

WAS

DNA2

DCLK2

XYLT1

DDAH1

CCDC174

LBR

AGA

ITGA8

GRB2

GALNT2

CNTN5

GDF6

RORA

VASP

SERPINA4

TFEB

BCKDHA

PMP22

S1PR1

APLNR

DAB2IP

ACP1

MIR98

ITGA1

P4HB

ECM1

ERN1

IARS1

DGKQ

P2RY1

CTTNBP2

KIR2DL3

KCNE2

PLIN1

ITGA4

S100A9

CAV3

ALDH9A1

LDHA

BMPR1A

CD2

MYOD1

C4B

TNFSF13B

HMGCLL1

SLC5A3

CKMT1A

VDAC1

SLC18A2

DPM3

AMPD1

PYY

SHC1

AVPR2

USB1

TRIM9

TRPC3

DDIT3

MIR122

SYK

NCK2

VAMP8

PRORP

MMP10

S100A6

CARD14

FLNB

KIT

TRMU

OCRL

C1R

GATA1

PAX2

MGME1

FAM20A

ORC1

MSH6

SRFBP1

HSPB8

VIPR1

B4GAT1

TWIST1

ELP1

HLA-DPB1

SCN10A

IL12RB1

RECQL5

ABCC1

CACNB4

CTBP1

ACVR1

IGFBP4

FNDC5

FLAD1

TOLLIP

RASA2

FOLH1

TPO

HMOX2

DBN1

TRPS1

TUBA4A

PER3

SEMA3F

HAS1

PIEZO2

DEFA1

TMCC2

CCND2

ABTB2

PRPS1

PLCG1

NEB

LEF1

PLXNA2

ATP6V0A1

MCCC2

STAT6

GHRH

NISCH

LAMA3

REPS1

MIR99B

HPD

MTHFD1L

GPNMB

COG8

TNNC1

CAMK4

JUP

IKBKB

WARS1

TNFRSF10B

DHX37

APBA1

PTS

EXT1

TRIM5

MERTK

POU1F1

UCP3

LETM1

P2RY6

HNF4A

GPR182

GFRA1

EHD4

CXADR

CCDC115

APOM

TERF1

RIPK2

FLG-AS1

PIEZO1

CSRP3

IGFBP5

TPK1

KLK3

MITF

TSFM

NUTM2B-AS1

KALRN

PTPRD

NUBPL

POU2F3

CAPN3

MIR9-1

SIX5

PRKAG2

MIR16-1

ALOX12

CYP17A1

UCN

MIR133A2

SLC29A4

CDH13

GP5

ATF6

XK

ELAVL3

UROD

PLCB4

UBC

BMP1

RARB

STIM1

SHOX

RHOB

CLEC16A

CD63

C5

KLRK1

RNASEH1

ADH5

ROCK1

VSNL1

MRAS

RHOD

QKI

MIRLET7I

PTGER4

LIPA

SLC6A18

GLDC

POU5F1

CHD4

SNIP1

DOK7

ADGRB1

PIK3C3

LMAN1

MIR574

ARSJ

NR1H3

ANK2

NQO1

CALHM1

SLCO1B1

HSPA12B

GCSH

DHPS

MARK4

PRPF8

MIR26A1

ANK1

APIP

KCNK17

RAG1

LPAR1

MIR124-3

FADS2

DCTN1

FLRT3

ITGA9

TBX5

SH2B1

AMBP

SOAT1

IL10RA

BLOC1S1

TOX

FERMT3

PIGH

BSND

PTAFR

RO60

ALDH1L1

KRT5

TBX6

NT5C2

MYOT

ACTA1

FGR

LRP12

ARNT

LAMP3

C3AR1

ELAVL1

INHBA

BCR

CYSLTR1

HMGA1

lnc-ZNF296-6

NONHSAG026010.2

CANX

SLC52A2

MIRLET7F1

SRI

FKBP10

KRT20

GALNTL5

GALNT18

RAN

OXTR

AKAP10

SETX

NPHS2

POT1

CHRNA5

TWIST2

DIO3

C1QBP

INSIG2

APRT

ESM1

PLCL2

TBX20

CST2

POLD3

TJP2

NRIP2

CANT1

CD80

GHR

SYNE2

MMP16

MAPK9

B3GAT1

S100A12

TNFRSF10A

PDE8B

FGF1

SLC5A4

BTG2

CSNK1A1

EFEMP2

TTN

NR1H4

CLCN7

TAFA3

PSMB8

BMPR2

RER1

DHX16

LGALS1

GAS6

FHL5

MT-RNR1

XAB2

NPEPPS

SLC9A3R2

MIR19A

INF2

HTR2B

DYM

ITGAE

FLOT1

DUSP19

ADAMTS2

CELSR1

NOP2

KIR3DL1

IGES

XRCC5

KLHDC2

PCK1

MAZ

SYNPO

BCAM

SRR

BET1L

CDK2

GJC1

SLC39A7

EYA1

BPI

PRKAA1

BLM

ADAMTS3

ARVCF

COX20

CCT4

MIR186

P2RY11

MIR196A2

MLYCD

TERF2

ATP2B4

NONO

MAP3K14

NEAT1

AXL

PTN

XRCC6

ABCC4

DIABLO

PRKD1

BCL2L11

FBXO7

TTF2

CA9

NELFA

WDR36

PRMT5

CCT8

FABP7

GDF3

GLO1

LRRC37A2

VTI1A

GRP

MTERF1

HAX1

CLDN3

DHX9

CDKAL1

COL6A3

ATG14

AHR

F2RL1

ITGB6

SNHG3

SIX1

HNF1A

TRPV2

MACROD2

DVL1

MSR1

CD28

STAT5B

DDRGK1

AGK

CLDN1

FTMT

HIP1

CCDC86

PLA2G3

KAZN

CCDC22

DCD

KIAA0319L

XKR8

GABRA3

SEC24D

GP9

MIR24-2

GFER

CACNB2

PPP1R15A

RELB

AQP10

STAT5A

CYBRD1

KLC1

ITGA6

ACOT7

USP8

PHF6

B3GAT3

FOXF1

ARHGAP35

INTS8

SLC12A1

PNMA2

CXCL16

EVA1A

HSD3B7

KCNJ2

TRPV3

SUMO1

FHL2

DIP2C

GATA6

NLRP1

PLEKHG1

ETS1

MS

MIRLET7F2

HERPUD1

PPARD

GNAI3

C5AR2

PITRM1

ADAM8

MYB

BIRC2

CUL2

CACNB1

SMARCA1

SLC11A2

GRM8

SNX19

ALMS1

C1QTNF6

FOXO1

APOL1

SLC25A20

MARK3

PPIF

MIR128-1

ACP3

PIK3R5

BAZ1A

FDX1

MAP2K6

PXDN

CARF

EGLN3

SLC25A38

TLR6

SPATA13

SLC22A2

NTRK3

NPHS1

STAP1

TNFAIP3

SMARCD2

HPSE

SCAP

SERPINA9

LTBP1

SMPX

PSMC5

MAP2K4

FGFBP2

PSMA7

SMG6

SEMA3E

TLL1

TPPP3

MIR134

RNLS

DMTN

FAIM2

IFNA2

FPR2

PAK4

CYP11A1

SLC4A4

CYP4V2

TRPA1

BLVRA

ATP2A2

CNTN2

TNS1

SENP1

TTPA

QRSL1

GATAD1

KY

CASP2

SERPINA5

KCNE1

PTPRB

MEIS2

MAP3K1

PFDN4

REG1A

DNAJA3

MTHFD1

HEATR5B

MLANA

KCNJ8

CYP26B1

RYR3

PAFAH2

MIP

ITPK1

TRIM63

SIGLEC5

DPP6

XRCC3

MYOG

MASP1

LOC110973015

ASZ1

BAG3

EXT2

RHD

TAF3

WDR31

MAPRE2

MIR361

MIR485

ACKR2

MARCHF1

ABL1

NR5A1

MIR197

NAT2

CFP

EPX

IGF2R

KCNK5

PIGF

WNT5A

FAAH

NPTXR

IGFBP7

MDH1

RBFOX1

SLC7A8

PML

DPAGT1

POLE3

YWHAB

P2RX7

CYP2E1

C4BPA

ZFYVE1

MCU

U2AF1

ALK

AP3B1

FKBP15

ANKH

MNX1

SERPINB8

SLC26A8

HLA-DPB2

SPRY1

MMAA

FAP

CNBP

MBNL1

MICA

ACADSB

A2ML1

ABCA3

OPRK1

CCDC180

SMARCA5

MIR139

KCNE3

ATP2B2

TCF12

CPT1B

TP53BP1

PTGFR

TLR7

IFIT2

FOXA2

ORAI1

RAB11FIP4

COPRS

MARK1

MGAM

FDPS

LAMB2

ZFPM2

BACH1

HADH

HTR4

MAFB

CTSZ

ARHGAP42

TMEM63B

SRSF2

TRDN

AGL

TP73

LPAR3

OGG1

ATG5

ABITRAM

PLEC

GLRX

PROKR2

BIRC3

SIRT6

TNNI3K

APOC4

TNRC18

APOC1P1

MIR5702

ENSG00000235070

LINC02514

ENSG00000225233

LINC02515

LOC102723536

ENSG00000254347

ENSG00000228255

RN7SL408P

RN7SL14P

RF00017-5493

lnc-APOC1-1

GGCTP2

ENSG00000280087

RF00017-5491

HSALNG0055868

HSALNG0048493-001

piR-38722

piR-56133-114

ENSG00000212989

piR-61028-297

RF00017-2706

SMOX

MYPN

ECRG4

BUD23

VDAC2

GALNT17

ANAPC5

SIRPA

BTK

CNTLN

TCP1

CHKB

EWSR1

TLR1

SERPINA7

NPC1L1

PEA15

TIPARP

TMSB15A

PTTG1

MEOX2

SCGN

SMAD9

ARHGEF2

ITGA7

PODXL

CACNB3

TRPC4

PHEX

IL32

CASC15

TLN1

GIMAP4

GIMAP2

GAB2

EYA4

MNT

OPCML

CMA1

ENHO

VTI1B

MTNR1A

KRT8

ASTN1

ACVR1B

DKK2

SOX2-OT

KLKB1

NEXN

ALDH1A2

CD177

SMG8

USF1

SECISBP2

P2RX1

BCL2A1

PDLIM1

PI4KB

SPTBN5

NUP205

CLEC4A

CD274

RLN2

CCL22

PHYHIP

KRT14

MIR100

RAD54L

RAD52

RECQL

SELENON

MCC

LTBP4

HLA-E

ATP10D

OGT

PER2

DMBT1

DHX36

CD2AP

RND3

NSUN3

RAMP3

PIK3C2G

GYS1

NRBF2

PRDX2

TMLHE

TENM3

AKT2

COX5B

TYK2

MATR3

PYROXD1

CCR4

NOS1AP

CD47

XIST

CHGB

LAG3

EDN2

TUBB1

BDKRB1

JMJD1C

PITPNA

SLCO3A1

F2RL2

MIR496

OTX1

PENK

BDH1

IFNGR2

CES1

MT1A

RYK

SLC2A9

PIM1

UBE2T

EIF3L

C1orf116

MAP1LC3B

ZNF607

MIR15B

RUNX1T1

TCF7L2

CBL

CXCL13

MARCHF10

PTPN3

MIR103A1

CPA6

SPHK2

SLC15A1

MIR191

GPR55

MAPKAPK2

GAS7

SLC22A8

GMDS

PLA2G5

INPP5K

MUS81

HLTF

BLMH

PADI2

ATF2

SETD1B

ITLN1

PPM1K

COL6A1

KCNN1

BNIP3

FZD9

PRKAB1

NOL3

RSPH6A

CLEC3B

AZIN2

NCOR2

NFATC1

TRAP1

IL1R2

MIR33A

IFI27

GYG1

ADAMTS5

ADAMTS17

MIR192

MIR185

MIR23B

WRNIP1

PURG

ZNF787

PLCB3

MICU1

CADM1

FES

GINS2

PIDD1

GATD3A

XPNPEP1

INTS5

SMAD1

HSP90AB1

PPP1R14A

NDFIP1

TMEM80

SOCS1

BANF1

ABT1

SPRED1

SETDB1

ARHGAP15

GTDC1

PRDM8

RBSN

NOM1

SLN

PROCA1

PDPN

UMOD

PLA2G10

CTTN

RMST

GSTZ1

PI3

CCL24

CCL17

SH3PXD2B

BCL2L14

ATP8A1

PTHLH

NSMCE2

BID

SP3

USF2

MS4A6A

TERF2IP

HSPA12A

MIR455

MTF1

KIR2DL1

BMPR1B

PZP

LIMK2

FAH

EIF4H

PGK1

CAPN10

C1QA

PAX7

TBX4

AGGF1

SAG

LOC646736

HNRNPDL

SS18L1

SLC34A3

ABCB11

CAMP

HOMER1

ITGB5

CLOCK

CAST

ITGAX

SORCS1

ALDOA

SOS1

SOS2

TIPIN

CSH2

RADX

CNOT3

CCT2

TMOD2

NR0B1

SFN

TLR8

PANX2

WDR37

SPTB

HSD11B2

DIP2B

CTSH

IL12RB2

MIR331

NFE2

UBR3

IRAK4

RPP25

CDS2

DUSP1

ALDOB

SFRP1

ST6GALNAC1

SERPINA12

HBS1L

CLCN3

ATP5F1E

TNFSF8

EVI2B

VEGFB

PDGFD

MSI1

MYH10

PKM

CIB1

SLC29A1

CPLX2

MYH14

CMIP

MOB2

RBCK1

HACD1

MIEF2

GJB4

PKHD1

SMURF2

UTP6

EFHC2

CD151

SPRR1B

S100A4

ANO5

DSTYK

HLA-DRA

GALNT3

ENPP3

HYAL1

SGCA

MBD4

SNX9

TMPO

BAK1

NEK9

RAB11B

DPYSL4

UCP1

DPYSL5

MIR382

MMAB

ITGB4

GRIN3B

MIR30D

PF4V1

FGF5

DRD4

ST8SIA2

HIGD1A

PIP5K1A

AOC1

NUB1

MAP6

KCNMB1

IFT81

CXCR5

PSMA4

CDK20

SLC25A14

OGA

MAP3K2

TBX21

FHIT

SHROOM3

CACNA1F

MIR495

CFHR2

S1PR3

SBF1

GAST

CXCL2

RUNX1

ID1

RPL13

TAF4

FST

RGS18

BLID

CYSLTR2

CLDN4

SHOX2

ANKRD26

ENO3

CYP21A2

MIR148B

SCAMP2

ADAMTS9

COQ8B

RRAS

OMA1

TMSB10

ADGRL3

FHL1

HAND2

TRIM32

TIMM22

INPP5B

AURKAIP1

COPS8

GIMAP7

CDC123

SCAMP5

ACTRT2

TCHP

C15orf39

PKD1L3

HTT-AS

NSUN6

NSUN7

IL5RA

BTRC

HLF

ECI2

MTDH

MIR23A

FGF4

VAMP7

SMCHD1

TAFAZZIN

SORBS3

SUMO4

JPH4

RXFP3

WNT2

GTF2IRD2

CCL20

CEACAM3

MIR10A

DNAJB6

BCAN

ZNF185

TBL3

PHOSPHO1

ELOVL5

C7

STC2

MIR16-2

MIR194-1

STAG3L4

ANXA11

C16orf95

ADD2

PARP2

ACTN4

RSAD2

PRG2

GNE

TIA1

COLEC11

SLC27A4

ILVBL

DGCR8

HAS2

C12orf75

MTMR3

NME7

USP16

PRIM2

PPCDC

B3GNT3

FGD5

EHBP1

CPLX3

DUSP18

ACTR10

TSPAN14

ZHX3

ELFN1

PHACTR4

SLC35E4

ARMH4

PTPN6

HPR

NR2C2

SH3PXD2A

MAGI3

MTTP

ACTR3

RFX3

DDIT4

SLIT2

SLC52A1

NAALADL2

STAG3

NIF3L1

ELOB

TRIM50

TNKS

OBSCN

TG

PRKCQ

IRAG1

COMP

CDH4

TRDMT1

LDB3

BARD1

ALDOC

LTBP2

UHRF1

MYO7B

HCN4

TREML1

WNT10B

ZNF224

NT5C1B

CTH

JAK1

MAVS

HLA-G

CRLF3

DYNLT1

LOC100507006

PDE1A

NPR3

CALM2

PLA2G2D

COQ5

IFITM3

IFITM1

OAS3

VAMP5

CTR9

EYA3

PAPPA2

IFITM2

ARHGEF26

RASGEF1B

SLC46A2

MRPL9

DDI1

CACFD1

TRNAU1AP

ATXN7L3B

TOMM20L

GCN1

GPR65

MIRLET7A1

ITGB8

DNAJC21

COA7

MICB

MTARC1

TFAP2A

WNK3

CD58

THRB

ITGAD

MIR211

SLC22A4

BPGM

INHA

RPS9

LILRB3

PSORS1C1

MIR199B

MIR154

ATP6V0A4

HK2

DST

LTC4S

GFPT1

SOCS2

UBR5

LAT2

FKBP6

CETN3

FIP1L1

ENAM

MXD1

ABHD11

BCL7B

MXD4

ATAD5

DNAJC30

VPS37D

GTF2IRD2B

TRIM74

POM121C

TRIM73

SPDYE1

METTL27

RCC1L

TMEM270

STAG3L1

NSUN5P2

STAG3L2

NSUN5P1

SPDYE7P

STAG3L3

ABHD11-AS1

LINC00851

GTF2IP1

GTF2IRD2P1

GTF2IP4

SPDYE14

SPDYE8

SPDYE15

SPDYE12P

SPDYE10P

SPDYE13

EIF4HP1

SPDYE9

WBSCR2

WBSCR23

LOC106029312

LOC108228208

LOC108228209

LOC106029311

LOC106029313

CCND3

SCRN1

TPM1

NOG

FUT4

BCKDK

MCEE

RPS3

PSMA3

EIF4A3

SF3A1

HAPLN3

YTHDF3

PNPLA2

WWP2

PDLIM5

ZC3HC1

GNAI1

ITGA5

RPL11

SPPL3

MIRLET7C

FSTL1

ABCG4

ADAMTSL5

HLA-S

TNKS2

GPR68

MTA1

GOLM1

RBP3

ITPKC

POU4F1

MATN3

MAPKAP1

CLEC7A

ACTN2

MIR30E

CCNY

KCNN3

E2F1

CORO1A

MIR766

PLOD2

DBNL

PANK4

NEUROD1

SLC17A1

PROK2

TGFBI

VIPR2

HSD11B1

F11R

RUVBL2

PARG

PTPRN2

AKAP12

RGS12

EPCAM

IRF8

MMP20

MCM6

ADH1A

EXOSC10

CD52

ADAP2

EVI2A

ELOC

LRRC37B

TEX45

MIR1304

LOC642361

AGFG1

SPTBN1

NINJ2

MAP1LC3A

PYGM

PHKA2

COL9A3

TRIP4

GATC

RAPGEF3

GALNT13

MAP3K12

A4GALT

ICMT

RCE1

EGFL7

THUMPD3-AS1

ARSL

IRF9

SKIV2L

BLK

GGACT

GLOD5

TSPAN9

TAP2

TPM3

PABPN1

GSTA2

PLCG2

MIB1

FOXC2

MAP3K6

SASH1

FOXL1

ZNF536

ZNF318

IPO9

CTAGE1

SH2D4B

MIR342

TNFRSF25

PTPRA

RPL4

COTL1

PTPRH

AGBL4

NCEH1

CD93

SLC2A13

ACAD8

CARD9

ROR2

POLR1D

IL21R

ACSS1

WNK2

CUL9

NCK1

CD86

ADCY6

BCAT1

PYCARD

PDIA2

PDK3

ISCU

CFL2

PGAM2

CAP2

ATP5F1D

COL13A1

PACS2

SYNCRIP

MAPK7

MIR3149

MIR324

ESRRA

THRA

MIR206

CAMK1D

DEGS2

PDE2A

CAP1

MOAP1

MIR34C

IL7R

TXK

RORC

ATP12A

BST1

ATP4A

SLC2A6

EPN1

CRYGD

CPVL

SRSF6

ERAP2

CEACAM8

TRIM39

KLC4

NELFCD

SYNE3

THYN1

IGLON5

JRKL

ANKRD20A1

ZNF98

C1orf141

CBARP

MIR148A

CNC2

SLC22A6

BAIAP2L1

PPP1R10

RFC1

SPINT1

ITGB7

CSMD1

INTS11

DAXX

CSN3

ERP44

AK1

LPAR2

HSPB7

MTFR1

CPT1A

SGCD

DMGDH

SGCG

PLEKHG5

TMEM43

FKBP14

KLHL41

FDX2

KCNJ18

LRP1B

DNM3

NTN4

BVES

CD226

MRGPRX2

MEF2D

HDAC5

BMP15

FGF7

SYTL4

DRP2

NIT2

MIR335

SSBP3

INSIG1

BBC3

MIR125B2

LYN

FKBP5

FUT2

CHL1

CRAT

CSF2RA

ITGA11

IFRD1

DPP10

ITGA10

CLEC12B

PUM3

MIR215

MIR4301

LOC102724020

CARM1P1

THRB-AS2

PLN

TOR1AIP1

TCAP

TBX18

CTPS1

EIF4A2

ASRGL1

SNU13

SULT2B1

TP53INP1

FUT7

F2RL3

NPTX1

HJV

EID1

LCN1

SEMA7A

ZWINT

KCNA3

RGS8

AVPR1A

HCN2

ANXA4

GIT1

PTGES3

RPS11

SCUBE1

TFF1

TMEM39A

BYSL

SEPTIN9

ZNF385D

LARGE2

INTS4

PLCE1

RASSF1

MRAP

C1QTNF1

FRZB

GEN1

PROK1

GATA3

CCN1

CERKL

TCF25

PARP3

PPP1CA

ATP5F1A

JMJD6

LGALS9

TOMM70

MIR135B

RBMS1

AGBL1

SLC4A3

CEP112

MIR340

GBF1

ARHGAP6

BAG6

ACYP2

SORCS2

PRKCI

PRDM5

HSPE1

SUPT3H

RPLP1

CELF4

NHLRC1

TPM2

ANKRD1

GATB

GIPR

PDXP

RAB37

ADCY1

CD5L

TSPAN32

CLLU1

FARP2

ATP5IF1

MIR216A

HSF1

SLC22A12

STK39

GJA3

ST13

EIF5B

KLLN

DUSP6

TMEFF2

CCL21

PTPRJ

AZU1

DOCK1

PSMB4

UBE2R2

NADSYN1

LLGL2

USP20

NUP210

ERGIC1

PPP1R12B

NVL

OGDHL

ZNF79

FAM13A

DIDO1

USP37

COL23A1

OSGIN1

CDYL2

SDK1

MEGF11

RABGAP1L

GPR160

MON1B

ZNF618

PNLDC1

RAB6C

ZC3H3

ZSWIM2

ELK1

MIRLET7G

MIR590

AVEN

TFR2

MEPE

TCF15

KCNN2

ESRRG

NDRG1

HGFAC

MIR20A

CHD9

PRPS1L1

PDZK1

PRCP

MCTP2

GRK5

PCDH7

VEGFD

ENDOG

SLPI

PTPN2

SLC9A3R1

AASS

COL14A1

LPIN2

ASAP1

DOCK4

RABEP1

SPON1

TXNL4B

TC2N

SYNRG

RPS6KB2

UTS2R

CSRP1

RBBP5

CDC5L

GRIPAP1

H2BC3

CD74

AK3

MS4A2

REEP5

GAS2

IRX4

PPP1R17

BCO1

ENDOD1

RARRES2

CHRNB4

B3GAT2

GPR135

IGKV2D-29

SSH1

EPS15

TRIP10

MAGED2

PARP10

ACLY

MARK2

LAP3

SH3GL1

GLG1

ELF5

MIR345

PVT1

DOK1

LASP1

EIF4G3

CRY2

BNIP2

COPZ1

LIMA1

CERS2

PMAIP1

RTN1

SRP68

DCTN6

DTD1

TSC22D4

SEPTIN5

BEX4

MAPK13

NR1D1

ARRB1

GRK3

CEL

LRAT

BHMT

MAT2B

ARHGEF3

PRDX5

GSTA1

NRG3

SULT1A1

SLC17A3

UGGT2

EMC8

VOPP1

TAFA4

PRKG2

ZYX

ALDH4A1

RPS14

ACAA2

ACSS2

XPNPEP2

SPI1

MYO9B

LCP2

STX4

HPS4

NRBP1

SNRK

TOMM20

ANKRD12

ERBIN

SVIP

CMYA5

HIF1A-AS1

HLX-AS1

IMPA2

PKP2

KCNE5

MYO18B

NEK2

CEBPA

MTNR1B

NOD1

CRTC1

MAP4

KIF13B

CLASP1

TAGLN2

SRGAP3

RPS16

ZDHHC8

TMX1

DOK6

PTDSS2

IFNE

FPR1

NNMT

HSPA1L

KIR2DL4

CFAP251

MIR433

MIR30B

GNAI2

NFAT5

ABI1

PTPRG

FMO1

TLK1

MEIS1

MLKL

DNAJB9

KCNIP4

RUFY3

RPL35AP35

RPL23AP28

ITPKB

SERPINA6

HMMR

FBXO25

TFPT

ITPRID1

FLT3

NR5A2

NR0B2

S1PR4

AGO2

HDGF

ATG4B

TCN1

WDR1

KIF16B

FLRT2

GMEB1

NBPF3

SEPTIN2

SELENOS

MIR203A

TTC4P1

HBB-LCR

PTGS1

CHRM3

CHRM1

SCN5A

PTGS2

CA2

RXRA

OPRD1

PDE3A

ADRA1A

CHRM2

ADRA1B

SLC6A3

ADRB2

OPRM1

HSP90AA1

CHRNA7

DRD1

SLC6A4

PGR

NR3C2

ACHE

DRD2

F2

ESR1

AR

PPARG

DPP4

F10

PTPN1

AKR1B1

RELA

EGFR

AKT1

CCND1

BCL2L1

CDKN1A

CASP9

MMP2

MMP9

MAPK1

IL10

RB1

JUN

IL6

CASP3

TP63

NFKBIA

TOP1

MDM2

APP

MMP1

PCNA

HMOX1

CASP7

ICAM1

MCL1

BIRC5

IL2

CCNB1

TYR

IFNG

IL4

SLC2A4

INSR

CD40LG

PTGES

MET

CCNA2

ADRA2A

ADRA2C

NOS2

ESR2

MAPK14

GSK3B

NR3C1

KCNH2

STAT3

EDN3

F7

SLC6A2

ADRA2B

BCL2

FOS

AHSA1

EDNRA

CYP3A4

MYC

CYP1A1

NR1I2

NPM1

ECE1

PARP4

ITGB3
