# Supplementary material for: Molecular Mechanism of Salvia miltiorrhiza Bunge in Treating Cerebral Infarction
Source: Evid Based Complement Alternat Med. 2022 Mar 29;2022:5992394. doi: 10.1155/2022/5992394 (PMC8983215; doi:10.1155/2022/5992394)
Supplement: Supplementary Materials — Table S1: details of active compounds and targets. Table S2: known therapeutic targets in cerebral infarction. Table S3: the details of the PPI network. Table S4: the details of the GO analysis. Table S5: the KEGG pathway enrichment analysis. [file 5992394.f1.zip › 5992394.f1/Table S3- The details of the PPI network (1).doc]

**Table S5: The details of the PPI network**

| **node1** | **node2** | **combined_score** |
| --- | --- | --- |
| ACHE; ACHE; ACHE; ACHE; ACHE; ACHE; ACHE; ACHE; ACHE; ACHE; ACHE; ACHE; ACHE; ACHE; ACHE; ACHE; ACHE; ACHE; ACHE; ACHE; ACHE; ACHE; ADRA1A; ADRA1A; ADRA1A; ADRA1A; ADRA1A; ADRA1A; ADRA1A; ADRA1A; ADRA1A; ADRA1A; ADRA1A; ADRA1B; ADRA1B; ADRA1B; ADRA1B; ADRA1B; ADRA1B; ADRA1B; ADRA1B; ADRA1B; ADRA1B; ADRA1B; ADRA2A; ADRA2A; ADRA2A; ADRA2A; ADRA2A; ADRA2A; ADRA2A; ADRA2A; ADRA2A; ADRA2A; ADRA2A; ADRA2B; ADRA2B; ADRA2B; ADRA2B; ADRA2B; ADRA2B; ADRA2B; ADRA2B; ADRA2B; ADRA2C; ADRA2C; ADRA2C; ADRA2C; ADRA2C; ADRA2C; ADRA2C; ADRA2C; ADRA2C; ADRB2; ADRB2; ADRB2; ADRB2; ADRB2; ADRB2; ADRB2; ADRB2; ADRB2; ADRB2; ADRB2; ADRB2; ADRB2; ADRB2; ADRB2; ADRB2; AHSA1; AHSA1; AHSA1; AKR1B1; AKR1B1; AKR1B1; AKR1B1; AKR1B1; AKR1B1; AKR1B1; AKT1; AKT1; AKT1; AKT1; AKT1; AKT1; AKT1; AKT1; AKT1; AKT1; AKT1; AKT1; AKT1; AKT1; AKT1; AKT1; AKT1; AKT1; AKT1; AKT1; AKT1; AKT1; AKT1; AKT1; AKT1; AKT1; AKT1; AKT1; AKT1; AKT1; AKT1; AKT1; AKT1; AKT1; AKT1; AKT1; AKT1; AKT1; AKT1; AKT1; AKT1; AKT1; AKT1; AKT1; AKT1; AKT1; AKT1; AKT1; AKT1; AKT1; AKT1; AKT1; AKT1; AKT1; AKT1; AKT1; AKT1; AKT1; AKT1; AKT1; AKT1; AKT1; APP; APP; APP; APP; APP; APP; APP; APP; APP; APP; APP; APP; APP; APP; APP; APP; APP; APP; APP; APP; APP; APP; APP; APP; APP; APP; APP; APP; APP; AR; AR; AR; AR; AR; AR; AR; AR; AR; AR; AR; AR; AR; AR; AR; AR; AR; AR; AR; AR; AR; AR; AR; AR; AR; AR; AR; AR; AR; AR; AR; AR; AR; AR; BCL2; BCL2; BCL2; BCL2; BCL2; BCL2; BCL2; BCL2; BCL2; BCL2; BCL2; BCL2; BCL2L1; BCL2L1; BCL2L1; BCL2L1; BCL2L1; BCL2L1; BCL2L1; BCL2L1; BCL2L1; BCL2L1; BCL2L1; BCL2L1; BCL2L1; BCL2L1; BCL2L1; BCL2L1; BCL2L1; BCL2L1; BCL2L1; BCL2L1; BCL2L1; BCL2L1; BCL2L1; BCL2L1; BCL2L1; BCL2L1; BCL2L1; BCL2L1; BCL2L1; BCL2L1; BCL2L1; BCL2L1; BCL2L1; BCL2L1; BCL2L1; BCL2L1; BCL2L1; BIRC5; BIRC5; BIRC5; BIRC5; BIRC5; BIRC5; BIRC5; BIRC5; BIRC5; BIRC5; BIRC5; BIRC5; BIRC5; BIRC5; BIRC5; CA2; CASP3; CASP3; CASP3; CASP3; CASP3; CASP3; CASP3; CASP3; CASP3; CASP3; CASP3; CASP3; CASP3; CASP3; CASP3; CASP3; CASP3; CASP3; CASP3; CASP3; CASP3; CASP3; CASP3; CASP3; CASP3; CASP3; CASP3; CASP3; CASP3; CASP3; CASP3; CASP3; CASP3; CASP3; CASP3; CASP3; CASP3; CASP3; CASP3; CASP3; CASP3; CASP3; CASP3; CASP7; CASP7; CASP7; CASP7; CASP7; CASP7; CASP7; CASP7; CASP7; CASP7; CASP7; CASP7; CASP7; CASP7; CASP7; CASP7; CASP7; CASP9; CASP9; CASP9; CASP9; CASP9; CASP9; CASP9; CASP9; CASP9; CASP9; CASP9; CASP9; CASP9; CASP9; CASP9; CASP9; CASP9; CASP9; CASP9; CASP9; CASP9; CASP9; CASP9; CASP9; CASP9; CASP9; CASP9; CASP9; CCNA2; CCNA2; CCNA2; CCNA2; CCNA2; CCNA2; CCNA2; CCNA2; CCNA2; CCNA2; CCNA2; CCNA2; CCNA2; CCNA2; CCNA2; CCNA2; CCNA2; CCNA2; CCNA2; CCNB1; CCNB1; CCNB1; CCNB1; CCNB1; CCNB1; CCNB1; CCNB1; CCNB1; CCNB1; CCNB1; CCNB1; CCNB1; CCNB1; CCNB1; CCNB1; CCNB1; CCNB1; CCNB1; CCNB1; CCNB1; CCND1; CCND1; CCND1; CCND1; CCND1; CCND1; CCND1; CCND1; CCND1; CCND1; CCND1; CCND1; CCND1; CCND1; CCND1; CCND1; CCND1; CCND1; CCND1; CCND1; CCND1; CCND1; CCND1; CCND1; CCND1; CCND1; CCND1; CCND1; CCND1; CCND1; CCND1; CCND1; CCND1; CCND1; CD40LG; CD40LG; CD40LG; CD40LG; CD40LG; CD40LG; CD40LG; CD40LG; CD40LG; CD40LG; CD40LG; CD40LG; CD40LG; CD40LG; CD40LG; CD40LG; CD40LG; CD40LG; CDKN1A; CDKN1A; CDKN1A; CDKN1A; CDKN1A; CDKN1A; CDKN1A; CDKN1A; CDKN1A; CDKN1A; CDKN1A; CDKN1A; CDKN1A; CDKN1A; CDKN1A; CDKN1A; CDKN1A; CDKN1A; CDKN1A; CDKN1A; CDKN1A; CDKN1A; CDKN1A; CHRM1; CHRM1; CHRM1; CHRM1; CHRM1; CHRM1; CHRM1; CHRM1; CHRM2; CHRM2; CHRM2; CHRM2; CHRM2; CHRM2; CHRM3; CHRM3; CHRM3; CHRM3; CHRNA7; CHRNA7; CHRNA7; CYP1A1; CYP1A1; CYP1A1; CYP1A1; CYP1A1; CYP1A1; CYP1A1; CYP1A1; CYP1A1; CYP1A1; CYP1A1; CYP1A1; CYP1A1; CYP3A4; CYP3A4; CYP3A4; CYP3A4; CYP3A4; CYP3A4; CYP3A4; CYP3A4; CYP3A4; CYP3A4; CYP3A4; DPP4; DPP4; DPP4; DPP4; DPP4; DPP4; DPP4; DPP4; DRD1; DRD1; DRD1; DRD1; DRD1; DRD2; DRD2; DRD2; DRD2; DRD2; DRD2; DRD2; ECE1; ECE1; ECE1; EDN3; EDN3; EDN3; EDN3; EDN3; EDN3; EDNRA; EDNRA; EDNRA; EDNRA; EDNRA; EGFR; EGFR; EGFR; EGFR; EGFR; EGFR; EGFR; EGFR; EGFR; EGFR; EGFR; EGFR; EGFR; EGFR; EGFR; EGFR; EGFR; EGFR; EGFR; EGFR; EGFR; EGFR; EGFR; EGFR; EGFR; EGFR; EGFR; EGFR; EGFR; EGFR; EGFR; EGFR; EGFR; EGFR; EGFR; EGFR; ESR1; ESR1; ESR1; ESR1; ESR1; ESR1; ESR1; ESR1; ESR1; ESR1; ESR1; ESR1; ESR1; ESR1; ESR1; ESR1; ESR1; ESR1; ESR1; ESR1; ESR1; ESR1; ESR1; ESR1; ESR1; ESR1; ESR2; ESR2; ESR2; ESR2; ESR2; ESR2; ESR2; ESR2; ESR2; ESR2; ESR2; F10; F10; F10; F2; F2; F2; F2; F2; F2; F2; F2; F2; F2; F2; F2; F7; FOS; FOS; FOS; FOS; FOS; FOS; FOS; FOS; FOS; FOS; FOS; FOS; FOS; FOS; FOS; FOS; FOS; FOS; FOS; FOS; FOS; FOS; FOS; FOS; FOS; FOS; FOS; FOS; GSK3B; GSK3B; GSK3B; GSK3B; GSK3B; GSK3B; GSK3B; GSK3B; GSK3B; GSK3B; GSK3B; GSK3B; GSK3B; GSK3B; GSK3B; HMOX1; HMOX1; HMOX1; HMOX1; HMOX1; HMOX1; HMOX1; HMOX1; HMOX1; HMOX1; HMOX1; HMOX1; HMOX1; HMOX1; HMOX1; HMOX1; HMOX1; HMOX1; HMOX1; HSP90AA1; HSP90AA1; HSP90AA1; HSP90AA1; HSP90AA1; HSP90AA1; HSP90AA1; HSP90AA1; HSP90AA1; HSP90AA1; HSP90AA1; HSP90AA1; HSP90AA1; HSP90AA1; HSP90AA1; HSP90AA1; HSP90AA1; HSP90AA1; HSP90AA1; HSP90AA1; HSP90AA1; HSP90AA1; HSP90AA1; HSP90AA1; HSP90AA1; ICAM1; ICAM1; ICAM1; ICAM1; ICAM1; ICAM1; ICAM1; ICAM1; ICAM1; ICAM1; ICAM1; ICAM1; ICAM1; ICAM1; ICAM1; ICAM1; ICAM1; ICAM1; ICAM1; IFNG; IFNG; IFNG; IFNG; IFNG; IFNG; IFNG; IFNG; IFNG; IFNG; IFNG; IFNG; IFNG; IFNG; IFNG; IFNG; IFNG; IFNG; IL10; IL10; IL10; IL10; IL10; IL10; IL10; IL10; IL10; IL10; IL10; IL10; IL10; IL10; IL10; IL10; IL10; IL10; IL10; IL10; IL2; IL2; IL2; IL2; IL2; IL2; IL2; IL2; IL2; IL2; IL2; IL2; IL2; IL2; IL2; IL2; IL4; IL4; IL4; IL4; IL4; IL4; IL4; IL4; IL4; IL4; IL4; IL4; IL4; IL4; IL4; IL4; IL4; IL6; IL6; IL6; IL6; IL6; IL6; IL6; IL6; IL6; IL6; IL6; IL6; IL6; IL6; IL6; IL6; IL6; IL6; IL6; IL6; IL6; IL6; IL6; IL6; IL6; IL6; IL6; IL6; INSR; INSR; INSR; INSR; INSR; ITGB3; ITGB3; ITGB3; ITGB3; ITGB3; JUN; JUN; JUN; JUN; JUN; JUN; JUN; JUN; JUN; JUN; JUN; JUN; JUN; JUN; JUN; JUN; JUN; JUN; JUN; JUN; JUN; KCNH2; MAPK1; MAPK1; MAPK1; MAPK1; MAPK1; MAPK1; MAPK1; MAPK1; MAPK1; MAPK1; MAPK1; MAPK1; MAPK1; MAPK1; MAPK1; MAPK1; MAPK1; MAPK1; MAPK1; MAPK1; MAPK1; MAPK14; MAPK14; MAPK14; MAPK14; MAPK14; MAPK14; MAPK14; MAPK14; MAPK14; MAPK14; MAPK14; MAPK14; MAPK14; MAPK14; MAPK14; MAPK14; MCL1; MCL1; MCL1; MCL1; MCL1; MCL1; MCL1; MCL1; MCL1; MCL1; MDM2; MDM2; MDM2; MDM2; MDM2; MDM2; MDM2; MDM2; MDM2; MDM2; MDM2; MDM2; MDM2; MET; MET; MET; MET; MET; MET; MMP1; MMP1; MMP1; MMP1; MMP1; MMP1; MMP2; MMP2; MMP2; MMP2; MMP2; MMP2; MMP2; MMP9; MMP9; MMP9; MMP9; MMP9; MMP9; MMP9; MMP9; MMP9; MYC; MYC; MYC; MYC; MYC; MYC; MYC; MYC; MYC; MYC; MYC; MYC; MYC; MYC; NFKBIA; NFKBIA; NFKBIA; NFKBIA; NFKBIA; NFKBIA; NFKBIA; NFKBIA; NOS2; NOS2; NOS2; NOS2; NOS2; NOS2; NOS2; NPM1; NPM1; NPM1; NPM1; NR1I2; NR3C1; NR3C1; NR3C1; NR3C1; NR3C1; NR3C1; NR3C1; NR3C1; NR3C2; OPRD1; OPRD1; OPRD1; OPRD1; OPRM1; OPRM1; OPRM1; OPRM1; PCNA; PCNA; PGR; PGR; PGR; PGR; PPARG; PPARG; PPARG; PPARG; PPARG; PPARG; PPARG; PPARG; PTGES; PTGES; PTGS1; PTGS1; PTGS2; PTGS2; PTGS2; PTPN1; PTPN1; RB1; RELA; RELA; RELA RXRA | SLC6A4; TYR; SLC6A3; ADRA2A; APP; ADRA1A; ADRA2C; OPRM1; ADRA1B; F2; ADRA2B; FOS; PTGS2; AKT1; CYP3A4; IL6; DRD1; CHRM1; CASP3; CHRNA7; DRD2; ADRB2; SLC6A2; CHRM3; SLC6A4; SLC6A3; APP; FOS; CHRM1; ADRA1B; F2; EDNRA; EDN3; SLC6A2; CHRM3; SLC6A4; SLC6A3; APP; ADRB2; FOS; CHRM1; F2; EDNRA; EDN3; SLC6A2; OPRD1; SLC6A4; SLC6A3; FOS; ADRA2B; ADRA2C; CHRM2; APP; DRD2; OPRM1; SLC6A2; OPRD1; SLC6A4; APP; FOS; DRD2; ADRA2C; OPRM1; CHRM2; SLC6A2; OPRD1; SLC6A4; SLC6A3; APP; FOS; DRD2; CHRM2; OPRM1; SLC6A2; NR3C1; OPRD1; MDM2; SLC6A4; ICAM1; EGFR; PPARG; INSR; AKT1; IL6; FOS; F2; DRD1; CHRM1; CHRM2; AKT1; AR; HSP90AA1; ICAM1; EGFR; CASP3; PTGES; PTPN1; AKT1; PTGS2; MAPK1; HMOX1; NFKBIA; MMP2; IL2; CCND1; IFNG; MAPK14; IL4; NR3C1; CCNB1; MDM2; TYR; STAT3; ICAM1; RB1; SLC6A3; CCNA2; EGFR; APP; PPARG; NPM1; BIRC5; BCL2L1; INSR; FOS; F2; CASP3; EDNRA; SLC2A4; MMP1; GSK3B; PGR; NOS2; CASP9; HSP90AA1; NR1I2; ESR2; NR3C2; PDE3A; DPP4; TOP1; DRD2; PTGS2; MCL1; CASP7; CD40LG; JUN; PTPN1; MMP9; AR; DRD1; BCL2; RELA; CDKN1A; IL6; OPRM1; ESR1; IL10; RXRA; ITGB3; MYC; MAPK1; HMOX1; MAPK14; OPRD1; CHRM3; EGFR; BCL2L1; PPARG; ECE1; ESR1; IL10; MMP9; MYC; CHRNA7; HSP90AA1; PTGS2; FOS; JUN; GSK3B; CASP3; CHRM2; CHRM1; OPRM1; EDN3; EDNRA; DRD2; RELA; F2; IL6; MAPK1; MMP2; CCND1; MAPK14; NR3C1; CCNB1; MDM2; STAT3; RB1; CCNA2; EGFR; CA2; BCL2L1; INSR; FOS; CASP3; MET; MMP1; GSK3B; PGR; HSP90AA1; CYP3A4; NR3C2; PTGS2; MCL1; CASP7; JUN; MMP9; CYP1A1; CDKN1A; ESR1; RELA; IL6; MYC; MAPK1; CCND1; MAPK14; STAT3; BCL2L1; CASP3; CASP9; HSP90AA1; MCL1; RXRA; ESR1; MYC; MAPK1; HMOX1; NFKBIA; MMP2; IL2; CCND1; IFNG; MAPK14; IL4; NR3C1; CCNB1; MDM2; STAT3; ICAM1; CCNA2; EGFR; BIRC5; TOP1; MET; CD40LG; FOS; ESR1; IL10; HSP90AA1; PTGS2; GSK3B; MMP9; CDKN1A; JUN; PGR; RELA; CASP7; MYC; IL6; CASP3; MCL1; CASP9; CCND1; CCNB1; MDM2; STAT3; CCNA2; EGFR; ESR1; CDKN1A; PCNA; MCL1; HSP90AA1; MYC; CASP7; CASP3; CASP9; MMP9; MAPK1; HMOX1; NFKBIA; MMP2; IL2; CCND1; IFNG; MAPK14; IL4; NR3C1; CCNB1; MDM2; STAT3; ICAM1; RB1; CCNA2; EGFR; PPARG; NPM1; FOS; F2; CD40LG; ESR2; MMP1; PGR; NOS2; RELA; GSK3B; ESR1; IL10; HSP90AA1; PTGS2; MMP9; MET; IL6; MYC; PARP4; JUN; MCL1; CASP7; TOP1; CASP9; CDKN1A; MAPK1; NFKBIA; CCND1; MAPK14; CCNB1; MDM2; STAT3; RB1; CCNA2; EGFR; CASP9; HSP90AA1; MCL1; CDKN1A; MYC; JUN; PARP4; MAPK1; HMOX1; NFKBIA; MMP2; IL2; CCND1; MAPK14; CCNB1; MDM2; STAT3; RB1; CCNA2; EGFR; FOS; GSK3B; RELA; IL10; TOP1; ESR1; PTGS2; MMP9; HSP90AA1; IL6; CDKN1A; MYC; JUN; PARP4; MCL1; MAPK1; IL2; CCND1; CCNB1; MDM2; STAT3; RB1; MMP9; TOP1; NPM1; MCL1; ESR1; FOS; EGFR; PGR; JUN; MYC; PCNA; CDKN1A; MAPK1; MMP2; CCND1; MAPK14; PTGS2; NPM1; PGR; HSP90AA1; MMP9; STAT3; RELA; JUN; ESR1; MCL1; MDM2; FOS; RB1; EGFR; MYC; PCNA; CDKN1A; MAPK1; HMOX1; NFKBIA; MMP2; IL2; IL10; NPM1; NR3C1; ICAM1; MMP1; TP63; HSP90AA1; PPARG; IL4; PTGS2; ESR2; MAPK14; MCL1; MMP9; MET; IL6; MDM2; PGR; EGFR; PCNA; MYC; FOS; RELA; JUN; GSK3B; ESR1; RB1; STAT3; CDKN1A; MAPK1; NFKBIA; IL2; IFNG; MAPK14; IL4; NR3C1; STAT3; ICAM1; F2; PTGS2; MCL1; JUN; CDKN1A; MMP9; IL6; IL10; RELA; MAPK1; HMOX1; MMP2; MAPK14; MDM2; STAT3; TP63; RB1; EGFR; NPM1; FOS; GSK3B; PGR; HSP90AA1; PTGS2; MCL1; JUN; MMP9; PCNA; RELA; IL6; ESR1; MYC; CHRM3; DRD1; RELA; CHRM2; F2; EDNRA; EDN3; JUN; OPRD1; SLC6A4; EGFR; DRD2; OPRM1; CHRNA7; CHRNA7; F2; EDN3; EDNRA; SLC6A4; SLC6A3; DRD2; HMOX1; NR3C1; SLC6A4; PGR; HSP90AA1; NR1I2; CYP3A4; ESR2; PTGS2; JUN; IL6; ESR1; RXRA; NR3C1; KCNH2; EGFR; F2; PGR; NR1I2; IL6; DPP4; RXRA; OPRM1; ESR1; MMP2; IL2; ICAM1; PPARG; SLC2A4; MMP9; IL10; IL6; SLC6A2; SLC6A4; SLC6A3; FOS; DRD2; SLC6A2; NR3C1; OPRD1; SLC6A4; SLC6A3; FOS; OPRM1; OPRD1; EDNRA; EDN3; OPRD1; TYR; FOS; F2; EDNRA; OPRM1; MAPK1; MMP2; EGFR; F2; MCL1; MAPK1; NFKBIA; MMP2; IL2; IFNG; MAPK14; IL4; NR3C1; MDM2; STAT3; TP63; ICAM1; RB1; F2; PCNA; RELA; NPM1; TOP1; MMP1; PTGES; MCL1; ITGB3; MET; ESR2; PPARG; PGR; MMP9; PTGS2; MYC; IL10; JUN; FOS; IL6; ESR1; PTPN1; HSP90AA1; MAPK1; MMP2; MAPK14; NR3C1; MDM2; STAT3; RB1; NPM1; FOS; MET; MMP1; GSK3B; PGR; HSP90AA1; ESR2; TOP1; PTGS2; MCL1; JUN; PTPN1; MMP9; PCNA; RELA; IL6; IL10; MYC; MAPK1; MAPK14; MDM2; STAT3; RB1; FOS; HSP90AA1; IL6; MYC; PTGS2; JUN; F2; NR1I2; F7; MAPK1; HMOX1; IL2; TYR; STAT3; PTGES; IL10; ITGB3; MMP9; PTGS2; F7; IL6; IL6; MAPK1; HMOX1; NFKBIA; MMP2; IL2; IFNG; MAPK14; IL4; NR3C1; OPRD1; MDM2; SLC6A4; STAT3; ICAM1; RB1; SLC6A3; PPARG; MCL1; PGR; MMP9; IL10; OPRM1; PTGS2; MMP1; MYC; RELA; IL6; JUN; MAPK1; NR3C1; MDM2; STAT3; TP63; PPARG; SLC2A4; RELA; IL6; PTGS2; HSP90AA1; PTPN1; MCL1; JUN; MYC; MAPK1; IL2; MYC; HSP90AA1; MMP2; SLC2A4; MMP1; ICAM1; IFNG; STAT3; PPARG; MMP9; NOS2; PTGS2; IL6; IL10; IL4; MAPK14; JUN; MAPK1; MMP2; IL2; IFNG; MAPK14; NR3C1; MDM2; KCNH2; STAT3; RB1; NPM1; MET; PGR; NOS2; IL10; MMP9; PTGS2; RELA; TOP1; JUN; MCL1; IL6; MYC; ITGB3; NR3C2; MAPK1; NFKBIA; MMP2; IL2; IFNG; MAPK14; IL4; STAT3; MYC; ITGB3; NOS2; MMP1; PTGS2; PPARG; RELA; MMP9; JUN; IL6; IL10; MAPK1; NFKBIA; MMP2; IL2; MMP1; MYC; PPARG; PTGS2; MAPK14; MMP9; STAT3; NOS2; PTPN1; IL4; IL6; IL10; JUN; RELA; MAPK1; NFKBIA; MMP2; IL2; MAPK14; IL4; NR3C1; STAT3; PPARG; MMP1; NOS2; PTGES; PTGS1; PTGS2; MCL1; JUN; MMP9; RELA; IL6; MYC; MAPK1; NFKBIA; MMP2; NR3C1; NOS2; TYR; MMP9; PTGS2; MCL1; MYC; MAPK14; IL6; IL4; STAT3; JUN; RELA; MAPK1; MMP2; MAPK14; NR3C1; PPARG; PTPN1; OPRM1; PTGS1; MMP9; NOS2; MMP1; MYC; PTGS2; RELA; JUN; STAT3; IL6; MAPK1; NFKBIA; MMP2; MAPK14; NR3C1; OPRD1; MDM2; SLC6A4; STAT3; PPARG; MET; SLC2A4; MMP1; PGR; NOS2; NR1I2; PTGES; NR3C2; PTGS1; PTGS2; MCL1; JUN; PTPN1; MMP9; RELA; ITGB3; OPRM1; MYC; MAPK1; PPARG; MET; SLC2A4; PTPN1; MAPK1; MMP2; PTGS1; PTPN1; MMP9; MAPK1; NFKBIA; MMP2; MAPK14; NR3C1; MDM2; STAT3; RB1; PPARG; MET; SLC2A4; MMP1; PGR; NOS2; TOP1; PTGS2; MCL1; PTPN1; MMP9; RELA; MYC; SCN5A; SLC2A4; TYR; MDM2; NOS2; PPARG; MMP1; MMP2; PTGS2; PTPN1; MMP9; MCL1; MAPK14; MET; RXRA; RELA; RB1; PGR; NR3C1; NFKBIA; MYC; STAT3; NFKBIA; MMP2; NOS2; MCL1; MDM2; PPARG; MMP1; PTPN1; MMP9; PTGS2; PGR; NR3C1; RB1; RELA; MYC; STAT3; NFKBIA; MMP2; MDM2; STAT3; MET; PTGS2; MMP9; RELA; PCNA; MYC; NR3C1; PGR; PCNA; MET; STAT3; PTGS2; PPARG; TOP1; RELA; NPM1; MYC; RB1; TP63; MMP2; STAT3; PTGS2; MMP9; MYC; PTPN1; MMP2; STAT3; PPARG; MYC; PTGS2; MMP9; PGR; RELA; PPARG; PTGS2; MYC; MMP9; STAT3; NFKBIA; STAT3; PPARG; PGR; NOS2; PTGES; PTGS2; MYC; RELA; NFKBIA; NR3C1; STAT3; TP63; RB1; PPARG; NPM1; SLC2A4; PGR; NOS2; TOP1; PTGS2; RELA; RXRA; NR3C1; PPARG; PTPN1; STAT3; PTGS2; RXRA; NOS2; RELA; STAT3; PPARG; OPRM1; PTGS1; PTGS2; RXRA; RELA; STAT3; RB1; RELA; TOP1; RXRA; PTGS2; PPARG; STAT3; SLC6A4; PGR; RXRA; NR3C2; RELA; PGR; SLC6A4; SLC6A3; STAT3; OPRM1; SLC6A2; SLC6A4; STAT3; SLC6A3; RB1; TOP1; STAT3; RB1; RELA; PTGS2; STAT3; RB1; PTGS1; PTPN1; PTGS2; RELA; SLC2A4; RXRA; PTGS1; PTGS2; TYR; PTGS2; STAT3; RELA; PTPN1; STAT3; SLC2A4; STAT3; STAT3; TP63; RXRA SLC2A4 | 0.433; 0.531; 0.489; 0.424; 0.834; 0.412; 0.416; 0.42; 0.425; 0.429; 0.431; 0.448; 0.451; 0.46; 0.462; 0.503; 0.511; 0.519; 0.533; 0.537; 0.556; 0.563; 0.626; 0.915; 0.557; 0.471; 0.909; 0.477; 0.912; 0.939; 0.905; 0.921; 0.932; 0.597; 0.914; 0.545; 0.427; 0.92; 0.91; 0.489; 0.916; 0.906; 0.919; 0.943; 0.678; 0.928; 0.583; 0.526; 0.475; 0.905; 0.906; 0.912; 0.913; 0.915; 0.927; 0.558; 0.929; 0.503; 0.912; 0.475; 0.916; 0.905; 0.927; 0.912; 0.606; 0.927; 0.523; 0.449; 0.912; 0.477; 0.919; 0.911; 0.927; 0.553; 0.478; 0.518; 0.564; 0.527; 0.529; 0.937; 0.416; 0.505; 0.458; 0.459; 0.473; 0.91; 0.911; 0.913; 0.919; 0.443; 0.474; 0.996; 0.638; 0.426; 0.4; 0.416; 0.439; 0.535; 0.661; 0.905; 0.889; 0.76; 0.836; 0.978; 0.943; 0.53; 0.931; 0.664; 0.728; 0.824; 0.991; 0.485; 0.994; 0.818; 0.955; 0.413; 0.776; 0.678; 0.64; 0.695; 0.827; 0.486; 0.993; 0.948; 0.815; 0.665; 0.969; 0.938; 0.925; 0.547; 0.999; 0.683; 0.909; 0.994; 0.998; 0.435; 0.828; 0.403; 0.556; 0.52; 0.452; 0.616; 0.854; 0.85; 0.672; 0.947; 0.913; 0.987; 0.887; 0.932; 0.466; 0.941; 0.786; 0.995; 0.888; 0.438; 0.991; 0.828; 0.93; 0.96; 0.992; 0.95; 0.42; 0.416; 0.926; 0.909; 0.72; 0.402; 0.416; 0.42; 0.44; 0.478; 0.485; 0.504; 0.557; 0.588; 0.654; 0.698; 0.713; 0.845; 0.891; 0.912; 0.914; 0.916; 0.918; 0.92; 0.931; 0.938; 0.944; 0.957; 0.782; 0.412; 0.986; 0.945; 0.944; 0.413; 0.72; 0.836; 0.963; 0.455; 0.803; 0.5; 0.706; 0.553; 0.647; 0.746; 0.75; 0.509; 0.967; 0.919; 0.988; 0.459; 0.945; 0.442; 0.59; 0.456; 0.736; 0.717; 0.444; 0.51; 0.587; 0.763; 0.787; 0.853; 0.945; 0.433; 0.41; 0.931; 0.991; 0.763; 0.577; 0.437; 0.948; 0.902; 0.925; 0.965; 0.977; 0.559; 0.625; 0.518; 0.63; 0.83; 0.406; 0.798; 0.511; 0.447; 0.668; 0.729; 0.993; 0.478; 0.58; 0.676; 0.543; 0.418; 0.452; 0.534; 0.54; 0.542; 0.557; 0.577; 0.58; 0.602; 0.642; 0.669; 0.681; 0.687; 0.754; 0.796; 0.8; 0.8; 0.937; 0.952; 0.971; 0.583; 0.996; 0.44; 0.962; 0.965; 0.474; 0.44; 0.465; 0.485; 0.505; 0.545; 0.585; 0.65; 0.733; 0.766; 0.414; 0.814; 0.726; 0.626; 0.669; 0.575; 0.854; 0.538; 0.989; 0.544; 0.425; 0.765; 0.902; 0.858; 0.62; 0.967; 0.684; 0.751; 0.578; 0.408; 0.585; 0.451; 0.426; 0.452; 0.453; 0.471; 0.533; 0.557; 0.636; 0.665; 0.669; 0.676; 0.685; 0.723; 0.764; 0.771; 0.775; 0.836; 0.874; 0.912; 0.935; 0.97; 0.972; 0.98; 0.652; 0.454; 0.572; 0.548; 0.551; 0.435; 0.402; 0.503; 0.426; 0.475; 0.948; 0.407; 0.663; 0.44; 0.442; 0.493; 0.826; 0.815; 0.528; 0.52; 0.475; 0.414; 0.737; 0.662; 0.686; 0.629; 0.606; 0.509; 0.581; 0.605; 0.414; 0.625; 0.418; 0.427; 0.428; 0.492; 0.547; 0.554; 0.565; 0.578; 0.634; 0.649; 0.818; 0.825; 0.847; 0.558; 0.542; 0.809; 0.998; 0.96; 0.484; 0.983; 0.42; 0.422; 0.424; 0.51; 0.528; 0.535; 0.562; 0.584; 0.711; 0.718; 0.921; 0.999; 0.597; 0.65; 0.935; 0.413; 0.41; 0.432; 0.451; 0.454; 0.485; 0.528; 0.53; 0.532; 0.534; 0.586; 0.621; 0.662; 0.728; 0.735; 0.8; 0.937; 0.993; 0.887; 0.422; 0.475; 0.709; 0.43; 0.418; 0.427; 0.448; 0.465; 0.476; 0.477; 0.557; 0.605; 0.614; 0.678; 0.709; 0.742; 0.752; 0.765; 0.776; 0.813; 0.821; 0.892; 0.917; 0.959; 0.964; 0.967; 0.967; 0.985; 0.99; 0.99; 0.992; 0.993; 0.999; 0.42; 0.931; 0.883; 0.703; 0.927; 0.914; 0.466; 0.533; 0.816; 0.564; 0.408; 0.42; 0.416; 0.464; 0.522; 0.741; 0.802; 0.945; 0.538; 0.439; 0.429; 0.646; 0.939; 0.976; 0.727; 0.981; 0.581; 0.581; 0.518; 0.599; 0.56; 0.961; 0.471; 0.548; 0.981; 0.47; 0.999; 0.585; 0.686; 0.714; 0.996; 0.911; 0.42; 0.641; 0.813; 0.9; 0.9; 0.904; 0.907; 0.918; 0.439; 0.905; 0.919; 0.918; 0.479; 0.433; 0.9; 0.9; 0.907; 0.479; 0.44; 0.547; 0.547; 0.459; 0.5; 0.404; 0.608; 0.699; 0.95; 0.553; 0.502; 0.406; 0.477; 0.696; 0.922; 0.567; 0.561; 0.45; 0.519; 0.419; 0.962; 0.42; 0.447; 0.459; 0.476; 0.548; 0.404; 0.499; 0.441; 0.51; 0.461; 0.405; 0.41; 0.514; 0.644; 0.745; 0.892; 0.793; 0.494; 0.733; 0.448; 0.93; 0.847; 0.99; 0.669; 0.932; 0.403; 0.731; 0.732; 0.473; 0.546; 0.466; 0.918; 0.99; 0.483; 0.529; 0.412; 0.939; 0.905; 0.561; 0.965; 0.475; 0.697; 0.811; 0.766; 0.81; 0.778; 0.643; 0.862; 0.998; 0.502; 0.539; 0.52; 0.413; 0.424; 0.473; 0.521; 0.531; 0.541; 0.566; 0.636; 0.699; 0.715; 0.749; 0.787; 0.811; 0.828; 0.872; 0.872; 0.88; 0.88; 0.9; 0.921; 0.943; 0.988; 0.989; 0.971; 0.532; 0.962; 0.925; 0.723; 0.855; 0.733; 0.412; 0.982; 0.473; 0.633; 0.409; 0.964; 0.979; 0.941; 0.411; 0.773; 0.515; 0.979; 0.484; 0.581; 0.92; 0.666; 0.769; 0.422; 0.983; 0.915; 0.832; 0.485; 0.578; 0.518; 0.953; 0.959; 0.42; 0.429; 0.444; 0.882; 0.938; 0.569; 0.964; 0.931; 0.508; 0.406; 0.665; 0.443; 0.448; 0.451; 0.463; 0.481; 0.511; 0.518; 0.724; 0.473; 0.992; 0.946; 0.563; 0.521; 0.822; 0.445; 0.978; 0.726; 0.972; 0.582; 0.462; 0.485; 0.988; 0.671; 0.577; 0.48; 0.968; 0.471; 0.514; 0.652; 0.748; 0.792; 0.817; 0.821; 0.915; 0.968; 0.982; 0.999; 0.657; 0.943; 0.666; 0.684; 0.906; 0.578; 0.488; 0.443; 0.573; 0.637; 0.674; 0.697; 0.753; 0.978; 0.982; 0.779; 0.42; 0.42; 0.44; 0.449; 0.561; 0.595; 0.653; 0.67; 0.714; 0.735; 0.759; 0.821; 0.834; 0.847; 0.868; 0.945; 0.972; 0.977; 0.979; 0.921; 0.939; 0.922; 0.589; 0.983; 0.706; 0.766; 0.982; 0.932; 0.587; 0.704; 0.985; 0.708; 0.4; 0.407; 0.409; 0.461; 0.503; 0.549; 0.557; 0.709; 0.762; 0.907; 0.967; 0.723; 0.789; 0.743; 0.834; 0.87; 0.655; 0.862; 0.975; 0.463; 0.535; 0.554; 0.596; 0.705; 0.705; 0.735; 0.737; 0.755; 0.953; 0.985; 0.462; 0.434; 0.638; 0.938; 0.408; 0.448; 0.449; 0.594; 0.603; 0.735; 0.779; 0.836; 0.907; 0.921; 0.939; 0.946; 0.949; 0.959; 0.777; 0.521; 0.584; 0.957; 0.754; 0.972; 0.77; 0.995; 0.556; 0.555; 0.696; 0.423; 0.425; 0.836; 0.4; 0.76; 0.831; 0.547; 0.997; 0.491; 0.954; 0.429; 0.44; 0.473; 0.486; 0.53; 0.556; 0.577; 0.668; 0.792; 0.941; 0.959; 0.964; 0.969; 0.971; 0.978; 0.55; 0.473; 0.962; 0.473; 0.52; 0.529; 0.586; 0.596; 0.648; 0.66; 0.685; 0.71; 0.8; 0.942; 0.954; 0.98; 0.996; 0.982; 0.693; 0.823; 0.835; 0.646; 0.416; 0.41; 0.418; 0.997; 0.812; 0.515; 0.766; 0.836; 0.442; 0.741; 0.47; 0.546; 0.418; 0.556; 0.945; 0.825; 0.984; 0.661; 0.923; 0.983; 0.462; 0.655; 0.85; 0.488; 0.466; 0.473; 0.684; 0.998; 0.926; 0.537; 0.518; 0.941; 0.44; 0.996; 0.968; 0.64; 0.995; 0.985; 0.593; 0.921; 0.96; 0.956; 0.52; 0.42; 0.767; 0.469; 0.956; 0.564; 0.711; 0.609; 0.44; 0.893; 0.98; 0.992; 0.933; 0.402; 0.432; 0.488; 0.521; 0.672; 0.717; 0.746; 0.753; 0.76; 0.799; 0.82; 0.889; 0.929; 0.941; 0.952; 0.954; 0.957; 0.961; 0.961; 0.986; 0.989; 0.977; 0.659; 0.505; 0.558; 0.563; 0.588; 0.622; 0.652; 0.698; 0.785; 0.844; 0.934; 0.943; 0.948; 0.949; 0.971; 0.459; 0.41; 0.596; 0.985; 0.445; 0.662; 0.447; 0.449; 0.485; 0.858; 0.602; 0.411; 0.422; 0.423; 0.495; 0.51; 0.53; 0.636; 0.639; 0.737; 0.769; 0.971; 0.989; 0.523; 0.978; 0.422; 0.546; 0.636; 0.966; 0.922; 0.966; 0.567; 0.408; 0.696; 0.961; 0.42; 0.59; 0.658; 0.704; 0.734; 0.908; 0.979; 0.55; 0.981; 0.709; 0.473; 0.53; 0.42; 0.741; 0.664; 0.783; 0.943; 0.514; 0.99; 0.496; 0.761; 0.496; 0.605; 0.498; 0.701; 0.918; 0.725; 0.57; 0.972; 0.926; 0.416; 0.439; 0.464; 0.482; 0.801; 0.915; 0.953; 0.998; 0.955; 0.459; 0.448; 0.454; 0.906; 0.909; 0.951; 0.478; 0.686; 0.438; 0.55; 0.917; 0.495; 0.539; 0.668; 0.672; 0.912; 0.923; 0.943; 0.963; 0.914; 0.553; 0.567; 0.912; 0.945; 0.437; 0.679; 0.918; 0.679; 0.928; 0.417; 0.684; 0.704; 0.505; 0.585; 0.535; 0.941; 0.423; 0.46; 0.819; 0.962; 0.973; 0.997; 0.97; 0.984; 0.406; 0.889; 0.98; 0.558; 0.573; 0.859; 0.59; 0.578; 0.876; 0.49; 0.628 0.918 |
