# Supplementary material for: Molecular Mechanism of Salvia miltiorrhiza Bunge in Treating Cerebral Infarction
Source: Evid Based Complement Alternat Med. 2022 Mar 29;2022:5992394. doi: 10.1155/2022/5992394 (PMC8983215; doi:10.1155/2022/5992394)
Supplement: Supplementary Materials — Table S1: details of active compounds and targets. Table S2: known therapeutic targets in cerebral infarction. Table S3: the details of the PPI network. Table S4: the details of the GO analysis. Table S5: the KEGG pathway enrichment analysis. [file 5992394.f1.zip › 5992394.f1/Table S4-The details of the GO analysis (1).doc]

**Table S6: The details of the GO analysis**

| **ONTOLOGY** | **Description** | **p.adjust** |
| --- | --- | --- |
| BP; BP; BP; BP; BP; BP; BP; BP; BP; BP; BP; BP; BP; BP; BP; BP; BP; BP; BP; BP; BP; BP; BP; BP; BP; BP; BP; BP; BP; BP; BP; BP; BP; BP; BP; BP; BP; BP; BP; BP; BP; BP; BP; BP; BP; BP; BP; BP; BP; BP; BP; BP; BP; BP; BP; BP; BP; BP; BP; BP; BP; BP; BP; BP; BP; BP; BP; BP; BP; BP; BP; BP; BP; BP; BP; BP; BP; BP; BP; BP; BP; BP; BP; BP; BP; BP; BP; BP; BP; BP; BP; BP; BP; BP; BP; BP; BP; BP; BP; BP; BP; BP; BP; BP; BP; BP; BP; BP; BP; BP; BP; BP; BP; BP; BP; BP; BP; BP; BP; BP; BP; BP; BP; BP; BP; BP; BP; BP; BP; BP; BP; BP; BP; BP; BP; BP; BP; BP; BP; BP; BP; BP; BP; BP; BP; BP; BP; BP; BP; BP; BP; BP; BP; BP; BP; BP; BP; BP; BP; BP; BP; BP; BP; BP; BP; BP; BP; BP; BP; BP; BP; BP; BP; BP; BP; BP; BP; BP; BP; BP; BP; BP; BP; BP; BP; BP; BP; BP; BP; BP; BP; BP; BP; BP; BP; BP; BP; BP; BP; BP; BP; BP; BP; BP; BP; BP; BP; BP; BP; BP; BP; BP; BP; BP; BP; BP; BP; BP; BP; BP; BP; BP; BP; BP; BP; BP; BP; BP; BP; BP; BP; BP; BP; BP; BP; BP; BP; BP; BP; BP; BP; BP; BP; BP; BP; BP; BP; BP; BP; BP; BP; BP; BP; BP; BP; BP; BP; BP; BP; BP; BP; BP; BP; BP; BP; BP; BP; BP; BP; BP; BP; BP; BP; BP; BP; BP; BP; BP; BP; BP; BP; BP; BP; BP; BP; BP; BP; BP; BP; BP; BP; BP; BP; BP; BP; BP; BP; BP; BP; BP; BP; BP; BP; BP; BP; BP; BP; BP; BP; BP; BP; BP; BP; BP; BP; BP; BP; BP; BP; BP; BP; BP; BP; BP; BP; BP; BP; BP; BP; BP; BP; BP; BP; BP; BP; BP; BP; BP; BP; BP; BP; BP; BP; BP; BP; BP; BP; BP; BP; BP; BP; BP; BP; BP; BP; BP; BP; BP; BP; BP; BP; BP; BP; BP; BP; BP; BP; BP; BP; BP; BP; BP; BP; BP; BP; BP; BP; BP; BP; BP; BP; BP; BP; BP; BP; BP; BP; BP; BP; BP; BP; BP; BP; BP; BP; BP; BP; BP; BP; BP; BP; BP; BP; BP; BP; BP; BP; BP; BP; BP; BP; BP; BP; BP; BP; BP; BP; BP; BP; BP; BP; BP; BP; BP; BP; BP; BP; BP; BP; BP; BP; BP; BP; BP; BP; BP; BP; BP; BP; BP; BP; BP; BP; BP; BP; BP; BP; BP; BP; BP; BP; BP; BP; BP; BP; BP; BP; BP; BP; BP; BP; BP; BP; BP; BP; BP; BP; BP; BP; BP; BP; BP; BP; BP; BP; BP; BP; BP; BP; BP; BP; BP; BP; BP; BP; BP; BP; BP; BP; BP; BP; BP; BP; BP; BP; BP; BP; BP; BP; BP; BP; BP; BP; BP; BP; BP; BP; BP; BP; BP; BP; BP; BP; BP; BP; BP; BP; BP; BP; BP; BP; BP; BP; BP; BP; BP; BP; BP; BP; BP; BP; BP; BP; BP; BP; BP; BP; BP; BP; BP; BP; BP; BP; BP; BP; BP; BP; BP; BP; BP; BP; BP; BP; BP; BP; BP; BP; BP; BP; BP; BP; BP; BP; BP; BP; BP; BP; BP; BP; BP; BP; BP; BP; BP; BP; BP; BP; BP; BP; BP; BP; BP; BP; BP; BP; BP; BP; BP; BP; BP; BP; BP; BP; BP; BP; BP; BP; BP; BP; BP; BP; BP; BP; BP; BP; BP; BP; BP; BP; BP; BP; BP; BP; BP; BP; BP; BP; BP; BP; BP; BP; BP; BP; BP; BP; BP; BP; BP; BP; BP; BP; BP; BP; BP; BP; BP; BP; BP; BP; BP; BP; BP; BP; BP; BP; BP; BP; BP; BP; BP; BP; BP; BP; BP; BP; BP; BP; BP; BP; BP; BP; BP; BP; BP; BP; BP; BP; BP; BP; BP; BP; BP; BP; BP; BP; BP; BP; BP; BP; BP; BP; BP; BP; BP; BP; BP; BP; BP; BP; BP; BP; BP; BP; BP; BP; BP; BP; BP; BP; BP; BP; BP; BP; BP; BP; BP; BP; BP; BP; BP; BP; BP; BP; BP; BP; BP; BP; BP; BP; BP; BP; BP; BP; BP; BP; BP; BP; BP; BP; BP; BP; BP; BP; BP; BP; BP; BP; BP; BP; BP; BP; BP; BP; BP; BP; BP; BP; BP; BP; BP; BP; BP; BP; BP; BP; BP; BP; BP; BP; BP; BP; BP; BP; BP; BP; BP; BP; BP; BP; BP; BP; BP; BP; BP; BP; BP; BP; BP; BP; BP; BP; BP; BP; BP; BP; BP; BP; BP; BP; BP; BP; BP; BP; BP; BP; BP; BP; BP; BP; BP; BP; BP; BP; BP; BP; BP; BP; BP; BP; BP; BP; BP; BP; BP; BP; BP; BP; BP; BP; BP; BP; BP; BP; BP; BP; BP; BP; BP; BP; BP; BP; BP; BP; BP; BP; BP; BP; BP; BP; BP; BP; BP; BP; BP; BP; BP; BP; BP; BP; BP; BP; BP; BP; BP; BP; BP; BP; BP; BP; BP; BP; BP; BP; BP; BP; BP; BP; BP; BP; BP; BP; BP; BP; BP; BP; BP; BP; BP; BP; BP; BP; BP; BP; BP; BP; BP; BP; BP; BP; BP; BP; BP; BP; BP; BP; BP; BP; BP; BP; BP; BP; BP; BP; BP; BP; BP; BP; BP; BP; BP; BP; BP; BP; BP; BP; BP; BP; BP; BP; BP; BP; BP; BP; BP; BP; BP; BP; BP; BP; BP; BP; BP; BP; BP; BP; BP; BP; BP; BP; BP; BP; BP; BP; BP; BP; BP; BP; BP; BP; BP; BP; BP; BP; BP; BP; BP; BP; BP; BP; BP; BP; BP; BP; BP; BP; BP; BP; BP; BP; BP; BP; BP; BP; BP; BP; BP; BP; BP; BP; BP; BP; BP; BP; BP; BP; BP; BP; BP; BP; BP; BP; BP; BP; BP; BP; BP; BP; BP; BP; BP; BP; BP; BP; BP; BP; BP; BP; BP; BP; BP; BP; BP; BP; BP; BP; BP; BP; BP; BP; BP; BP; BP; BP; BP; BP; BP; BP; BP; BP; BP; BP; BP; BP; BP; BP; BP; BP; BP; BP; BP; BP; BP; BP; BP; BP; BP; BP; BP; BP; BP; BP; BP; BP; BP; BP; BP; BP; BP; BP; BP; BP; BP; BP; BP; BP; BP; BP; BP; BP; BP; BP; BP; BP; BP; BP; BP; BP; BP; BP; BP; BP; BP; BP; BP; BP; BP; BP; BP; BP; BP; BP; BP; BP; BP; BP; BP; BP; BP; BP; BP; BP; BP; BP; BP; BP; BP; BP; BP; BP; BP; BP; BP; BP; BP; BP; BP; BP; BP; BP; BP; BP; BP; BP; BP; BP; BP; BP; BP; BP; BP; BP; BP; BP; BP; BP; BP; BP; BP; BP; BP; BP; BP; BP; BP; BP; BP; BP; BP; BP; BP; BP; BP; BP; BP; BP; BP; BP; BP; BP; BP; BP; BP; BP; BP; BP; BP; BP; BP; BP; BP; BP; BP; BP; BP; BP; BP; BP; BP; BP; BP; BP; BP; BP; BP; BP; BP; BP; BP; BP; BP; BP; BP; BP; BP; BP; BP; BP; BP; BP; BP; BP; BP; BP; BP; BP; BP; BP; BP; BP; BP; BP; BP; BP; BP; BP; BP; BP; BP; BP; BP; BP; BP; BP; BP; BP; BP; BP; BP; BP; BP; BP; BP; BP; BP; BP; BP; BP; BP; BP; BP; BP; BP; BP; BP; BP; BP; BP; BP; BP; BP; BP; BP; BP; BP; BP; BP; BP; BP; BP; BP; BP; BP; BP; BP; BP; BP; BP; BP; BP; BP; BP; BP; BP; BP; BP; BP; BP; BP; BP; BP; BP; BP; BP; BP; BP; BP; BP; BP; BP; BP; BP; BP; BP; BP; BP; BP; BP; BP; BP; BP; BP; BP; BP; BP; BP; BP; BP; BP; BP; BP; BP; BP; BP; BP; BP; BP; BP; BP; BP; BP; BP; BP; BP; BP; BP; BP; BP; BP; BP; BP; BP; BP; BP; BP; BP; BP; BP; BP; BP; BP; BP; BP; BP; BP; BP; BP; BP; BP; BP; BP; BP; BP; BP; BP; BP; BP; BP; BP; BP; BP; BP; BP; BP; BP; BP; BP; BP; BP; BP; BP; BP; BP; BP; BP; BP; BP; BP; BP; BP; BP; BP; BP; BP; BP; BP; BP; BP; BP; BP; BP; BP; BP; BP; BP; BP; BP; BP; BP; BP; BP; BP; BP; BP; BP; BP; BP; BP; BP; BP; BP; BP; BP; BP; BP; BP; BP; BP; BP; BP; BP; BP; BP; BP; BP; BP; BP; BP; BP; BP; BP; BP; BP; BP; BP; BP; BP; BP; BP; BP; BP; BP; BP; BP; BP; BP; BP; BP; BP; BP; BP; BP; BP; BP; BP; BP; BP; BP; BP; BP; BP; BP; BP; BP; BP; BP; BP; BP; BP; BP; BP; BP; BP; BP; BP; BP; BP; BP; BP; BP; BP; BP; BP; BP; BP; BP; BP; BP; BP; BP; BP; BP; BP; BP; BP; BP; BP; BP; BP; BP; BP; BP; BP; BP; BP; BP; BP; BP; BP; BP; BP; BP; BP; BP; BP; BP; BP; BP; BP; BP; BP; BP; BP; BP; BP; BP; BP; BP; BP; BP; BP; BP; BP; BP; BP; BP; BP; BP; BP; BP; BP; BP; BP; BP; BP; BP; BP; BP; BP; BP; BP; BP; BP; BP; BP; BP; BP; BP; BP; BP; BP; BP; BP; BP; BP; BP; BP; BP; BP; BP; BP; BP; BP; BP; BP; BP; BP; BP; BP; BP; BP; BP; BP; BP; BP; BP; BP; BP; BP; BP; BP; BP; BP; BP; BP; BP; BP; BP; BP; BP; BP; BP; BP; BP; BP; BP; BP; BP; BP; BP; BP; BP; BP; BP; BP; BP; BP; BP; BP; BP; BP; BP; BP; BP; BP; BP; BP; BP; BP; BP; BP; BP; BP; BP; BP; BP; BP; BP; BP; BP; BP; BP; BP; BP; BP; BP; BP; BP; BP; BP; BP; BP; BP; BP; BP; BP; BP; BP; BP; BP; BP; BP; BP; BP; BP; BP; BP; BP; BP; BP; BP; BP; BP; BP; BP; BP; BP; BP; BP; BP; BP; BP; BP; BP; BP; BP; BP; BP; BP; BP; CC; CC; CC; CC; CC; CC; CC; CC; CC; CC; CC; CC; CC; CC; CC; CC; CC; CC; CC; CC; CC; CC; CC; CC; CC; CC; CC; CC; CC; CC; CC; CC; CC; CC; CC; CC; CC; CC; CC; CC; CC; CC; CC; CC; CC; CC; CC; CC; CC; CC; CC; CC; CC; CC; CC; CC; CC; CC; CC; CC; CC; CC; CC; CC; CC; CC; CC; CC; CC; CC; CC; CC; CC; CC; CC; CC; CC; CC; CC; CC; CC; MF; MF; MF; MF; MF; MF; MF; MF; MF; MF; MF; MF; MF; MF; MF; MF; MF; MF; MF; MF; MF; MF; MF; MF; MF; MF; MF; MF; MF; MF; MF; MF; MF; MF; MF; MF; MF; MF; MF; MF; MF; MF; MF; MF; MF; MF; MF; MF; MF; MF; MF; MF; MF; MF; MF; MF; MF; MF; MF; MF; MF; MF; MF; MF; MF; MF; MF; MF; MF; MF; MF; MF; MF; MF; MF; MF; MF; MF; MF; MF; MF; MF; MF; MF; MF; MF; MF; MF; MF; MF; MF; MF; MF; MF; MF; MF; MF; MF; MF; MF; MF; MF; MF; MF; MF; MF; MF; MF; MF MF | response to steroid hormone; vascular process in circulatory system; cellular response to drug; response to metal ion; regulation of tube diameter; regulation of blood vessel diameter; response to antibiotic; regulation of tube size; vasoconstriction; response to oxygen levels; cellular response to chemical stress; negative regulation of blood vessel diameter; muscle cell proliferation; response to radiation; response to hypoxia; regulation of vasoconstriction; response to decreased oxygen levels; response to xenobiotic stimulus; aging; response to reactive oxygen species; regulation of DNA-binding transcription factor activity; gland development; response to oxidative stress; regulation of blood circulation; smooth muscle contraction; response to UV; multi-multicellular organism process; response to light stimulus; response to ketone; cellular response to reactive oxygen species; regulation of smooth muscle cell proliferation; cellular response to oxidative stress; smooth muscle cell proliferation; regulation of neurotransmitter levels; regulation of blood pressure; response to alkaloid; response to glucocorticoid; response to nutrient levels; response to lipopolysaccharide; female pregnancy; response to molecule of bacterial origin; neuron death; response to corticosteroid; phospholipase C-activating G protein-coupled receptor signaling pathway; positive regulation of vasoconstriction; positive regulation of smooth muscle cell proliferation; response to estradiol; response to iron ion; reproductive structure development; reproductive system development; adenylate cyclase-modulating G protein-coupled receptor signaling pathway; neurotransmitter biosynthetic process; response to alcohol; reactive oxygen species metabolic process; response to acid chemical; regulation of reactive oxygen species metabolic process; peptidyl-serine phosphorylation; cellular response to steroid hormone stimulus; regulation of smooth muscle contraction; G protein-coupled receptor signaling pathway, coupled to cyclic nucleotide second messenger; positive regulation of DNA-binding transcription factor activity; epithelial cell proliferation; cellular response to inorganic substance; peptidyl-serine modification; positive regulation of reactive oxygen species metabolic process; extrinsic apoptotic signaling pathway; positive regulation of cell cycle; animal organ regeneration; positive regulation of blood circulation; cellular response to xenobiotic stimulus; activation of protein kinase activity; response to nicotine; positive regulation of protein serine/threonine kinase activity; negative regulation of apoptotic signaling pathway; adenylate cyclase-activating adrenergic receptor signaling pathway; transcription initiation from RNA polymerase II promoter; cellular response to metal ion; regulation of developmental growth; rhythmic process; positive regulation of cell cycle process; neurotransmitter metabolic process; adrenergic receptor signaling pathway; regeneration; DNA-templated transcription, initiation; muscle contraction; cellular response to alkaloid; regulation of membrane potential; positive regulation of epithelial cell proliferation; regulation of neuron death; reactive oxygen species biosynthetic process; regulation of inflammatory response; cellular response to cadmium ion; postsynaptic signal transduction; regulation of epithelial cell proliferation; monoamine transport; positive regulation of synaptic transmission; cellular response to peptide; response to cadmium ion; response to vitamin; response to nutrient; maternal process involved in female pregnancy; cellular response to abiotic stimulus; cellular response to environmental stimulus; intracellular receptor signaling pathway; muscle system process; regulation of apoptotic signaling pathway; steroid hormone mediated signaling pathway; cellular response to oxygen levels; intrinsic apoptotic signaling pathway; regulation of DNA metabolic process; myeloid cell differentiation; multicellular organismal homeostasis; signal transduction in absence of ligand; extrinsic apoptotic signaling pathway in absence of ligand; positive regulation of peptidyl-serine phosphorylation; cognition; positive regulation of cell cycle phase transition; response to estrogen; response to hydrogen peroxide; pri-miRNA transcription by RNA polymerase II; peptidyl-tyrosine phosphorylation; neuroinflammatory response; response to purine-containing compound; cellular response to monoamine stimulus; cellular response to catecholamine stimulus; peptidyl-tyrosine modification; morphogenesis of a branching structure; response to peptide hormone; modulation of chemical synaptic transmission; regulation of trans-synaptic signaling; negative regulation of catabolic process; nitric oxide biosynthetic process; positive regulation of protein transport; regulation of binding; response to anesthetic; response to monoamine; response to catecholamine; learning or memory; regulation of peptidyl-tyrosine phosphorylation; positive regulation of fibroblast proliferation; myeloid leukocyte differentiation; astrocyte differentiation; cellular response to hypoxia; organic hydroxy compound transport; protein localization to nucleus; nitric oxide metabolic process; positive regulation of establishment of protein localization; response to mechanical stimulus; regulation of fibroblast proliferation; positive regulation of pri-miRNA transcription by RNA polymerase II; positive regulation of mitotic cell cycle; response to amyloid-beta; negative regulation of autophagy; fibroblast proliferation; regulation of autophagy; regulation of metal ion transport; regulation of vascular smooth muscle cell proliferation; vascular smooth muscle cell proliferation; reactive nitrogen species metabolic process; cellular response to decreased oxygen levels; glial cell differentiation; positive regulation of ion transport; regulation of muscle contraction; adenylate cyclase-inhibiting G protein-coupled receptor signaling pathway; positive regulation of receptor signaling pathway via JAK-STAT; positive regulation of mitotic cell cycle phase transition; positive regulation of receptor signaling pathway via STAT; cellular response to ketone; cellular response to dopamine; response to muscle stretch; response to dopamine; gliogenesis; response to organophosphorus; hormone-mediated signaling pathway; autophagy; process utilizing autophagic mechanism; liver development; morphogenesis of a branching epithelium; cAMP-mediated signaling; regulation of G1/S transition of mitotic cell cycle; hepaticobiliary system development; positive regulation of cellular catabolic process; cell cycle G1/S phase transition; negative regulation of intrinsic apoptotic signaling pathway; adenylate cyclase-activating G protein-coupled receptor signaling pathway; regulation of peptidyl-serine phosphorylation; positive regulation of small molecule metabolic process; response to temperature stimulus; regulation of nitric oxide biosynthetic process; second-messenger-mediated signaling; anatomical structure homeostasis; regulation of reactive oxygen species biosynthetic process; protein import into nucleus; protein import; positive regulation of peptidyl-tyrosine phosphorylation; negative regulation of extrinsic apoptotic signaling pathway; regulation of pri-miRNA transcription by RNA polymerase II; response to ionizing radiation; acute inflammatory response; regulation of cell cycle G1/S phase transition; positive regulation of nitric oxide biosynthetic process; positive regulation of transmembrane transport; organ growth; positive regulation of cytokine production; positive regulation of cellular protein localization; positive regulation of nitric oxide metabolic process; epithelial cell apoptotic process; regulation of generation of precursor metabolites and energy; regulation of protein secretion; response to amino acid; regulation of cytokine biosynthetic process; neurotransmitter uptake; positive regulation of nucleotide metabolic process; positive regulation of purine nucleotide metabolic process; positive regulation of growth; cyclic-nucleotide-mediated signaling; female sex differentiation; catecholamine transport; positive regulation of ATP metabolic process; cellular response to external stimulus; import into nucleus; regulation of cation transmembrane transport; nucleocytoplasmic transport; G1/S transition of mitotic cell cycle; nuclear transport; receptor biosynthetic process; regulation of signaling receptor activity; cellular response to UV; cytokine biosynthetic process; regulation of peptide secretion; positive regulation of catabolic process; cytokine metabolic process; neurotransmitter reuptake; response to ethanol; positive regulation of intracellular protein transport; acetylcholine receptor signaling pathway; cellular response to biotic stimulus; response to cocaine; neuron apoptotic process; regulation of leukocyte cell-cell adhesion; liver regeneration; response to acetylcholine; cellular response to acetylcholine; positive regulation of myeloid cell differentiation; positive regulation of hemopoiesis; positive regulation of reactive oxygen species biosynthetic process; regulation of systemic arterial blood pressure; synaptic transmission, dopaminergic; regulation of receptor signaling pathway via JAK-STAT; cellular response to toxic substance; positive regulation of neuron death; B cell proliferation; positive regulation of DNA metabolic process; cellular response to radiation; import into cell; regulation of intracellular protein transport; positive regulation of smooth muscle contraction; regulation of postsynaptic membrane potential; female gonad development; response to antineoplastic agent; regulation of myeloid cell differentiation; ERK1 and ERK2 cascade; regulation of B cell proliferation; negative regulation of phosphorylation; positive regulation of cell-cell adhesion; cellular response to peptide hormone stimulus; positive regulation of nucleocytoplasmic transport; positive regulation of MAP kinase activity; excitatory postsynaptic potential; activation of protein kinase B activity; positive regulation of signaling receptor activity; regulation of muscle system process; digestive system development; regulation of receptor signaling pathway via STAT; feeding behavior; development of primary female sexual characteristics; negative regulation of catecholamine secretion; regulation of chemokine biosynthetic process; dopamine uptake; regulation of cyclin-dependent protein serine/threonine kinase activity; regulation of cell-cell adhesion; regulation of cell cycle phase transition; positive regulation of cyclin-dependent protein kinase activity; regulation of ion transmembrane transport; cell growth; cellular response to lipopolysaccharide; intrinsic apoptotic signaling pathway in response to DNA damage; cellular response to extracellular stimulus; regulation of multicellular organism growth; placenta development; positive regulation of cellular amide metabolic process; leukocyte cell-cell adhesion; platelet activation; circadian rhythm; regulation of synaptic transmission, dopaminergic; chemokine biosynthetic process; chemokine metabolic process; catecholamine uptake; positive regulation of amyloid-beta formation; sex differentiation; regulation of cyclin-dependent protein kinase activity; positive regulation of cytokine biosynthetic process; regulation of extrinsic apoptotic signaling pathway; chemical synaptic transmission, postsynaptic; cellular response to molecule of bacterial origin; negative regulation of ion transport; ovulation cycle; regulation of cysteine-type endopeptidase activity involved in apoptotic process; receptor signaling pathway via JAK-STAT; gonad development; positive regulation of leukocyte cell-cell adhesion; epithelial cell migration; negative regulation of protein phosphorylation; mammary gland epithelium development; response to dexamethasone; epithelium migration; regulation of intrinsic apoptotic signaling pathway; positive regulation of proteolysis; development of primary sexual characteristics; positive regulation of protein localization to nucleus; regulation of protein localization to nucleus; tissue migration; regulation of myeloid leukocyte differentiation; regulation of epithelial cell migration; negative regulation of binding; receptor signaling pathway via STAT; G protein-coupled acetylcholine receptor signaling pathway; negative regulation of lipid storage; regulation of mitotic cell cycle phase transition; tissue homeostasis; positive regulation of intracellular transport; positive regulation of epithelial cell migration; gland morphogenesis; regulation of intracellular transport; anatomical structure maturation; temperature homeostasis; astrocyte development; positive regulation of amyloid precursor protein catabolic process; regulation of peptidase activity; apoptotic mitochondrial changes; regulation of DNA binding; cellular response to nutrient levels; cell maturation; cellular calcium ion homeostasis; negative regulation of G1/S transition of mitotic cell cycle; regulation of cysteine-type endopeptidase activity; tissue remodeling; norepinephrine transport; ameboidal-type cell migration; endocrine system development; T cell activation; lymphocyte activation involved in immune response; response to tumor necrosis factor; regulation of protein kinase B signaling; cellular response to light stimulus; positive regulation of developmental growth; homeostasis of number of cells; intracellular steroid hormone receptor signaling pathway; positive regulation of muscle contraction; regulation of telomerase activity; calcium ion homeostasis; negative regulation of cell cycle G1/S phase transition; regulation of systemic arterial blood pressure mediated by a chemical signal; negative regulation of epithelial cell differentiation; positive regulation of vascular smooth muscle cell proliferation; long-term synaptic potentiation; peptide hormone secretion; regulation of heart contraction; ossification; digestive tract development; response to cold; positive regulation of G1/S transition of mitotic cell cycle; negative regulation of cellular catabolic process; receptor metabolic process; positive regulation of cell adhesion; regulation of nervous system process; male gonad development; urogenital system development; regulation of production of molecular mediator of immune response; development of primary male sexual characteristics; regulation of neurotransmitter transport; regulation of leukocyte migration; cellular divalent inorganic cation homeostasis; vascular smooth muscle contraction; negative regulation of amine transport; blood coagulation; ERBB signaling pathway; positive regulation of myeloid leukocyte differentiation; regulation of interleukin-12 production; regulation of MAP kinase activity; leukocyte migration; negative regulation of mitotic cell cycle; mammary gland development; positive regulation of leukocyte differentiation; interaction with host; prostate gland epithelium morphogenesis; hemostasis; protein kinase B signaling; learning; coagulation; neurotransmitter transport; multicellular organism growth; response to gamma radiation; interleukin-12 production; response to insulin; regulation of endopeptidase activity; response to cAMP; response to immobilization stress; astrocyte activation; epithelial cell development; regulation of lymphocyte proliferation; negative regulation of neuron death; positive regulation of NF-kappaB transcription factor activity; regulation of mononuclear cell proliferation; cellular response to hydrogen peroxide; branching morphogenesis of an epithelial tube; glial cell activation; prostate gland morphogenesis; regulation of amyloid-beta formation; calcium ion transport; activation of MAPK activity; release of cytochrome c from mitochondria; regulation of heart rate; heart contraction; regulation of response to DNA damage stimulus; positive regulation of cell cycle G1/S phase transition; regulation of transporter activity; positive regulation of ERK1 and ERK2 cascade; developmental maturation; cell cycle checkpoint; phenol-containing compound metabolic process; negative regulation of transferase activity; regulation of exocytosis; regulation of protein binding; negative regulation of lipid localization; sensory perception of pain; regulation of nucleocytoplasmic transport; leukocyte apoptotic process; regulation of deoxyribonuclease activity; positive regulation of MHC class II biosynthetic process; cellular response to dexamethasone stimulus; positive regulation of cell cycle G2/M phase transition; bone resorption; regulation of immunoglobulin production; mitotic G1 DNA damage checkpoint; amyloid precursor protein metabolic process; mitotic G1/S transition checkpoint; regulation of leukocyte proliferation; male sex differentiation; regulation of regulated secretory pathway; heart process; fat cell differentiation; regulation of cellular respiration; positive regulation of cyclin-dependent protein serine/threonine kinase activity; G1 DNA damage checkpoint; glucose transmembrane transport; regulation of DNA biosynthetic process; cyclooxygenase pathway; dopamine uptake involved in synaptic transmission; catecholamine uptake involved in synaptic transmission; prostate glandular acinus development; mitotic cell cycle checkpoint; negative regulation of calcium ion transport; regulation of ERK1 and ERK2 cascade; regulation of immune effector process; positive regulation of leukocyte activation; positive regulation of endothelial cell proliferation; hexose transmembrane transport; regulation of synaptic vesicle cycle; negative regulation of smooth muscle cell proliferation; regulation of protein catabolic process; response to isoquinoline alkaloid; response to morphine; glucose import; monosaccharide transmembrane transport; amyloid-beta formation; tumor necrosis factor biosynthetic process; regulation of tumor necrosis factor biosynthetic process; hormone secretion; collagen metabolic process; regulation of purine nucleotide metabolic process; cell cycle arrest; positive regulation of chemokine biosynthetic process; epithelial cell differentiation involved in prostate gland development; cell aging; glial cell development; carbohydrate transmembrane transport; regulation of hemopoiesis; regulation of nucleotide metabolic process; memory; maternal placenta development; positive regulation of interleukin-12 production; regulation of amyloid precursor protein catabolic process; divalent metal ion transport; positive regulation of tyrosine phosphorylation of STAT protein; regulation of T cell activation; positive regulation of cell activation; glucose homeostasis; stress-activated protein kinase signaling cascade; maintenance of location; regulation of endothelial cell proliferation; response to heat; positive regulation of protein kinase B signaling; carbohydrate homeostasis; negative regulation of synaptic transmission; cellular response to estradiol stimulus; negative regulation of signal transduction in absence of ligand; negative regulation of extrinsic apoptotic signaling pathway in absence of ligand; hormone transport; regulation of lymphocyte activation; divalent inorganic cation transport; positive regulation of cytosolic calcium ion concentration; negative regulation of response to external stimulus; regulation of ATP metabolic process; positive regulation of binding; negative regulation of macrophage derived foam cell differentiation; regulation of tau-protein kinase activity; positive regulation of protein import into nucleus; T cell homeostasis; positive regulation of secretion by cell; response to growth hormone; negative regulation of vascular smooth muscle cell proliferation; regulation of B cell activation; regulation of mitochondrial membrane permeability; regulation of calcium ion transport; response to inactivity; regulation of MHC class II biosynthetic process; regulation of sensory perception of pain; positive regulation of protein import; steroid metabolic process; regulation of synaptic plasticity; regulation of protein localization to membrane; pancreas development; regulation of synaptic vesicle exocytosis; positive regulation of leukocyte migration; regulation of cell growth; positive regulation of lymphocyte activation; regulation of bone resorption; regulation of sensory perception; cellular response to osmotic stress; positive regulation of lymphocyte proliferation; endothelial cell proliferation; positive regulation of mononuclear cell proliferation; positive regulation of B cell proliferation; brown fat cell differentiation; regulation of neuroinflammatory response; regulation of hormone secretion; glial cell apoptotic process; MHC class II biosynthetic process; prepulse inhibition; Notch signaling pathway; striated muscle cell proliferation; extracellular matrix disassembly; signal transduction in response to DNA damage; regulation of chemokine production; positive regulation of secretion; mitochondrial membrane organization; DNA biosynthetic process; regulation of small molecule metabolic process; signal transduction by p53 class mediator; response to osmotic stress; regulation of tyrosine phosphorylation of STAT protein; regulation of transmembrane transporter activity; positive regulation of protein secretion; locomotory behavior; positive regulation of phospholipase C activity; cellular response to amyloid-beta; negative regulation of cysteine-type endopeptidase activity involved in apoptotic process; regulation of norepinephrine secretion; negative regulation of smooth muscle contraction; amyloid fibril formation; cellular response to heat; negative regulation of immune system process; lymphocyte proliferation; regulation of leukocyte differentiation; dopamine receptor signaling pathway; amyloid precursor protein catabolic process; positive regulation of T cell activation; mononuclear cell proliferation; positive regulation of leukocyte proliferation; leukocyte homeostasis; tyrosine phosphorylation of STAT protein; regulation of epidermal growth factor receptor signaling pathway; regulation of membrane permeability; regulation of long-term synaptic potentiation; regulation of phospholipase C activity; regulation of cytosolic calcium ion concentration; regulation of leukocyte mediated immunity; drug transport; response to UV-B; norepinephrine secretion; regulation of tissue remodeling; execution phase of apoptosis; regulation of epithelial cell apoptotic process; mesenchyme development; regulation of lipid storage; negative regulation of blood pressure; cellular response to interleukin-6; chemokine production; regulation of peptide hormone secretion; positive regulation of cation transmembrane transport; cellular response to acid chemical; somatic recombination of immunoglobulin genes involved in immune response; somatic diversification of immunoglobulins involved in immune response; acute-phase response; isotype switching; amyloid-beta metabolic process; regulation of extrinsic apoptotic signaling pathway in absence of ligand; bone remodeling; DNA damage checkpoint; regulation of neuron apoptotic process; regulation of immunoglobulin secretion; mammary gland alveolus development; mammary gland lobule development; negative regulation of secretion by cell; energy derivation by oxidation of organic compounds; stress-activated MAPK cascade; microglial cell activation; leukocyte activation involved in inflammatory response; prostate gland development; positive regulation of potassium ion transport; regulation of bone remodeling; myeloid cell homeostasis; cellular response to antibiotic; extracellular matrix organization; vesicle-mediated transport in synapse; negative regulation of cysteine-type endopeptidase activity; extracellular structure organization; positive regulation of peptide secretion; carbohydrate transport; response to calcium ion; cell death in response to oxidative stress; regulation of amine transport; regulation of ERBB signaling pathway; in utero embryonic development; cellular response to insulin stimulus; cellular response to tumor necrosis factor; alpha-beta T cell lineage commitment; striated muscle cell differentiation; response to interleukin-6; macrophage activation; regulation of protein localization to plasma membrane; positive regulation of immune effector process; embryo implantation; arachidonic acid metabolic process; negative regulation of G protein-coupled receptor signaling pathway; response to bronchodilator; osteoclast differentiation; mitotic DNA damage checkpoint; leukocyte proliferation; response to muramyl dipeptide; CD4-positive or CD8-positive, alpha-beta T cell lineage commitment; regulation of neurotransmitter uptake; dopamine transport; somatic recombination of immunoglobulin gene segments; positive regulation of neurogenesis; positive regulation of ion transmembrane transport; regulation of T cell proliferation; regulation of cellular amide metabolic process; DNA integrity checkpoint; amine transport; phasic smooth muscle contraction; response to corticosterone; response to hyperoxia; positive regulation of long-term synaptic potentiation; catecholamine metabolic process; catechol-containing compound metabolic process; alpha-beta T cell differentiation; cytokine production involved in immune response; ovarian follicle development; regulation of protein import into nucleus; B cell activation; negative regulation of protein binding; negative regulation of ion transmembrane transport; hormone metabolic process; regulation of receptor biosynthetic process; regulation of nuclease activity; vitamin D metabolic process; response to leptin; immunoglobulin secretion; regulation of neurotransmitter secretion; immunoglobulin production involved in immunoglobulin mediated immune response; DNA damage response, signal transduction by p53 class mediator resulting in cell cycle arrest; negative regulation of protein kinase activity; regulation of Notch signaling pathway; regulation of potassium ion transport; cardiac muscle tissue growth; positive regulation of neuron apoptotic process; signal transduction involved in mitotic G1 DNA damage checkpoint; intracellular signal transduction involved in G1 DNA damage checkpoint; regulation of stress-activated MAPK cascade; T cell activation involved in immune response; mitotic DNA integrity checkpoint; negative regulation of secretion; positive regulation of nucleotide biosynthetic process; positive regulation of glycolytic process; regulation of membrane protein ectodomain proteolysis; positive regulation of purine nucleotide biosynthetic process; negative regulation of defense response; regulation of stress-activated protein kinase signaling cascade; intracellular estrogen receptor signaling pathway; lipopolysaccharide-mediated signaling pathway; positive regulation of chemokine production; cellular response to glucocorticoid stimulus; regulation of protein import; negative regulation of inflammatory response; regulation of cell cycle arrest; epithelial tube morphogenesis; positive regulation of phospholipase activity; membrane protein proteolysis; positive regulation of DNA binding; signal transduction involved in mitotic cell cycle checkpoint; positive regulation of blood vessel diameter; signal transduction involved in mitotic DNA damage checkpoint; signal transduction involved in mitotic DNA integrity checkpoint; regulation of lipid metabolic process; long-chain fatty acid metabolic process; positive regulation of protein-containing complex assembly; CD4-positive, alpha-beta T cell differentiation involved in immune response; alpha-beta T cell activation involved in immune response; alpha-beta T cell differentiation involved in immune response; cellular response to corticosteroid stimulus; molting cycle; hair cycle; heart growth; negative regulation of mitotic cell cycle phase transition; positive regulation of osteoclast differentiation; branching involved in mammary gland duct morphogenesis; morphogenesis of an epithelial fold; regulation of insulin secretion; regulation of organ growth; lymphocyte homeostasis; somatic diversification of immune receptors via germline recombination within a single locus; somatic cell DNA recombination; regulation of catecholamine secretion; protein insertion into membrane; positive regulation of organ growth; regulation of protein localization to cell periphery; regulation of mitochondrion organization; positive regulation of apoptotic signaling pathway; T cell lineage commitment; T-helper 17 cell differentiation; regulation of cation channel activity; somatic diversification of immunoglobulins; positive regulation of fat cell differentiation; regulation of osteoclast differentiation; catecholamine secretion; regulation of glutamate receptor signaling pathway; regulation of protein-containing complex assembly; regulation of NIK/NF-kappaB signaling; negative regulation of kinase activity; regulation of monooxygenase activity; endothelial cell apoptotic process; NIK/NF-kappaB signaling; startle response; positive regulation of G2/M transition of mitotic cell cycle; negative regulation of muscle contraction; protein activation cascade; blood coagulation, fibrin clot formation; negative regulation of cellular response to drug; T cell proliferation; epidermal growth factor receptor signaling pathway; secondary metabolic process; regulation of ion transmembrane transporter activity; negative regulation of cell development; positive regulation of autophagy; synaptic vesicle exocytosis; cardiac muscle cell proliferation; prostaglandin biosynthetic process; mammary gland epithelial cell proliferation; response to increased oxygen levels; prostanoid biosynthetic process; response to angiotensin; erythrocyte homeostasis; positive regulation of protein localization to membrane; T cell differentiation involved in immune response; lipid storage; cell cycle G2/M phase transition; negative regulation of cell cycle phase transition; regulation of gliogenesis; lymphocyte differentiation; regulation of glycogen biosynthetic process; synaptic transmission, cholinergic; regulation of glucan biosynthetic process; T-helper 17 type immune response; regulation of phospholipase activity; regulation of synaptic transmission, glutamatergic; synaptic vesicle cycle; cofactor metabolic process; cell fate commitment; peptidyl-threonine phosphorylation; negative regulation of proteolysis; cellular response to ionizing radiation; regulation of macrophage derived foam cell differentiation; long-chain fatty acid biosynthetic process; cellular response to gamma radiation; endothelial cell migration; positive regulation of endothelial cell migration; negative regulation of transmembrane transport; negative regulation of cell cycle process; regulation of mitochondrial membrane potential; positive regulation of lipase activity; positive regulation of peptidase activity; cellular hormone metabolic process; activation of phospholipase C activity; response to X-ray; modulation by virus of host process; positive regulation of multicellular organism growth; positive regulation of epidermal growth factor receptor signaling pathway; B cell activation involved in immune response; multicellular organismal response to stress; regulation of glial cell differentiation; signal transduction involved in DNA integrity checkpoint; signal transduction involved in DNA damage checkpoint; regulation of carbohydrate metabolic process; cellular response to nutrient; CD4-positive, alpha-beta T cell differentiation; signal transduction involved in cell cycle checkpoint; regulation of neurotransmitter receptor activity; regulation of fat cell differentiation; response to amphetamine; regulation of synaptic transmission, GABAergic; response to arsenic-containing substance; response to mineralocorticoid; positive regulation of synaptic transmission, glutamatergic; somatic diversification of immune receptors; regulation of oxidative stress-induced cell death; regulation of ossification; protein deubiquitination; peptidyl-threonine modification; entry into host; regulation of endocytosis; positive regulation of chemotaxis; eating behavior; regulation of isotype switching; mammary gland duct morphogenesis; positive regulation of ERBB signaling pathway; production of molecular mediator of immune response; regulation of glycolytic process; adult locomotory behavior; positive regulation of NIK/NF-kappaB signaling; female gamete generation; insulin secretion; associative learning; regulation of glucose transmembrane transport; intrinsic apoptotic signaling pathway by p53 class mediator; regulation of interleukin-17 production; anoikis; positive T cell selection; G protein-coupled serotonin receptor signaling pathway; alpha-beta T cell activation; forebrain development; positive regulation of carbohydrate metabolic process; fatty acid metabolic process; drug catabolic process; type 2 immune response; regulation of glycogen metabolic process; positive regulation of mitochondrial outer membrane permeabilization involved in apoptotic signaling pathway; negative regulation of response to drug; muscle cell differentiation; negative regulation of neurogenesis; insulin receptor signaling pathway; negative regulation of cytokine production; positive regulation of protein catabolic process; regulation of G protein-coupled receptor signaling pathway; positive regulation of B cell activation; positive regulation of cell cycle arrest; macrophage derived foam cell differentiation; monocyte differentiation; positive regulation of DNA replication; positive regulation of telomerase activity; foam cell differentiation; amyloid-beta clearance; mononuclear cell differentiation; regulation of NMDA receptor activity; protein modification by small protein removal; striated muscle tissue development; adult behavior; negative regulation of protein serine/threonine kinase activity; behavioral fear response; regulation of polysaccharide biosynthetic process; response to fluid shear stress; positive regulation of blood pressure; regulation of cytokine production involved in immune response; positive regulation of lipid metabolic process; chromatin remodeling; mesenchymal cell differentiation; regulation of sodium ion transport; mesenchymal cell development; phosphatidylinositol 3-kinase signaling; behavioral defense response; activation of adenylate cyclase activity; histone phosphorylation; response to food; interleukin-17 production; positive regulation of glucose import; positive regulation of cardiac muscle cell proliferation; hair follicle development; extrinsic apoptotic signaling pathway via death domain receptors; cellular carbohydrate biosynthetic process; response to fatty acid; negative regulation of immune response; embryonic placenta development; positive regulation of leukocyte chemotaxis; energy reserve metabolic process; regulation of carbohydrate catabolic process; positive regulation of striated muscle tissue development; positive regulation of muscle organ development; serotonin receptor signaling pathway; regulation of intracellular estrogen receptor signaling pathway; dopamine metabolic process; negative regulation of extrinsic apoptotic signaling pathway via death domain receptors; molting cycle process; hair cycle process; regulation of protein tyrosine kinase activity; skin epidermis development; neuron projection organization; regulation of cellular response to oxidative stress; positive regulation of muscle tissue development; receptor-mediated endocytosis; synapse organization; muscle tissue development; positive regulation of inflammatory response; regulation of endothelial cell migration; mating; negative regulation of cytokine biosynthetic process; fear response; modulation by symbiont of host process; regulation of purine nucleotide biosynthetic process; negative regulation of nervous system development; muscle organ development; monovalent inorganic cation homeostasis; adenylate cyclase-activating dopamine receptor signaling pathway; peristalsis; response to cobalt ion; negative regulation of tumor necrosis factor biosynthetic process; low-density lipoprotein particle receptor biosynthetic process; behavioral response to ethanol; positive regulation of isotype switching to IgG isotypes; prostatic bud formation; response to lipoteichoic acid; regulation of uterine smooth muscle contraction; cellular response to lipoteichoic acid; cellular response to iron ion; positive regulation of brown fat cell differentiation; neuron projection maintenance; regulation of nucleotide biosynthetic process; retinol metabolic process; modulation of excitatory postsynaptic potential; regulation of extracellular matrix organization; membrane depolarization; negative regulation of cation transmembrane transport; regulation of epithelial cell differentiation; regulation of lipid localization; lipid localization; CD4-positive, alpha-beta T cell activation; movement in host environment; calcium ion transport into cytosol; response to virus; positive regulation of immunoglobulin production; membrane protein ectodomain proteolysis; response to copper ion; body fluid secretion; synaptic transmission, glutamatergic; regulation of tumor necrosis factor production; regulation of reproductive process; prostanoid metabolic process; prostaglandin metabolic process; fat-soluble vitamin metabolic process; intrinsic apoptotic signaling pathway in response to oxidative stress; regulation of polysaccharide metabolic process; negative regulation of Notch signaling pathway; regulation of cellular response to drug; interleukin-6 production; nervous system process involved in regulation of systemic arterial blood pressure; cerebral cortex GABAergic interneuron differentiation; response to peptidoglycan; Leydig cell differentiation; histone-serine phosphorylation; positive regulation of immunoglobulin secretion; prostate gland growth; uterine smooth muscle contraction; T cell differentiation; positive regulation of production of molecular mediator of immune response; positive regulation of T cell proliferation; monocarboxylic acid transport; tumor necrosis factor production; regulation of tumor necrosis factor superfamily cytokine production; vascular endothelial growth factor receptor signaling pathway; positive regulation of glucose transmembrane transport; macrophage differentiation; regulation of collagen biosynthetic process; positive regulation of tissue remodeling; regulation of mitochondrial outer membrane permeabilization involved in apoptotic signaling pathway; fatty acid biosynthetic process; fatty acid transport; regulation of response to oxidative stress; negative regulation of cell migration; endocrine pancreas development; intrinsic apoptotic signaling pathway in response to DNA damage by p53 class mediator; thymus development; protein insertion into mitochondrial membrane; synaptic transmission, GABAergic; regulation of cell killing; pigmentation; regulation of lipase activity; G2/M transition of mitotic cell cycle; cardiocyte differentiation; modulation of process of other organism involved in symbiotic interaction; cell killing; tumor necrosis factor superfamily cytokine production; magnesium ion homeostasis; positive regulation of synaptic transmission, GABAergic; response to vitamin E; opioid receptor signaling pathway; vitamin D biosynthetic process; positive regulation of action potential; saliva secretion; regulation of isotype switching to IgG isotypes; branching involved in prostate gland morphogenesis; T-helper 17 cell lineage commitment; hepatocyte apoptotic process; glandular epithelial cell differentiation; inflammatory response to antigenic stimulus; regulation of acute inflammatory response; glycogen biosynthetic process; glucan biosynthetic process; response to amine; ovulation cycle process; oocyte development; mammary gland morphogenesis; labyrinthine layer development; regulation of cytokine production involved in inflammatory response; negative regulation of epithelial cell apoptotic process; telencephalon development; negative regulation of growth; glutamate receptor signaling pathway; positive regulation of cell growth; negative regulation of endopeptidase activity; neurotransmitter secretion; negative regulation of DNA-binding transcription factor activity; signal release from synapse; collagen catabolic process; neuron maturation; T cell selection; developmental pigmentation; genitalia development; response to epidermal growth factor; establishment of protein localization to mitochondrial membrane; regulation of macroautophagy; visual learning; positive regulation of cardiac muscle tissue growth; regulation of release of cytochrome c from mitochondria; adenohypophysis development; T-helper 2 cell cytokine production; isotype switching to IgG isotypes; regulation of interleukin-1 beta biosynthetic process; lateral sprouting from an epithelium; eyelid development in camera-type eye; nucleotide-binding oligomerization domain containing 2 signaling pathway; positive regulation of ATP biosynthetic process; Ras protein signal transduction; stem cell differentiation; negative regulation of cell motility; cytokine production involved in inflammatory response; regulation of collagen metabolic process; digestive tract morphogenesis; mitochondrial transport; cytosolic calcium ion transport; regulation of response to drug; DNA damage response, signal transduction by p53 class mediator; neuromuscular process; regulation of oxidoreductase activity; negative regulation of peptidase activity; regulation of response to wounding; regulation of B cell mediated immunity; regulation of immunoglobulin mediated immune response; oocyte differentiation; regulation of nitric-oxide synthase activity; regulation of signal transduction by p53 class mediator; regulation of DNA replication; response to retinoic acid; genitalia morphogenesis; positive regulation of regulatory T cell differentiation; interleukin-1 beta biosynthetic process; positive regulation of killing of cells of other organism; growth hormone receptor signaling pathway via JAK-STAT; cellular response to estrogen stimulus; GABAergic neuron differentiation; positive regulation of metallopeptidase activity; phosphatidylinositol-mediated signaling; limbic system development; positive regulation of heart growth; adaptive immune response based on somatic recombination of immune receptors built from immunoglobulin superfamily domains; diterpenoid metabolic process; unsaturated fatty acid metabolic process; cellular transition metal ion homeostasis; eye development; inositol lipid-mediated signaling; visual behavior; telencephalon cell migration; collagen biosynthetic process; response to dsRNA; positive regulation of mitotic nuclear division; regulation of JNK cascade; columnar/cuboidal epithelial cell differentiation; visual system development; male sex determination; fat-soluble vitamin biosynthetic process; regulation of interleukin-1 biosynthetic process; positive regulation of interleukin-6 biosynthetic process; regulation of developmental pigmentation; positive regulation of membrane protein ectodomain proteolysis; unsaturated fatty acid biosynthetic process; negative regulation of DNA binding; mitochondrial outer membrane permeabilization; glycolytic process; modulation of process of other organism; regulation of gene silencing by miRNA; DNA replication; regulation of nuclear division; regulation of leukocyte chemotaxis; icosanoid metabolic process; ATP generation from ADP; erythrocyte differentiation; alcohol metabolic process; icosanoid biosynthetic process; positive regulation of protein tyrosine kinase activity; sensory system development; response to starvation; positive regulation of mitochondrion organization; kidney development; forebrain cell migration; T cell costimulation; regulation of morphogenesis of a branching structure; positive regulation of protein localization to plasma membrane; negative regulation of oxidative stress-induced cell death; epithelial cell maturation; T-helper cell lineage commitment; regulation of cytokine secretion involved in immune response; serotonin transport; negative regulation of B cell proliferation; interleukin-1 biosynthetic process; hormone catabolic process; estrous cycle; hepatocyte growth factor receptor signaling pathway; ventricular cardiac muscle cell differentiation; morphogenesis of an epithelial bud; negative regulation of gene silencing by miRNA; cellular response to prostaglandin E stimulus; regulation of brown fat cell differentiation; positive regulation of glutamate receptor signaling pathway; regulation of posttranscriptional gene silencing; regulation of gene silencing by RNA; immunoglobulin production; lymphocyte costimulation; hydrogen peroxide metabolic process; regulation of cardiac muscle cell proliferation; cellular response to fatty acid; negative regulation of cellular response to oxidative stress; lipid transport; negative regulation of locomotion; T-helper cell differentiation; regulation of extrinsic apoptotic signaling pathway via death domain receptors; regulation of protein stability; renal system process; terpenoid metabolic process; response to endoplasmic reticulum stress; regulation of angiogenesis; regulation of extracellular matrix disassembly; response to insecticide; female genitalia development; heat generation; positive regulation of interleukin-17 production; negative regulation of collagen biosynthetic process; cellular response to hepatocyte growth factor stimulus; cytoplasmic sequestering of transcription factor; CD4-positive, alpha-beta T cell lineage commitment; positive regulation of glycogen biosynthetic process; hydrogen peroxide biosynthetic process; regulation of killing of cells of other organism; positive regulation of leukocyte adhesion to vascular endothelial cell; negative regulation of anoikis; negative regulation of cellular component movement; viral entry into host cell; negative regulation of response to oxidative stress; regulation of endothelial cell apoptotic process; negative regulation of cell activation; antibiotic metabolic process; ADP metabolic process; regulation of glucose import; positive regulation of mitochondrial membrane permeability involved in apoptotic process; regulation of phosphatidylinositol 3-kinase signaling; positive regulation of wound healing; positive regulation of intrinsic apoptotic signaling pathway; regulation of B cell apoptotic process; negative regulation of collagen metabolic process; striatum development; cellular response to leptin stimulus; positive regulation of bone resorption; positive regulation of bone remodeling; positive regulation of glycogen metabolic process; regulation of amyloid-beta clearance; renal system development; xenobiotic metabolic process; regulation of lipid transport; positive regulation of angiogenesis; killing of cells of other organism; positive regulation of cardiac muscle tissue development; intrinsic apoptotic signaling pathway in response to endoplasmic reticulum stress; mitochondrial outer membrane permeabilization involved in programmed cell death; positive regulation of translation; cellular iron ion homeostasis; excretion; positive regulation of protein localization to cell periphery; response to interleukin-1; chronic inflammatory response; negative regulation of systemic arterial blood pressure; grooming behavior; gas transport; response to magnesium ion; response to vitamin A; response to hepatocyte growth factor; negative regulation of posttranscriptional gene silencing; labyrinthine layer blood vessel development; negative regulation of gene silencing by RNA; negative regulation of release of cytochrome c from mitochondria; regulation of production of miRNAs involved in gene silencing by miRNA; regulation of ATP biosynthetic process; negative regulation of leukocyte cell-cell adhesion; positive regulation of mitochondrial membrane permeability; animal organ formation; positive regulation of sprouting angiogenesis; negative regulation of reactive oxygen species metabolic process; B cell differentiation; ATP metabolic process; cell chemotaxis; regulation of cell cycle G2/M phase transition; nucleoside diphosphate phosphorylation; negative regulation of protein catabolic process; cytokine secretion involved in immune response; response to manganese ion; platelet formation; positive regulation of smooth muscle cell apoptotic process; regulation of dopamine metabolic process; regulation of catecholamine metabolic process; positive regulation of tumor necrosis factor biosynthetic process; regulation of monocyte differentiation; behavioral response to cocaine; negative regulation of endoplasmic reticulum stress-induced intrinsic apoptotic signaling pathway; positive regulation of nuclear division; regulation of mitochondrial membrane permeability involved in apoptotic process; positive regulation of membrane permeability; JNK cascade; action potential; transition metal ion homeostasis; positive regulation of DNA biosynthetic process; vitamin metabolic process; purine nucleoside diphosphate metabolic process; purine ribonucleoside diphosphate metabolic process; nucleotide phosphorylation; regulation of chemotaxis; positive regulation of macroautophagy; cell adhesion mediated by integrin; cellular response to vascular endothelial growth factor stimulus; multicellular organismal water homeostasis; cellular response to amino acid stimulus; desensitization of G protein-coupled receptor signaling pathway; acute inflammatory response to antigenic stimulus; antibiotic biosynthetic process; negative adaptation of signaling pathway; ovulation; negative regulation of chemokine production; peroxisome proliferator activated receptor signaling pathway; CD4-positive, alpha-beta T cell cytokine production; platelet morphogenesis; positive regulation of protein export from nucleus; regulation of production of small RNA involved in gene silencing by RNA; cellular response to fluid shear stress; positive regulation of extracellular matrix organization; positive regulation of leukocyte mediated immunity; ribonucleoside diphosphate metabolic process; regulation of cellular carbohydrate metabolic process; establishment of protein localization to mitochondrion; long-chain fatty acid transport; lymphocyte apoptotic process; negative regulation of tumor necrosis factor production; isoprenoid metabolic process; positive regulation of cytokine secretion; regulation of gene silencing; melanin metabolic process; mating behavior; adaptation of signaling pathway; positive regulation of cyclase activity; endoplasmic reticulum calcium ion homeostasis; negative regulation of Rho protein signal transduction; regulation of urine volume; cellular response to prostaglandin stimulus; regulation of vasculature development; cardiac muscle contraction; positive regulation of cellular protein catabolic process; calcium ion transmembrane transport; leukocyte chemotaxis; cellular polysaccharide biosynthetic process; pigment metabolic process; regulation of translation; negative regulation of protein secretion; protein localization to mitochondrion; production of molecular mediator involved in inflammatory response; negative regulation of tumor necrosis factor superfamily cytokine production; cold-induced thermogenesis; regulation of cold-induced thermogenesis; low-density lipoprotein receptor particle metabolic process; response to prostaglandin E; negative regulation of lipid catabolic process; establishment of skin barrier; negative regulation of cell cycle arrest; negative regulation of response to reactive oxygen species; negative regulation of intrinsic apoptotic signaling pathway by p53 class mediator; negative regulation of hydrogen peroxide-induced cell death; positive regulation of gene silencing by miRNA; negative regulation of mononuclear cell proliferation; negative regulation of lymphocyte proliferation; positive regulation of vasculature development; glycogen metabolic process; positive regulation of gliogenesis; water homeostasis; positive regulation of response to wounding; regulation of adaptive immune response based on somatic recombination of immune receptors built from immunoglobulin superfamily domains; cellular glucan metabolic process; regulation of intracellular steroid hormone receptor signaling pathway; glucan metabolic process; B cell apoptotic process; regulation of receptor recycling; sex determination; vitamin biosynthetic process; response to lead ion; subpallium development; cerebral cortex neuron differentiation; exogenous drug catabolic process; fibroblast apoptotic process; decidualization; cytoplasmic sequestering of protein; positive regulation of posttranscriptional gene silencing; growth hormone receptor signaling pathway; regulation of anoikis; cardiac muscle tissue development; negative regulation of neuron apoptotic process; negative regulation of peptide secretion; regulation of wound healing; protein autophosphorylation; regulation of lymphocyte mediated immunity; cellular response to starvation; response to axon injury; monocarboxylic acid biosynthetic process; negative regulation of cytokine production involved in immune response; hair follicle morphogenesis; negative regulation of cAMP-mediated signaling; positive regulation of isotype switching; regulation of response to interferon-gamma; regulation of interferon-gamma-mediated signaling pathway; cellular response to growth hormone stimulus; negative regulation of regulated secretory pathway; cellular response to angiotensin; positive regulation of transcription regulatory region DNA binding; pyruvate metabolic process; lipid modification; polysaccharide biosynthetic process; central nervous system neuron development; negative regulation of ion transmembrane transporter activity; regulation of cardiac muscle tissue growth; negative regulation of leukocyte proliferation; positive regulation of reproductive process; regulation of striated muscle tissue development; regulation of interleukin-6 production; regulation of calcium ion transmembrane transport; potassium ion transport; cytokine secretion; positive regulation of blood vessel endothelial cell migration; cellular response to mechanical stimulus; regulation of cellular response to heat; Fc receptor signaling pathway; oocyte maturation; apoptotic DNA fragmentation; positive regulation of heart rate; positive regulation of blood coagulation; melanocyte differentiation; regulation of water loss via skin; regulation of long-term neuronal synaptic plasticity; response to thyroid hormone; positive regulation of hemostasis; regulation of receptor binding; regulation of protein insertion into mitochondrial membrane involved in apoptotic signaling pathway; positive regulation of protein insertion into mitochondrial membrane involved in apoptotic signaling pathway; regulation of oxidative stress-induced neuron death; regulation of metallopeptidase activity; nucleoside diphosphate metabolic process; adaptive thermogenesis; regulation of muscle tissue development; negative regulation of hemopoiesis; hexose metabolic process; neural crest cell development; hippocampus development; positive regulation of proteasomal ubiquitin-dependent protein catabolic process; iron ion homeostasis; negative regulation of response to DNA damage stimulus; regulation of blood vessel endothelial cell migration; regulation of muscle organ development; regulation of epidermal growth factor-activated receptor activity; neuron death in response to oxidative stress; positive regulation of coagulation; regulation of ventricular cardiac muscle cell membrane repolarization; cellular response to copper ion; regulation of oxidative stress-induced intrinsic apoptotic signaling pathway; positive regulation of DNA-templated transcription, initiation; regulation of cardiac muscle contraction; positive regulation of T cell differentiation; cellular response to calcium ion; myeloid dendritic cell activation; protein kinase C-activating G protein-coupled receptor signaling pathway; androgen metabolic process; response to gonadotropin; regulation of interleukin-6 biosynthetic process; positive regulation of G protein-coupled receptor signaling pathway; positive regulation of chromosome segregation; protein localization to chromatin; regulation of leukocyte adhesion to vascular endothelial cell; positive regulation of excitatory postsynaptic potential; regulation of adaptive immune response; skeletal muscle tissue development; lymphocyte mediated immunity; positive regulation of lipid biosynthetic process; regulation of heart growth; negative regulation of hydrolase activity; secondary alcohol metabolic process; primary alcohol metabolic process; stem cell development; regulation of animal organ morphogenesis; telomere maintenance; regulation of transcription involved in G1/S transition of mitotic cell cycle; nitric oxide mediated signal transduction; adult walking behavior; positive regulation of vascular endothelial growth factor production; positive regulation of peptidyl-threonine phosphorylation; interleukin-6 biosynthetic process; negative regulation of heart contraction; epidermis morphogenesis; interleukin-6-mediated signaling pathway; hormone biosynthetic process; regulation of mitotic nuclear division; oogenesis; B cell homeostasis; protein insertion into mitochondrial membrane involved in apoptotic signaling pathway; reproductive behavior; regulation of fatty acid oxidation; negative regulation of fibroblast proliferation; negative regulation of B cell activation; regulation of hydrogen peroxide-induced cell death; defense response to Gram-negative bacterium; fatty acid derivative metabolic process; protein localization to plasma membrane; negative regulation of transporter activity; regulation of cell division; germ cell development; pallium development; Fc-epsilon receptor signaling pathway; skeletal muscle organ development; metanephros development; neural crest cell differentiation; regulation of cellular pH; odontogenesis of dentin-containing tooth; regulation of carbohydrate biosynthetic process; maintenance of protein location; positive regulation of ossification; positive regulation of acute inflammatory response; sleep; positive regulation of monooxygenase activity; response to prostaglandin; positive regulation of erythrocyte differentiation; regulation of astrocyte differentiation; walking behavior; ventricular cardiac muscle cell membrane repolarization; negative regulation of intrinsic apoptotic signaling pathway in response to DNA damage; regulation of intrinsic apoptotic signaling pathway by p53 class mediator; mitotic nuclear division; phagocytosis; smooth muscle cell migration; negative regulation of myeloid cell differentiation; interferon-gamma-mediated signaling pathway; organic hydroxy compound biosynthetic process; protein tetramerization; estrogen metabolic process; attachment of spindle microtubules to kinetochore; ERBB2 signaling pathway; vasodilation; retinoic acid metabolic process; regulation of regulatory T cell differentiation; response to lipoprotein particle; placenta blood vessel development; cellular response to vitamin; regulation of cardiac muscle cell membrane repolarization; regulation of endoplasmic reticulum stress-induced intrinsic apoptotic signaling pathway; positive regulation of protein binding; I-kappaB kinase/NF-kappaB signaling; negative regulation of leukocyte activation; telomere organization; positive regulation of lymphocyte differentiation; protein ADP-ribosylation; negative regulation of macroautophagy; myeloid cell apoptotic process; negative regulation of cyclin-dependent protein serine/threonine kinase activity; negative regulation of signal transduction by p53 class mediator; negative regulation of endothelial cell apoptotic process; positive regulation of ubiquitin-dependent protein catabolic process; regulation of cytokine-mediated signaling pathway; striated muscle contraction; synapse assembly; positive regulation of endopeptidase activity; regulation of muscle cell apoptotic process; regulation of fatty acid metabolic process; positive regulation of interleukin-6 production; DNA catabolic process, endonucleolytic; epithelial cell morphogenesis; regulation of vascular endothelial growth factor production; positive regulation of sodium ion transport; neuronal action potential; cell death in response to hydrogen peroxide; regulatory T cell differentiation; negative regulation of exocytosis; negative regulation of gene silencing; neuron cellular homeostasis; cellular response to lipoprotein particle stimulus; negative regulation of cyclin-dependent protein kinase activity; cellular response to interleukin-1; regulation of striated muscle contraction; positive regulation of cold-induced thermogenesis; positive regulation of response to DNA damage stimulus; negative regulation of cell-cell adhesion; blood vessel endothelial cell migration; cellular response to interferon-gamma; positive regulation of defense response; nucleotide biosynthetic process; cell migration involved in sprouting angiogenesis; regulation of pH; positive regulation of endocytosis; receptor recycling; negative regulation of production of molecular mediator of immune response; apoptotic nuclear changes; regulation of glial cell proliferation; nucleotide-binding oligomerization domain containing signaling pathway; fatty acid oxidation; fatty acid derivative biosynthetic process; sprouting angiogenesis; cellular carbohydrate metabolic process; nucleoside phosphate biosynthetic process; positive regulation of adaptive immune response based on somatic recombination of immune receptors built from immunoglobulin superfamily domains; muscle cell apoptotic process; digestive system process; regulation of cardiac muscle tissue development; interleukin-1-mediated signaling pathway; vascular endothelial growth factor production; regulation of vascular endothelial growth factor receptor signaling pathway; smooth muscle cell apoptotic process; regulation of smooth muscle cell apoptotic process; nucleotide-binding domain, leucine rich repeat containing receptor signaling pathway; negative regulation of insulin receptor signaling pathway; pigment cell differentiation; negative regulation of calcium ion transmembrane transporter activity; gastrulation; regulation of DNA recombination; lipid oxidation; regulation of receptor-mediated endocytosis; negative regulation of cell growth; regulation of calcium ion transport into cytosol; positive regulation of B cell mediated immunity; positive regulation of immunoglobulin mediated immune response; cellular component disassembly involved in execution phase of apoptosis; Schwann cell differentiation; secretion by tissue; response to vitamin D; peptidyl-tyrosine autophosphorylation; positive regulation of protein-containing complex disassembly; positive regulation of fatty acid metabolic process; regulation of activated T cell proliferation; membrane depolarization during action potential; positive regulation of cell migration involved in sprouting angiogenesis; negative regulation of reactive oxygen species biosynthetic process; regulation of DNA-templated transcription, initiation; monosaccharide metabolic process; regulation of interleukin-1 production; cellular polysaccharide metabolic process; negative regulation of leukocyte differentiation; regulation of response to cytokine stimulus; retinoid metabolic process; muscle cell migration; positive regulation of proteasomal protein catabolic process; porphyrin-containing compound metabolic process; positive regulation of myotube differentiation; positive regulation of glucose metabolic process; negative regulation of insulin secretion; regulation of protein export from nucleus; insulin-like growth factor receptor signaling pathway; positive regulation of neurotransmitter transport; ventricular cardiac muscle cell action potential; regulation of heart rate by cardiac conduction; negative regulation of cellular response to insulin stimulus; negative regulation of blood circulation; positive regulation of epithelial cell apoptotic process; positive regulation of lymphocyte mediated immunity; positive regulation of adaptive immune response; negative regulation of protein transport; T cell mediated immunity; organ or tissue specific immune response; activation of JUN kinase activity; positive regulation of muscle cell apoptotic process; regulation of vascular permeability; positive regulation of DNA recombination; regulation of membrane repolarization; regulation of intrinsic apoptotic signaling pathway in response to DNA damage; cellular respiration; macroautophagy; leukocyte mediated cytotoxicity; nuclear division; cellular monovalent inorganic cation homeostasis; regulation of G2/M transition of mitotic cell cycle; regulation of systemic arterial blood pressure by hormone; T cell cytokine production; positive regulation of heart contraction; positive regulation of release of sequestered calcium ion into cytosol; negative regulation of establishment of protein localization; carbohydrate catabolic process; DNA catabolic process; activated T cell proliferation; regulation of execution phase of apoptosis; positive regulation of potassium ion transmembrane transport; response to interferon-gamma; receptor internalization; lymphocyte migration; negative regulation of cellular carbohydrate metabolic process; pituitary gland development; cardiac muscle cell membrane repolarization; regulation of response to reactive oxygen species; regulation of glucose metabolic process; negative regulation of T cell activation; regulation of innate immune response; response to hexose; skin development; negative regulation of developmental growth; glucose metabolic process; positive regulation of supramolecular fiber organization; regulation of cyclase activity; regulation of protein oligomerization; cellular response to amino acid starvation; positive regulation of glial cell differentiation; cellular response to epidermal growth factor stimulus; negative regulation of calcium ion transmembrane transport; polysaccharide metabolic process; regulation of circadian rhythm; interleukin-1 production; response to monosaccharide; phenol-containing compound biosynthetic process; leukocyte adhesion to vascular endothelial cell; negative regulation of response to endoplasmic reticulum stress; carbohydrate biosynthetic process; icosanoid secretion; response to progesterone; endodermal cell differentiation; autonomic nervous system development; negative regulation of peptide hormone secretion; regulation of cytokine secretion; purine nucleotide metabolic process; ER-nucleus signaling pathway; negative regulation of lipid transport; production of miRNAs involved in gene silencing by miRNA; response to exogenous dsRNA; response to amino acid starvation; camera-type eye development; protein localization to cell periphery; negative regulation of immune effector process; import across plasma membrane; positive regulation of proteolysis involved in cellular protein catabolic process; chromosome segregation; formation of primary germ layer; regulation of peptidyl-threonine phosphorylation; positive regulation of smooth muscle cell migration; interleukin-12-mediated signaling pathway; regulation of CD4-positive, alpha-beta T cell differentiation; regulation of erythrocyte differentiation; regulation of proteasomal ubiquitin-dependent protein catabolic process; membrane raft; membrane microdomain; membrane region; integral component of presynaptic membrane; intrinsic component of presynaptic membrane; integral component of postsynaptic membrane; intrinsic component of postsynaptic membrane; integral component of synaptic membrane; presynaptic membrane; intrinsic component of synaptic membrane; plasma membrane raft; caveola; presynapse; postsynaptic membrane; cyclin-dependent protein kinase holoenzyme complex; glutamatergic synapse; synaptic membrane; protein kinase complex; serine/threonine protein kinase complex; neuronal cell body; spindle; RNA polymerase II transcription regulator complex; axon terminus; dopaminergic synapse; neuron projection terminus; distal axon; nuclear chromatin; myelin sheath; integral component of postsynaptic density membrane; intrinsic component of postsynaptic density membrane; transcription regulator complex; asymmetric synapse; transferase complex, transferring phosphorus-containing groups; neuron to neuron synapse; postsynaptic density membrane; integral component of postsynaptic specialization membrane; perikaryon; intrinsic component of postsynaptic specialization membrane; focal adhesion; cell-substrate junction; dendrite membrane; postsynaptic density; cell projection membrane; dendritic spine; cholinergic synapse; neuron spine; leading edge membrane; postsynaptic specialization; postsynaptic specialization membrane; Golgi lumen; nuclear envelope; T-tubule; clathrin-coated vesicle; neuron projection membrane; coated vesicle; external side of plasma membrane; protein-DNA complex; ficolin-1-rich granule; ficolin-1-rich granule lumen; endoplasmic reticulum lumen; basolateral plasma membrane; sarcolemma; GABA-ergic synapse; lamellipodium membrane; glial cell projection; neuronal cell body membrane; microvillus; cell body membrane; dendrite cytoplasm; mitochondrial outer membrane; basal plasma membrane; cell leading edge; collagen-containing extracellular matrix; synaptic vesicle; synaptic vesicle membrane; exocytic vesicle membrane; nuclear membrane; melanosome; pigment granule; cell-cell junction; endocytic vesicle; G protein-coupled amine receptor activity; adrenergic receptor activity; nuclear receptor activity; ligand-activated transcription factor activity; catecholamine binding; steroid hormone receptor activity; RNA polymerase II-specific DNA-binding transcription factor binding; DNA-binding transcription factor binding; ubiquitin-like protein ligase binding; ubiquitin protein ligase binding; phosphatase binding; protein phosphatase binding; hormone binding; activating transcription factor binding; peptide binding; steroid binding; postsynaptic neurotransmitter receptor activity; amide binding; neurotransmitter receptor activity; cyclin-dependent protein serine/threonine kinase regulator activity; acetylcholine receptor activity; hydrolase activity, acting on acid phosphorus-nitrogen bonds; serine hydrolase activity; NF-kappaB binding; endopeptidase activity; sodium:chloride symporter activity; BH domain binding; death domain binding; DNA-binding transcription activator activity, RNA polymerase II-specific; cytokine receptor binding; DNA-binding transcription activator activity; oxidoreductase activity, acting on paired donors, with incorporation or reduction of molecular oxygen; serine-type endopeptidase activity; protein phosphatase 2A binding; nuclear receptor binding; anion:sodium symporter activity; dopamine binding; serine-type peptidase activity; protease binding; cysteine-type endopeptidase activity involved in apoptotic process; G protein-coupled neurotransmitter receptor activity; cation:chloride symporter activity; growth factor receptor binding; heme binding; acetylcholine binding; kinase regulator activity; tetrapyrrole binding; steroid hormone receptor binding; anion:cation symporter activity; phosphoric diester hydrolase activity; nuclear hormone receptor binding; neurotransmitter binding; scaffold protein binding; phosphatidylinositol phospholipase C activity; neurotransmitter:sodium symporter activity; protein kinase regulator activity; phospholipase C activity; hormone receptor binding; repressing transcription factor binding; virus receptor activity; exogenous protein binding; disordered domain specific binding; G protein-coupled serotonin receptor activity; serotonin receptor activity; amyloid-beta binding; signaling receptor activator activity; cytokine activity; dopamine neurotransmitter receptor activity; Hsp90 protein binding; estrogen receptor binding; protein serine/threonine/tyrosine kinase activity; transcription coactivator activity; organic hydroxy compound transmembrane transporter activity; growth factor activity; cyclin-dependent protein serine/threonine kinase inhibitor activity; insulin-like growth factor I binding; monooxygenase activity; phospholipase activity; metalloendopeptidase activity; MAP kinase activity; nitric-oxide synthase binding; neurotransmitter transmembrane transporter activity; RNA polymerase II activating transcription factor binding; MAP kinase kinase activity; receptor ligand activity; transmembrane receptor protein tyrosine kinase activity; adrenergic receptor binding; DNA polymerase binding; protein kinase inhibitor activity; lipase activity; RNA polymerase II general transcription initiation factor binding; p53 binding; kinase inhibitor activity; growth factor binding; transcription coactivator binding; fibroblast growth factor binding; R-SMAD binding; solute:sodium symporter activity; G-protein alpha-subunit binding; neuropeptide binding; receptor serine/threonine kinase binding; sodium ion transmembrane transporter activity; transmembrane receptor protein kinase activity; metal ion transmembrane transporter activity; ATPase binding; beta-catenin binding; insulin-like growth factor binding; kinase activator activity; oxidoreductase activity, acting on paired donors, with incorporation or reduction of molecular oxygen, reduced flavin or flavoprotein as one donor, and incorporation of one atom of oxygen protein serine/threonine kinase inhibitor activity | 6.01281E-19; 3.75119E-17; 7.59211E-17; 2.56189E-16; 2.58393E-16; 2.58393E-16; 2.58393E-16; 2.58393E-16; 5.14535E-16; 6.77316E-16; 7.80211E-16; 1.72569E-15; 2.22318E-15; 8.2268E-15; 1.60607E-14; 2.09374E-14; 2.6083E-14; 6.73841E-14; 3.55758E-13; 3.55758E-13; 4.69546E-13; 4.91242E-13; 1.00564E-12; 1.13856E-12; 1.42603E-12; 1.44207E-12; 2.45965E-12; 2.56001E-12; 5.2781E-12; 1.45461E-11; 1.52866E-11; 1.69074E-11; 1.69074E-11; 1.73338E-11; 3.69901E-11; 3.69901E-11; 3.98449E-11; 4.78816E-11; 5.93515E-11; 6.89677E-11; 1.05115E-10; 1.29424E-10; 1.31302E-10; 2.27759E-10; 2.28246E-10; 2.43485E-10; 2.47084E-10; 2.81298E-10; 3.17047E-10; 3.48777E-10; 3.68003E-10; 3.69096E-10; 7.23085E-10; 7.75422E-10; 9.5907E-10; 1.06977E-09; 1.5259E-09; 1.52661E-09; 2.13754E-09; 2.25512E-09; 2.87004E-09; 2.88402E-09; 3.24309E-09; 3.86459E-09; 5.08169E-09; 5.12094E-09; 5.12094E-09; 5.37131E-09; 5.37131E-09; 5.41896E-09; 5.57784E-09; 5.65733E-09; 5.65733E-09; 6.3979E-09; 7.3476E-09; 8.16654E-09; 8.16654E-09; 9.0187E-09; 1.11807E-08; 1.2608E-08; 1.31117E-08; 1.35811E-08; 1.37984E-08; 1.50391E-08; 1.54694E-08; 1.61208E-08; 1.96173E-08; 2.05355E-08; 2.15341E-08; 2.18218E-08; 2.18218E-08; 2.2638E-08; 2.2638E-08; 2.44589E-08; 2.64658E-08; 3.00992E-08; 3.05025E-08; 3.39803E-08; 3.41633E-08; 3.64266E-08; 3.75268E-08; 3.84651E-08; 3.84651E-08; 4.67612E-08; 5.47024E-08; 5.77189E-08; 6.31394E-08; 7.14363E-08; 7.14363E-08; 7.67403E-08; 7.67486E-08; 7.79768E-08; 8.70024E-08; 8.70024E-08; 8.74639E-08; 8.96367E-08; 9.356E-08; 9.40117E-08; 9.62503E-08; 1.02884E-07; 1.0733E-07; 1.12301E-07; 1.12301E-07; 1.12301E-07; 1.12301E-07; 1.14502E-07; 1.1607E-07; 1.24686E-07; 1.24686E-07; 1.27609E-07; 1.28058E-07; 1.29414E-07; 1.36777E-07; 1.37094E-07; 1.40422E-07; 1.41141E-07; 1.41141E-07; 1.53404E-07; 1.53404E-07; 1.58901E-07; 1.58901E-07; 1.80894E-07; 1.8246E-07; 1.90637E-07; 1.90637E-07; 1.9416E-07; 1.99943E-07; 2.04806E-07; 2.09682E-07; 2.17491E-07; 2.20064E-07; 2.22278E-07; 2.23311E-07; 2.23311E-07; 2.29642E-07; 2.35949E-07; 2.37783E-07; 2.37783E-07; 2.37783E-07; 2.66351E-07; 2.77622E-07; 2.92868E-07; 3.22834E-07; 3.30795E-07; 3.30795E-07; 3.92431E-07; 4.25483E-07; 4.58135E-07; 4.58135E-07; 4.92079E-07; 4.92869E-07; 4.96976E-07; 5.00977E-07; 5.10717E-07; 5.11133E-07; 5.11133E-07; 5.22479E-07; 5.35838E-07; 5.87875E-07; 5.87875E-07; 6.1879E-07; 6.27937E-07; 6.3058E-07; 6.37002E-07; 6.41202E-07; 6.41202E-07; 7.22101E-07; 7.27533E-07; 7.4755E-07; 7.4755E-07; 7.4755E-07; 7.74384E-07; 7.90642E-07; 8.1219E-07; 8.1219E-07; 9.55002E-07; 9.71224E-07; 9.78722E-07; 1.26459E-06; 1.27797E-06; 1.28024E-06; 1.38118E-06; 1.38118E-06; 1.38875E-06; 1.4063E-06; 1.44056E-06; 1.50616E-06; 1.64319E-06; 1.6726E-06; 1.70661E-06; 1.81923E-06; 1.82524E-06; 1.82524E-06; 1.82524E-06; 1.85731E-06; 1.86122E-06; 1.8934E-06; 1.91586E-06; 2.03632E-06; 2.13324E-06; 2.13324E-06; 2.33348E-06; 2.39739E-06; 2.48086E-06; 2.61006E-06; 2.75279E-06; 2.82822E-06; 2.83743E-06; 3.02311E-06; 3.0727E-06; 3.17675E-06; 3.17675E-06; 3.26392E-06; 3.35E-06; 3.86E-06; 3.91E-06; 4.59E-06; 4.89786E-06; 5.11658E-06; 5.44125E-06; 5.44125E-06; 5.44125E-06; 5.44125E-06; 5.44125E-06; 5.64469E-06; 5.88383E-06; 6.24023E-06; 6.35222E-06; 6.37067E-06; 6.60785E-06; 6.60785E-06; 7.07624E-06; 7.09653E-06; 7.09653E-06; 7.09653E-06; 7.22118E-06; 7.23609E-06; 7.25528E-06; 7.34646E-06; 7.34646E-06; 7.34646E-06; 7.57334E-06; 8.286E-06; 8.31839E-06; 8.36077E-06; 8.44178E-06; 8.99992E-06; 9.19831E-06; 9.38833E-06; 9.38833E-06; 9.38833E-06; 9.38833E-06; 9.40447E-06; 9.40447E-06; 9.81332E-06; 9.81332E-06; 9.99907E-06; 9.99907E-06; 9.99907E-06; 1.03284E-05; 1.03284E-05; 1.03483E-05; 1.04678E-05; 1.10178E-05; 1.12361E-05; 1.1441E-05; 1.15142E-05; 1.2018E-05; 1.2018E-05; 1.2018E-05; 1.2018E-05; 1.23924E-05; 1.23924E-05; 1.23924E-05; 1.23924E-05; 1.23924E-05; 1.23924E-05; 1.23924E-05; 1.23924E-05; 1.24216E-05; 1.24922E-05; 1.26586E-05; 1.33797E-05; 1.4037E-05; 1.4186E-05; 1.45943E-05; 1.48379E-05; 1.57846E-05; 1.5899E-05; 1.6933E-05; 1.75331E-05; 1.77397E-05; 1.82998E-05; 1.86797E-05; 1.86873E-05; 1.90189E-05; 2.04715E-05; 2.05053E-05; 2.06152E-05; 2.15165E-05; 2.15165E-05; 2.1954E-05; 2.25879E-05; 2.25879E-05; 2.37814E-05; 2.37814E-05; 2.37814E-05; 2.37814E-05; 2.47228E-05; 2.47228E-05; 2.47228E-05; 2.55357E-05; 2.60335E-05; 2.68433E-05; 2.71833E-05; 2.7519E-05; 2.83746E-05; 2.87362E-05; 2.90026E-05; 3.16494E-05; 3.16494E-05; 3.17099E-05; 3.19601E-05; 3.27304E-05; 3.30033E-05; 3.35727E-05; 3.43431E-05; 3.43448E-05; 3.52714E-05; 3.61587E-05; 3.66572E-05; 3.67657E-05; 3.85799E-05; 3.88537E-05; 3.95518E-05; 4.10614E-05; 4.11754E-05; 4.12201E-05; 4.12201E-05; 4.12201E-05; 4.15381E-05; 4.29569E-05; 4.5096E-05; 4.5096E-05; 4.5096E-05; 4.55744E-05; 4.5665E-05; 4.70408E-05; 4.88287E-05; 4.88287E-05; 4.8969E-05; 4.8969E-05; 4.94957E-05; 5.35139E-05; 5.40931E-05; 5.56196E-05; 5.81986E-05; 5.90961E-05; 6.03948E-05; 6.03948E-05; 6.03948E-05; 6.09705E-05; 6.22576E-05; 6.46111E-05; 6.46111E-05; 6.77755E-05; 6.84303E-05; 6.87973E-05; 6.87973E-05; 6.88479E-05; 6.89072E-05; 7.02951E-05; 7.05658E-05; 7.35458E-05; 7.35458E-05; 7.39519E-05; 7.49816E-05; 7.60803E-05; 7.61386E-05; 7.63459E-05; 7.77945E-05; 7.90533E-05; 7.93787E-05; 7.93787E-05; 8.17239E-05; 8.23989E-05; 8.23989E-05; 8.37303E-05; 8.37303E-05; 8.4706E-05; 8.7308E-05; 8.7308E-05; 8.79057E-05; 8.99749E-05; 9.08979E-05; 9.11715E-05; 9.15224E-05; 9.49872E-05; 9.49872E-05; 9.70245E-05; 9.82249E-05; 9.84207E-05; 0.000100052; 0.00010408; 0.00010408; 0.000105939; 0.000106409; 0.000106905; 0.00010893; 0.000109896; 0.000109896; 0.000111243; 0.000112563; 0.000112563; 0.000112563; 0.000114299; 0.000114299; 0.000114299; 0.000116283; 0.000116283; 0.000119596; 0.000119596; 0.00011975; 0.00012828; 0.00012828; 0.00012828; 0.00012828; 0.00012828; 0.00012828; 0.00012828; 0.000129201; 0.000131849; 0.00013317; 0.00013317; 0.000135937; 0.000135937; 0.000135937; 0.000152048; 0.000152048; 0.000152048; 0.000152048; 0.000152477; 0.000155506; 0.000155506; 0.000156568; 0.000162905; 0.000162957; 0.000162957; 0.000162957; 0.000164841; 0.000164841; 0.000164954; 0.000164954; 0.000176051; 0.000177921; 0.000184282; 0.000184282; 0.000184282; 0.000184627; 0.000184627; 0.000184627; 0.000191468; 0.000191525; 0.000191525; 0.000191525; 0.000191525; 0.000191525; 0.000192533; 0.000199835; 0.000199835; 0.000200954; 0.000200954; 0.000200954; 0.000205369; 0.000207934; 0.000207934; 0.000207934; 0.000208842; 0.000211715; 0.000211715; 0.000212115; 0.000212115; 0.000212115; 0.000212538; 0.000218307; 0.00021852; 0.00021852; 0.00021852; 0.00021852; 0.000222874; 0.000226654; 0.000228174; 0.000228998; 0.000230356; 0.000230834; 0.000233291; 0.000233291; 0.000238931; 0.000238931; 0.00024066; 0.000264486; 0.000264486; 0.00027098; 0.000273438; 0.000287694; 0.000289696; 0.000289696; 0.000289696; 0.000289696; 0.000293755; 0.00029526; 0.00029526; 0.000303628; 0.000303628; 0.000303628; 0.000305088; 0.000310907; 0.000314116; 0.000314116; 0.000314116; 0.000327495; 0.000331462; 0.000340576; 0.000342927; 0.000342927; 0.000342927; 0.000343641; 0.000346335; 0.000346335; 0.000346335; 0.000348274; 0.000352283; 0.000352283; 0.000363293; 0.000372329; 0.000373168; 0.000376754; 0.000378463; 0.000378463; 0.000383334; 0.000389715; 0.000389715; 0.000390724; 0.000390724; 0.000399174; 0.000399337; 0.000399337; 0.000408384; 0.000409085; 0.000409085; 0.000409085; 0.000414488; 0.000414488; 0.000425129; 0.000425129; 0.00043; 0.00043; 0.000442538; 0.000443606; 0.000443606; 0.000444537; 0.000444537; 0.000444537; 0.000444537; 0.000462919; 0.000462919; 0.000474548; 0.000476382; 0.000476382; 0.000478185; 0.000478185; 0.000487761; 0.000487761; 0.000487761; 0.000490511; 0.000494926; 0.000494926; 0.000494926; 0.000510456; 0.000512201; 0.000520674; 0.000526046; 0.000529509; 0.000529509; 0.000529509; 0.000529509; 0.000529509; 0.000529509; 0.000529509; 0.000532986; 0.000533993; 0.000550314; 0.000550314; 0.000550314; 0.000561506; 0.000561506; 0.000561506; 0.000561506; 0.000561506; 0.000561506; 0.000561506; 0.000561506; 0.000561506; 0.000561506; 0.000561506; 0.000569442; 0.000570446; 0.000570446; 0.000576858; 0.000576858; 0.000576858; 0.000594372; 0.000594372; 0.000594372; 0.000611496; 0.000611496; 0.000613524; 0.000626622; 0.000641213; 0.000642682; 0.000649225; 0.000649225; 0.000658968; 0.000687187; 0.000687187; 0.000687187; 0.000687187; 0.000708271; 0.000708271; 0.000708271; 0.000717643; 0.000717643; 0.000717643; 0.00073254; 0.00073254; 0.00073397; 0.000738555; 0.000738555; 0.000756106; 0.000762501; 0.000803309; 0.000819147; 0.000819147; 0.000819147; 0.000819147; 0.000832991; 0.000832991; 0.000832991; 0.000871169; 0.000890514; 0.000890514; 0.000897813; 0.000905239; 0.000905239; 0.00092042; 0.000924184; 0.000924184; 0.000924184; 0.000924184; 0.000924184; 0.00093747; 0.000938554; 0.000938554; 0.000957252; 0.000971898; 0.000971898; 0.000971898; 0.000995318; 0.000995318; 0.000995318; 0.000998093; 0.001007192; 0.001007192; 0.00101974; 0.001031633; 0.001031633; 0.001031633; 0.001031633; 0.00103728; 0.00103728; 0.00104229; 0.00104229; 0.00104229; 0.00104229; 0.00104229; 0.001064041; 0.001077368; 0.001098231; 0.001098231; 0.001098231; 0.001098231; 0.001098231; 0.001098231; 0.001098231; 0.001098231; 0.001102114; 0.001109025; 0.001147586; 0.001165646; 0.001237503; 0.001237503; 0.001237503; 0.001244602; 0.001244602; 0.001244602; 0.00125303; 0.001273486; 0.001273486; 0.001273486; 0.001280982; 0.001286443; 0.001292715; 0.001292715; 0.001292715; 0.001292715; 0.001292715; 0.001373204; 0.001382307; 0.001383803; 0.001383803; 0.001409223; 0.001409223; 0.001420023; 0.001437683; 0.001437683; 0.001437683; 0.001437683; 0.001437683; 0.001470751; 0.001473238; 0.001503354; 0.001515958; 0.001515958; 0.001528817; 0.001542195; 0.001542195; 0.001542195; 0.001542195; 0.001542195; 0.001542195; 0.001559536; 0.001568179; 0.001584946; 0.001584946; 0.001592221; 0.001619289; 0.001619289; 0.001669843; 0.001692032; 0.001692032; 0.001692032; 0.001692032; 0.001692032; 0.001729382; 0.001729382; 0.001746946; 0.001746946; 0.001785294; 0.001823587; 0.001851657; 0.001851657; 0.001851657; 0.001851657; 0.001851657; 0.001851657; 0.001927015; 0.001927015; 0.001942125; 0.001945457; 0.001965048; 0.001965048; 0.002004838; 0.002018856; 0.002021586; 0.002021586; 0.002021586; 0.00208078; 0.00209086; 0.00209086; 0.002097336; 0.002105538; 0.002105538; 0.002124544; 0.002153132; 0.002186364; 0.002186364; 0.002186364; 0.002186364; 0.002186364; 0.002186364; 0.002186364; 0.002186364; 0.002186364; 0.002186364; 0.002207989; 0.002288402; 0.002288402; 0.002288402; 0.002288402; 0.002344654; 0.002365612; 0.002365612; 0.002365612; 0.002365612; 0.002365612; 0.002384787; 0.002384787; 0.00241361; 0.002424339; 0.002477555; 0.002477555; 0.002529033; 0.002552805; 0.002552805; 0.002552805; 0.002552805; 0.002552805; 0.002552805; 0.00259108; 0.00259108; 0.00259108; 0.002615448; 0.002626497; 0.002704618; 0.002704618; 0.002704618; 0.002749959; 0.002749959; 0.002749959; 0.002749959; 0.002764897; 0.002805141; 0.002814686; 0.002895654; 0.002937132; 0.002965441; 0.002965441; 0.002965441; 0.002965441; 0.002974541; 0.00298251; 0.003008508; 0.003035335; 0.003049332; 0.003091699; 0.003091699; 0.003164337; 0.003164337; 0.003164337; 0.003164337; 0.003164337; 0.003164337; 0.003164337; 0.003164337; 0.003164337; 0.003171676; 0.003171676; 0.003247904; 0.003346864; 0.003399953; 0.003399953; 0.003399953; 0.003399953; 0.003421721; 0.003428521; 0.003429608; 0.003429608; 0.003556863; 0.003556863; 0.003620572; 0.003620572; 0.003620572; 0.003620572; 0.003620572; 0.003620572; 0.003620572; 0.003620572; 0.00366663; 0.00366663; 0.00366663; 0.00366663; 0.003797557; 0.003799145; 0.003799145; 0.003799145; 0.003799145; 0.003799145; 0.003799145; 0.003843772; 0.003843772; 0.003843772; 0.003843772; 0.003917404; 0.003917404; 0.003917404; 0.003917404; 0.003917404; 0.003917404; 0.003917404; 0.004017831; 0.004034473; 0.004034473; 0.004050328; 0.004050328; 0.004064583; 0.004064583; 0.004064583; 0.004064583; 0.004064583; 0.004112715; 0.004112715; 0.004112715; 0.004112715; 0.004112715; 0.004112715; 0.004112715; 0.004112715; 0.004112715; 0.004112715; 0.004112715; 0.004112715; 0.004112715; 0.004112715; 0.004112715; 0.004112715; 0.004112715; 0.00427297; 0.00427297; 0.00427297; 0.00427297; 0.004277677; 0.004277677; 0.004277677; 0.004277677; 0.004342241; 0.004443815; 0.004508113; 0.004508113; 0.004527774; 0.004527774; 0.004527774; 0.004527774; 0.004587433; 0.004587433; 0.00472749; 0.00472749; 0.004794878; 0.004794878; 0.004794878; 0.004794878; 0.004794878; 0.004794878; 0.004794878; 0.004819941; 0.004826513; 0.004826513; 0.004826513; 0.004826513; 0.004826513; 0.004826513; 0.004826513; 0.004826513; 0.004842302; 0.004858012; 0.004858012; 0.005018493; 0.005018493; 0.005018493; 0.005018746; 0.005018746; 0.005018746; 0.005018746; 0.005018746; 0.005018746; 0.005119717; 0.005183861; 0.005183861; 0.005283701; 0.005309659; 0.005309659; 0.005309659; 0.005309659; 0.005309659; 0.00533568; 0.00533568; 0.00533568; 0.005471423; 0.005474359; 0.005489837; 0.005489837; 0.005489837; 0.005489837; 0.005489837; 0.005489837; 0.005489837; 0.005489837; 0.005489837; 0.005489837; 0.005489837; 0.005489837; 0.005489837; 0.005489837; 0.005489837; 0.005489837; 0.005489837; 0.005489837; 0.005489837; 0.005489837; 0.005489837; 0.005489837; 0.005489837; 0.005489837; 0.005489837; 0.005489837; 0.005540168; 0.005540168; 0.00557612; 0.005602044; 0.005637406; 0.005727091; 0.005727091; 0.005727091; 0.005759345; 0.005759345; 0.005759345; 0.005759345; 0.005759345; 0.005759345; 0.005759345; 0.00583168; 0.006097794; 0.006097794; 0.006097794; 0.006293804; 0.006293804; 0.006293804; 0.006293804; 0.006293804; 0.006293804; 0.006293804; 0.006293804; 0.006325982; 0.006333982; 0.006360534; 0.006387031; 0.006387031; 0.006387031; 0.006428665; 0.006498159; 0.006691346; 0.006905374; 0.006905374; 0.006905374; 0.006905902; 0.006956082; 0.007084241; 0.007084241; 0.007084241; 0.007084241; 0.007084241; 0.007084241; 0.007084241; 0.007124027; 0.007124027; 0.007124027; 0.007124027; 0.007124027; 0.007124027; 0.007124027; 0.007124027; 0.007193462; 0.007259032; 0.007398198; 0.00747008; 0.00747008; 0.00747008; 0.00747008; 0.007570417; 0.007669093; 0.007735856; 0.007735856; 0.007735856; 0.007735856; 0.007735856; 0.007808614; 0.007910399; 0.007984248; 0.008023835; 0.008023835; 0.008023835; 0.008023835; 0.008023835; 0.008023835; 0.008073514; 0.008073514; 0.008073514; 0.008074578; 0.008074578; 0.008074578; 0.008130044; 0.008251422; 0.008291903; 0.008291903; 0.008291903; 0.008291903; 0.008339399; 0.008417889; 0.008417889; 0.008442976; 0.008763761; 0.008783123; 0.008792057; 0.008792057; 0.008792057; 0.008792057; 0.008792057; 0.008792057; 0.008830685; 0.008830685; 0.008830685; 0.008830685; 0.008830685; 0.008830685; 0.008830685; 0.008830685; 0.008830685; 0.008830685; 0.008830685; 0.008830685; 0.008830685; 0.008830685; 0.008830685; 0.008878887; 0.008878887; 0.008966832; 0.009063603; 0.009063603; 0.009063603; 0.009063603; 0.009063603; 0.009375873; 0.009490702; 0.009490702; 0.009490702; 0.009501042; 0.009617903; 0.009617903; 0.009641857; 0.009713999; 0.009713999; 0.009713999; 0.009713999; 0.009713999; 0.009713999; 0.009713999; 0.009713999; 0.009713999; 0.009713999; 0.009713999; 0.009713999; 0.009713999; 0.009713999; 0.009713999; 0.009739961; 0.009758402; 0.009769945; 0.009769945; 0.009939284; 0.010008624; 0.010008624; 0.010204009; 0.010204009; 0.010582132; 0.010666187; 0.010666187; 0.010711456; 0.010711456; 0.010711456; 0.010711456; 0.010711456; 0.010711456; 0.010711456; 0.010711456; 0.01071212; 0.010773037; 0.010773037; 0.010873834; 0.011020497; 0.011020497; 0.011020497; 0.011020497; 0.011344548; 0.011483289; 0.011483289; 0.011483289; 0.011483289; 0.011680093; 0.011680093; 0.011680093; 0.011680093; 0.011680093; 0.011680093; 0.011680093; 0.011680093; 0.011680093; 0.011680093; 0.011680093; 0.011680093; 0.011680093; 0.011818128; 0.011828447; 0.011828447; 0.011828447; 0.011828447; 0.012431517; 0.012471613; 0.012471613; 0.012702125; 0.012702125; 0.012702125; 0.012702125; 0.012702125; 0.012702125; 0.012702125; 0.012702125; 0.012702125; 0.012702125; 0.012702125; 0.012702125; 0.012702125; 0.012702125; 0.012702125; 0.012702125; 0.012814799; 0.012898084; 0.012898084; 0.013191995; 0.013191995; 0.013191995; 0.013191995; 0.013191995; 0.013500973; 0.013658472; 0.013658472; 0.013658472; 0.013658472; 0.013658472; 0.013673014; 0.013673014; 0.013673014; 0.013673014; 0.013673014; 0.013673014; 0.013673014; 0.013673014; 0.013673014; 0.013673014; 0.013673014; 0.013673014; 0.013673014; 0.013673014; 0.013673014; 0.014008968; 0.014008968; 0.014017607; 0.014017607; 0.014581118; 0.014673626; 0.014673626; 0.014673626; 0.014793833; 0.014793833; 0.014793833; 0.014793833; 0.014793833; 0.014793833; 0.014793833; 0.014793833; 0.014814654; 0.014920717; 0.014920717; 0.014933549; 0.014933549; 0.014960289; 0.014960289; 0.01511229; 0.015216287; 0.015216287; 0.015495108; 0.015495108; 0.015868748; 0.015868748; 0.015868748; 0.015868748; 0.015868748; 0.015868748; 0.015868748; 0.015868748; 0.015868748; 0.015868748; 0.015868748; 0.01594275; 0.01594275; 0.016403783; 0.016494193; 0.016494193; 0.016494193; 0.016494193; 0.016516247; 0.016934448; 0.016934448; 0.016934448; 0.016934448; 0.016934448; 0.016934448; 0.016934448; 0.016934448; 0.016934448; 0.016934448; 0.016934448; 0.016934448; 0.016934448; 0.016934448; 0.016934448; 0.016934448; 0.016934448; 0.017012176; 0.017080348; 0.017462429; 0.017462429; 0.017571108; 0.017836585; 0.017836585; 0.018058813; 0.018082158; 0.018082158; 0.018082158; 0.018082158; 0.018082158; 0.018082158; 0.018082158; 0.018082158; 0.018082158; 0.018082158; 0.018082158; 0.018082158; 0.018296947; 0.018454606; 0.018454606; 0.018454606; 0.018454606; 0.018454606; 0.018454606; 0.018785885; 0.018785885; 0.018785885; 0.018785885; 0.018785885; 0.019003325; 0.019003325; 0.019003325; 0.01905383; 0.019101643; 0.019101643; 0.019101643; 0.019101643; 0.019101643; 0.019101643; 0.019101643; 0.019101643; 0.019101643; 0.019101643; 0.019101643; 0.019101643; 0.019101643; 0.019101643; 0.019354615; 0.019354615; 0.019763902; 0.019763902; 0.019772188; 0.019993904; 0.019993904; 0.019993904; 0.019993904; 0.019993904; 0.020092482; 0.020092482; 0.020286502; 0.020286502; 0.020286502; 0.020286502; 0.020286502; 0.020286502; 0.020286502; 0.020532505; 0.021195953; 0.021195953; 0.021577659; 0.021577659; 0.021577659; 0.021577659; 0.021577659; 0.021577659; 0.021577659; 0.021577659; 0.021577659; 0.021577659; 0.021583706; 0.021583706; 0.021608177; 0.021672722; 0.021672722; 0.021867523; 0.021973138; 0.022322588; 0.022322588; 0.02232443; 0.022381906; 0.022781536; 0.022781536; 0.022781536; 0.022781536; 0.022781536; 0.022781536; 0.022781536; 0.022781536; 0.022781536; 0.022855473; 0.023164175; 0.023554204; 0.02416254; 0.02416254; 0.02416254; 0.02416254; 0.02416254; 0.02416254; 0.02416254; 0.024164729; 0.024472749; 0.024534144; 0.024866771; 0.024923793; 0.025250189; 0.025293899; 0.025293899; 0.025293899; 0.025293899; 0.025293899; 0.025293899; 0.025293899; 0.025293899; 0.025293899; 0.025293899; 0.025293899; 0.025293899; 0.025293899; 0.025293899; 0.025293899; 0.025293899; 0.025293899; 0.025293899; 0.025293899; 0.025293899; 0.025664691; 0.025862621; 0.025931707; 0.025931707; 0.025931707; 0.026363748; 0.026488375; 0.026570205; 0.026570205; 0.026570205; 0.026570205; 0.026570205; 0.026570205; 0.026570205; 0.026570205; 0.026570205; 0.026570205; 0.026570205; 0.027244796; 0.027315564; 0.027802657; 0.027802657; 0.027967621; 0.027987566; 0.027987566; 0.027987566; 0.027987566; 0.027987566; 0.027987566; 0.02864302; 0.028678477; 0.028678477; 0.028678477; 0.029207912; 0.029253535; 0.029253535; 0.029253535; 0.029253535; 0.029253535; 0.029253535; 0.029253535; 0.029253535; 0.029253535; 0.029253535; 0.029253535; 0.029253535; 0.029253535; 0.029253535; 0.029253535; 0.029452491; 0.029839004; 0.029839004; 0.029839004; 0.029891443; 0.029891443; 0.029891443; 0.029892714; 0.030363787; 0.03051219; 0.03051219; 0.03051219; 0.030586585; 0.030586585; 0.030586585; 0.030586585; 0.030586585; 0.031211575; 0.031211575; 0.031328059; 0.031856689; 0.031856689; 0.031897232; 0.031897232; 0.031897232; 0.031897232; 0.031897232; 0.031910149; 0.031910149; 0.031910149; 0.031910149; 0.031910149; 0.031910149; 0.031910149; 0.031910149; 0.032154626; 0.032505933; 0.032505933; 0.032505933; 0.032654354; 0.033200699; 0.033200699; 0.033200699; 0.033200699; 0.033200699; 0.033200699; 0.033200699; 0.033200699; 0.033200699; 0.033200699; 0.033200699; 0.033200699; 0.033200699; 0.033200699; 0.033200699; 0.033404329; 0.033818938; 0.033818938; 0.033818938; 0.034497092; 0.034497092; 0.034497092; 0.034497092; 0.034497092; 0.034497092; 0.034497092; 0.034497092; 0.034497092; 0.034497092; 0.034497092; 0.034497092; 0.034497092; 0.034497092; 0.034497092; 0.034497092; 0.035193734; 0.035193734; 0.035997707; 0.035997707; 0.035997707; 0.035997707; 0.035997707; 0.035997707; 0.035997707; 0.035997707; 0.035997707; 0.036455672; 0.036455672; 0.036723833; 0.036814455; 0.037537713; 0.037537713; 0.037537713; 0.037537713; 0.037537713; 0.037537713; 0.038113986; 0.038729899; 0.039190585; 0.039190585; 0.039190585; 0.039190585; 0.039255511; 0.040061724; 0.040061724; 0.040842053; 0.040842053; 0.040842053; 0.040842053; 0.040867636; 0.040867636; 0.04087033; 0.040977079; 0.041228955; 0.041730782; 0.042214592; 0.042214592; 0.042334794; 0.042334794; 0.042334794; 0.042334794; 0.042334794; 0.042334794; 0.042448083; 0.042448083; 0.043403366; 0.044003499; 0.044021206; 0.044021206; 0.044021206; 0.044590526; 0.045754675; 0.045754675; 0.045754675; 0.045754675; 0.045754675; 0.045831028; 0.046418646; 0.047480696; 0.047480696; 0.047480696; 0.047480696; 0.047480696; 0.047567182; 0.047567182; 0.047860091; 0.047860091; 0.047860091; 0.048592368; 0.048843869; 0.049079335; 0.049079335; 0.049079335; 0.049079335; 0.049079335; 0.049719566; 7.24324E-14; 7.24324E-14; 9.57688E-14; 1.17995E-10; 3.13844E-10; 3.96697E-10; 5.39387E-10; 5.18175E-09; 8.55395E-09; 9.38403E-09; 5.90894E-08; 9.41444E-08; 2.18887E-07; 7.97027E-07; 7.97027E-07; 1.6672E-06; 2.07903E-06; 1.30337E-05; 5.45154E-05; 5.53149E-05; 7.62115E-05; 0.000174123; 0.000256166; 0.000316483; 0.000542727; 0.000661322; 0.000750828; 0.000814893; 0.000852103; 0.001035956; 0.001378359; 0.001418349; 0.001912868; 0.002071885; 0.003142976; 0.003142976; 0.003221858; 0.003464046; 0.004744683; 0.005170194; 0.005706402; 0.005706402; 0.005746988; 0.005746988; 0.005746988; 0.005942312; 0.007406677; 0.007430106; 0.007430106; 0.007551343; 0.008649985; 0.009866811; 0.010218505; 0.012365549; 0.012424848; 0.012837635; 0.012965437; 0.013048123; 0.013048123; 0.01583074; 0.016451431; 0.017281597; 0.019733916; 0.019985347; 0.023366236; 0.028631356; 0.028631356; 0.030214015; 0.038592104; 0.039038774; 0.042159308; 0.046086413; 0.046539637; 0.046539637; 0.046539637; 0.046539637; 0.046539637; 0.047144967; 0.047144967; 0.04784791; 0.048499615; 2.387E-10; 2.82377E-10; 2.82377E-10; 2.82377E-10; 5.99115E-10; 1.00657E-09; 1.52834E-07; 2.518E-07; 4.25419E-07; 1.85565E-06; 2.02092E-06; 2.02092E-06; 2.25704E-06; 1.02959E-05; 1.33058E-05; 1.93187E-05; 4.82229E-05; 6.64195E-05; 6.64195E-05; 0.000123361; 0.000123361; 0.000123361; 0.000123361; 0.000260373; 0.000260373; 0.000260373; 0.000260373; 0.000260373; 0.000295953; 0.000295953; 0.000295953; 0.000295953; 0.000298828; 0.000302964; 0.000322707; 0.000464206; 0.000464206; 0.00059134; 0.000677873; 0.000677873; 0.000682138; 0.000791519; 0.000794906; 0.000809526; 0.000893605; 0.001095817; 0.001123998; 0.00116046; 0.00116046; 0.001235122; 0.0013408; 0.001711353; 0.002204642; 0.002700298; 0.002700298; 0.003037876; 0.003257843; 0.003392529; 0.004027607; 0.004557578; 0.004557578; 0.004893284; 0.005179189; 0.005179189; 0.005217316; 0.00725712; 0.00725712; 0.007726977; 0.008330974; 0.008809818; 0.009275693; 0.009275693; 0.009671446; 0.010070022; 0.010070022; 0.010070022; 0.010679052; 0.011758064; 0.012029772; 0.012937576; 0.012937576; 0.013180445; 0.01453643; 0.016337942; 0.020807336; 0.021884383; 0.021994375; 0.021994375; 0.022119508; 0.02241371; 0.023551172; 0.02437729; 0.025149543; 0.028016665; 0.029117837; 0.029117837; 0.029117837; 0.030286323; 0.03070605; 0.03070605; 0.032932137; 0.035360383; 0.035811799; 0.036359958; 0.036359958; 0.03852878; 0.038765657; 0.04304101; 0.048804748 0.048804747 |
