# Supplementary material for: Molecular Mechanism of Salvia miltiorrhiza Bunge in Treating Cerebral Infarction
Source: Evid Based Complement Alternat Med. 2022 Mar 29;2022:5992394. doi: 10.1155/2022/5992394 (PMC8983215; doi:10.1155/2022/5992394)
Supplement: Supplementary Materials — Table S1: details of active compounds and targets. Table S2: known therapeutic targets in cerebral infarction. Table S3: the details of the PPI network. Table S4: the details of the GO analysis. Table S5: the KEGG pathway enrichment analysis. [file 5992394.f1.zip › 5992394.f1/Table S5- The KEGG pathway enrichment analysis (1).doc]

**Table S7: The KEGG pathway enrichment analysis**

| **Description** | **p.adjust** |
| --- | --- |
| Lipid and atherosclerosis; Chemical carcinogenesis - receptor activation; Hepatitis B; IL-17 signaling pathway; Prostate cancer; Endocrine resistance; Small cell lung cancer; Kaposi sarcoma-associated herpesvirus infection; PI3K-Akt signaling pathway; Colorectal cancer; Toxoplasmosis; Bladder cancer; Human cytomegalovirus infection; Epstein-Barr virus infection; T cell receptor signaling pathway; Th17 cell differentiation; Hepatitis C; Apoptosis; TNF signaling pathway; Measles; Leishmaniasis; AGE-RAGE signaling pathway in diabetic complications; Chagas disease; FoxO signaling pathway; JAK-STAT signaling pathway; HIF-1 signaling pathway; Estrogen signaling pathway; Fluid shear stress and atherosclerosis; Prolactin signaling pathway; Proteoglycans in cancer; Non-small cell lung cancer; Breast cancer; Pertussis; Pancreatic cancer; Chronic myeloid leukemia; Human T-cell leukemia virus 1 infection; Relaxin signaling pathway; C-type lectin receptor signaling pathway; Neuroactive ligand-receptor interaction; PD-L1 expression and PD-1 checkpoint pathway in cancer; Platinum drug resistance; cAMP signaling pathway; Non-alcoholic fatty liver disease; Cellular senescence; EGFR tyrosine kinase inhibitor resistance; MicroRNAs in cancer; Endometrial cancer; cGMP-PKG signaling pathway; Yersinia infection; Inflammatory bowel disease; Gastric cancer; Th1 and Th2 cell differentiation; Thyroid hormone signaling pathway; Melanoma; Apoptosis - multiple species; p53 signaling pathway; Osteoclast differentiation; Hepatocellular carcinoma; Insulin resistance; Salmonella infection; Thyroid cancer; Human immunodeficiency virus 1 infection; Cholinergic synapse; Tuberculosis; Transcriptional misregulation in cancer; Coronavirus disease - COVID-19; Cell cycle; Acute myeloid leukemia; Pathogenic Escherichia coli infection; Alzheimer disease; Epithelial cell signaling in Helicobacter pylori infection; Calcium signaling pathway; Pathways of neurodegeneration - multiple diseases; Viral carcinogenesis; Malaria; Glioma; Toll-like receptor signaling pathway; B cell receptor signaling pathway; Legionellosis; ErbB signaling pathway; Neurotrophin signaling pathway; Allograft rejection; Human papillomavirus infection; Shigellosis; Adipocytokine signaling pathway; Influenza A; Progesterone-mediated oocyte maturation; NF-kappa B signaling pathway; NOD-like receptor signaling pathway; Intestinal immune network for IgA production; Cocaine addiction; Adrenergic signaling in cardiomyocytes; AMPK signaling pathway; Focal adhesion; MAPK signaling pathway; Regulation of lipolysis in adipocytes; VEGF signaling pathway; Viral myocarditis; Rheumatoid arthritis; Dopaminergic synapse; African trypanosomiasis; Amoebiasis; Renal cell carcinoma; Central carbon metabolism in cancer; Parathyroid hormone synthesis, secretion and action; Adherens junction; Oxytocin signaling pathway; Serotonergic synapse; Diabetic cardiomyopathy; Sphingolipid signaling pathway; Growth hormone synthesis, secretion and action; Rap1 signaling pathway; Platelet activation; Autoimmune thyroid disease; GnRH signaling pathway; Insulin signaling pathway; Choline metabolism in cancer; Asthma; Signaling pathways regulating pluripotency of stem cells; Fc epsilon RI signaling pathway; Renin secretion; Amphetamine addiction; Cushing syndrome; Aldosterone-regulated sodium reabsorption; Regulation of actin cytoskeleton; Graft-versus-host disease; Ras signaling pathway; Oocyte meiosis; Type II diabetes mellitus; Vascular smooth muscle contraction; Gap junction; Longevity regulating pathway; Ovarian steroidogenesis; Chemokine signaling pathway; Salivary secretion; Phospholipase D signaling pathway; Hedgehog signaling pathway Arachidonic acid metabolism | 7.65E-15; 4.02E-14; 3.79E-13; 2.72176E-12; 3.44694E-12; 3.44694E-12; 2.31205E-11; 4.39879E-11; 4.39879E-11; 1.25355E-10; 2.24193E-10; 2.24193E-10; 3.77816E-10; 6.08574E-10; 1.00453E-09; 1.33643E-09; 1.33643E-09; 2.05458E-09; 2.05458E-09; 2.50759E-09; 5.41555E-09; 6.39684E-09; 7.73304E-09; 1.17838E-08; 1.49243E-08; 1.49243E-08; 2.00236E-08; 2.11158E-08; 2.54258E-08; 3.15882E-08; 3.15882E-08; 3.68432E-08; 4.80791E-08; 4.80791E-08; 4.80791E-08; 6.65682E-08; 7.34815E-08; 7.87989E-08; 1.07282E-07; 1.99842E-07; 4.35069E-07; 4.46047E-07; 4.99155E-07; 5.23844E-07; 7.96996E-07; 8.58712E-07; 8.58712E-07; 1.01694E-06; 1.08485E-06; 1.99832E-06; 2.44331E-06; 2.59565E-06; 2.82966E-06; 4.12512E-06; 4.2485E-06; 4.42816E-06; 4.4346E-06; 7.10752E-06; 8.94281E-06; 9.40921E-06; 9.40921E-06; 1.01343E-05; 1.22308E-05; 1.26613E-05; 2.32647E-05; 2.42145E-05; 2.47164E-05; 2.47164E-05; 2.80342E-05; 2.85524E-05; 3.13775E-05; 3.13775E-05; 3.56529E-05; 3.64406E-05; 4.67239E-05; 4.6959E-05; 4.6959E-05; 8.25943E-05; 9.53263E-05; 0.00010184; 0.000119094; 0.000145714; 0.000147313; 0.000188948; 0.00026157; 0.00026157; 0.000266328; 0.000337403; 0.000440787; 0.000454179; 0.000454179; 0.000535029; 0.000776766; 0.000776766; 0.000776953; 0.000883122; 0.001027413; 0.001099987; 0.001169866; 0.001289549; 0.001514685; 0.001855797; 0.001995153; 0.002109985; 0.002206364; 0.002208192; 0.002995597; 0.003273812; 0.003353134; 0.003795262; 0.003795262; 0.004043938; 0.004595203; 0.005215101; 0.006743917; 0.007366616; 0.008303548; 0.008861906; 0.008861906; 0.012183773; 0.012617861; 0.012617861; 0.012648152; 0.013939284; 0.017443416; 0.019495956; 0.0236517; 0.023699281; 0.024411266; 0.026350397; 0.0271466; 0.027980338; 0.031152504; 0.031152504; 0.031669794; 0.038206648; 0.038853911 0.0481897861368518 |
